# Systematic Review and Meta-Analysis of Observational Studies on the Effectiveness and Safety of Ustekinumab among Patients with Inflammatory Bowel Disease in Eastern and Western Countries

**DOI:** 10.3390/jcm12051894

**Published:** 2023-02-27

**Authors:** He Zhou, Fang Wang, Jian Wan, Song Su, Yanting Shi, Xiaofei Li, Tong Wu, Jie Liang

**Affiliations:** 1 State Key Laboratory of Cancer Biology, National Clinical Research Center for Digestive Diseases, Xijing Hospital of Digestive Diseases, Fourth Military Medical University, Xi’an 710032, China; 2 Department of Gastroenterology, The First Medical Center of Chinese PLA General Hospital, Beijing 100853, China

**Keywords:** inflammatory bowel disease, effectiveness, meta-analysis, ustekinumab

## Abstract

Background: Ustekinumab (UST) is an IL12/23 inhibitor utilized for altering inflammatory responses in inflammatory bowel disease (IBD). Clinical trials and case reports suggested that the effectiveness and safety of UST may differ among IBD patients in Eastern and Western countries. However, related data have not been systematically reviewed and analyzed. Methods: This systematic review and meta-analysis of the safety and effectiveness of UST in IBD included relevant literature from the Medline and Embase databases. The main outcomes were clinical response, clinical remission, endoscopic response, endoscopic remission, and adverse events in IBD. Results: We analyzed 49 real-world studies, most of which included patients with biological failure (89.1% CD and 97.1% UC). In UC patients, clinical remission rates were 34% at 12 weeks, 40% at 24 weeks, and 37% at 1 year. In CD patients, clinical remission rates were 46% at 12 weeks, 51% at 24 weeks, and 47% at 1 year. Clinical remission rates of CD patients were 40% at 12 weeks and 44% at 24 weeks in Western countries, versus 63% and 72% in Eastern countries, respectively. Conclusion: UST is an effective drug for IBD with a promising safety profile. Although no RCTs have been performed in Eastern countries, the effectiveness of UST on CD patients is not inferior to that in Western countries based on the existing data.

## 1. Introduction

Inflammatory bowel disease (IBD), including ulcerative colitis (UC) and Crohn’s disease (CD), is a group of non-specific chronic inflammatory diseases with unclear etiology in the gastrointestinal tract. Over recent years, there has been an increasing number of biologic medications used for IBD treatment, such as monoclonal antibodies or inhibitors to tumor necrosis factor (TNF)-α, interleukin (IL)-12/23, adhesion molecules, and Janus kinase (JAK). Ustekinumab (UST) is a fully human IgG antibody that inhibits the early-stage and chronic inflammation in IBD by antagonizing the p40 subunit, shared by IL-12 and IL-23, which blocks the IL-12/23 inflammatory pathways. While UST was initially approved to treat psoriasis in 2008, the US Food and Drug Administration (FDA) and the European Union Medicines Agency (EMA) have approved UST to alter the inflammatory responses in IBD. However, UST was approved only for CD remedy but not for UC in some Asian countries.

Both UNITI and UNIFI clinical trials have confirmed the effect of UST in the induction of remission and the remission duration of IBD compared to placebo [1,2]. Due to the strict inclusion criteria, the randomized clinical trials (RCT) mentioned above may not be sufficient to represent real-world IBD cases [1,2,3,4,5,6]. Due to the differences between Eastern and Western countries, the efficacy and safety of UST in the treatment of IBD may be inconsistent. In particular, only a few reports have noted UST clinical practice among IBD patients in Eastern countries, and no studies stated the differences in UST’s efficacy and safety between Eastern and Western ethnicities. Therefore, in this trial, we systematically evaluated the effectiveness and safety of UST on IBD patients by incorporating real-world data in Eastern and Western countries to guide physicians in clinical practice.

## 2. Materials and Methods

### 2.1. Information Sources and Search Strategies

This meta-analysis was conducted and reported according to the guidelines of the Cochrane Handbook for Systematic Reviews and the Preferred Reporting Items for Systematic Reviews and Meta-Analyses (PRISMA) (Table S2) [7,8]. A systematic search was performed in the MEDLINE, Embase, and PubMed databases from inception to September 2022. Search terms based on subject headings and keywords included, but were not limited to, the following: ulcerative colitis, Crohn’s disease, inflammatory bowel disease, and ustekinumab. A review of titles and abstracts was independently performed by the primary co-authors (ZH and WF). Disagreements between the investigators were solved by consensus with a third author (SS).

### 2.2. Eligibility Criteria and Study Selection

Studies that fulfilled all the following criteria were included for analysis: (1) study design as case series or retrospective and prospective cohort studies; (2) the study subjects were patients with IBD; (3) real-world observational studies, which assessed the effectiveness and/or safety outcome measures of UST in clinical practice. Exclusion criteria were: (1) patients with no confirmed diagnosis of IBD; (2) RCT study; (3) studies with incomplete, duplicate, or unusable data.

Two investigators (ZH and WF) strictly followed the inclusion and exclusion criteria while independently performing literature screening and data extraction. In case of discrepancies in the literature screening process, the third investigator (SYT) participated in the screening and decided whether to include the report in question after comprehensive consultation. Then, a full-text review and a search of the reference lists of the screened results were performed. The information gathered for each eligible study included author, year of publication, country, indication, sample size, mean age, age at onset, male sex, and mean disease duration.

### 2.3. Outcome Measures

The primary outcome measure was clinical remission. Secondary outcome measures included clinical response, corticosteroid-free (CS)-free clinical remission, mucosal healing, endoscopic response, endoscopic remission, and safety. These outcomes were used to analyze the differences between Eastern and Western countries, as well as the composition of biological agents for the treatment of IBD.

Among CD patients, CD activity index (CDAI) [9] was used to assess clinical remission (CDAI < 150 points) and clinical response (CDAI ≥ 100 points). The Harvey–Bradshaw Index (HBI) [10] was used to assess corticosteroid-free (CS)-free clinical remission (HBI ≤ 3 points without the use of any steroid preparation). The simple endoscopic score for CD (SES-CD) [11] was used to assess mucosal healing (SES-CD = 0 points) and endoscopic response (a decrease in SES-CD ≥ 8 points or SES-CD ≤ 4 points). Among UC patients, partial Mayo score (PMS) was used to assess clinical remission (PMS ≤ 2) and clinical response (PMS ≥ 3 points from the baseline score); Mayo endoscopic subscore (MES) was used to assess endoscopic response (MES ≤ 1) [12].

By using a broader definition, Western and Eastern countries in this study were defined by cultural, economic, and ethnicity factors rather than geographics [13]. In this study, countries, including Spain, Germany, the US, the UK, Sweden, Northlands, France, Canada, Ireland, Finland, Belgium, Italy and Slovenia, were categorized as Western countries; the others, including China, Japan, Republic of Korea and Saudi Arabia, were categorized as Eastern countries.

### 2.4. Study Quality Assessment

The quality of the included studies was assessed according to the MINORS scale developed by Karem Slim for non-randomized controlled interventional studies [14], which includes a total of 12 indicators for MINORS evaluation, each with a score of 0–2, with the highest score being 24 points. The scoring method was as follows: 0, not reported; 1, reported but insufficient information; 2, reported and provided sufficient information; 0–8, low-quality literature; 9–16, medium quality; 17–24, high quality. Two investigators (ZH and WF) independently scored the quality of the reports. In case of inconsistency, a third researcher (SYT) made a comprehensive judgment, finally reaching a consensus.

### 2.5. Statistical Analysis

Meta-analyses were performed to combine clinical response, clinical remission, CS-free clinical remission, mucosal healing, endoscopic response, and endoscopic remission rates using the R software (version 3.2.2; R Foundation; Vienna, Austria) with the “meta” package (version 4.3-2). Weighted mean clinical response, CS-free clinical remission, mucosal healing, endoscopic response, endoscopic remission rate, and corresponding 95% confidence intervals (CIs) were calculated using the DerSimonian–Laird random effects model to account for between-study heterogeneity [15]. Study heterogeneity was determined using the I^2^ statistic and the Q-statistic, where *p* < 0.05 suggested statistically significant heterogeneity. *p* > 0.1 and I^2^ ≤ 50% indicated that the heterogeneity between the study groups was not significant, and the fixed effects model was adopted; *p* ≤ 0.1 and I^2^ > 50% indicated a significant heterogeneity, and the random effects model was used. For publication bias assessment, funnel plots, Begg’s adjusted rank correlation test, and Egger’s regression test were used. We also performed subgroup analysis for results with high heterogeneity.

## 3. Results

### 3.1. Study Selection and Characteristics

**Figure 1:**
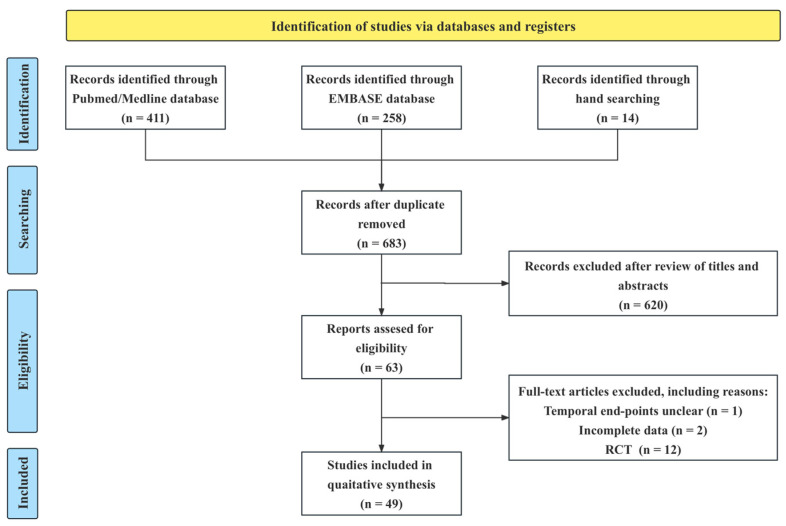
Flow diagram of the search process for referred reporting items for systematic reviews and meta-analyses.

As shown in Figure 1, 683 hits were identified through our database search (PubMed, 411; Embase, 258; hand search, 14). After reading the titles and abstracts, 620 irrelevant studies were removed. Following screening and full-text review, 63 relevant reports were identified and retrieved for detailed evaluation. Among these, 49 studies [3,4,12,16,17,18,19,20,21,22,23,24,25,26,27,28,29,30,31,32,33,34,35,36,37,38,39,40,41,42,43,44,45,46,47,48,49,50,51,52,53,54,55,56,57,58,59,60,61] including 8176 patients (CD, n = 4731; UC, n = 3445) treated with UST from real-world data that met the inclusion criteria were finally included in the analysis (Table 1).

For IBD patients administered UST, we focused on whether they had used biological agents previously and the types used. In all CD patients, excluding cases with unclear first- or second-line use, 3494 cases reported previous use of biological agents (89.1%), while only 10.9% were biologics-naive. Among CD cases with biological failure, 75.9% were administered infliximab, 65.1% were administered adalimumab, 31.2% were treated with vedolizumab, and 39% were administered other drugs. In UC patients, excluding cases with unclear first- or second-line use, 515 cases reported previous use of biological agents (97.1%), and only 2.9% were biologics-naive. For UC cases with biological failure, 84.2% were administered infliximab, and 63.6% had vedolizumab. Other medications and disease-related status prior to UST treatment were analyzed (Table S1), including anti-TNF therapy, CS therapy, immunosuppressant (IMS) therapy, concomitant IMS therapy, concomitant CS therapy, fistulizing disease, and prior surgery.

The included studies, which were all non-RCTs, were evaluated using the MINORS scale for non-RCT studies. The grading of the quality of evidence of the studies ranged from 16 to 19, as shown in Table 1.

### 3.2. Clinical Outcomes at Induction

Clinical response at induction in patients administered UST was assessed in 6 studies of UC [12,51,55,56,57,59] and 17 studies of CD [3,16,17,19,20,22,23,30,31,33,34,39,40,43,45,46,50]. In UC, the pooled estimate of the clinical response rate was 61% (95% CI: 55–67%; I^2^ = 34.62%—Figure S1, Table 2), while in CD, the pooled estimate of the clinical response rate was 55% (95% CI: 46–65%; I^2^ = 93.71%—Figure S2A, Table 2).

**Figure 2:**
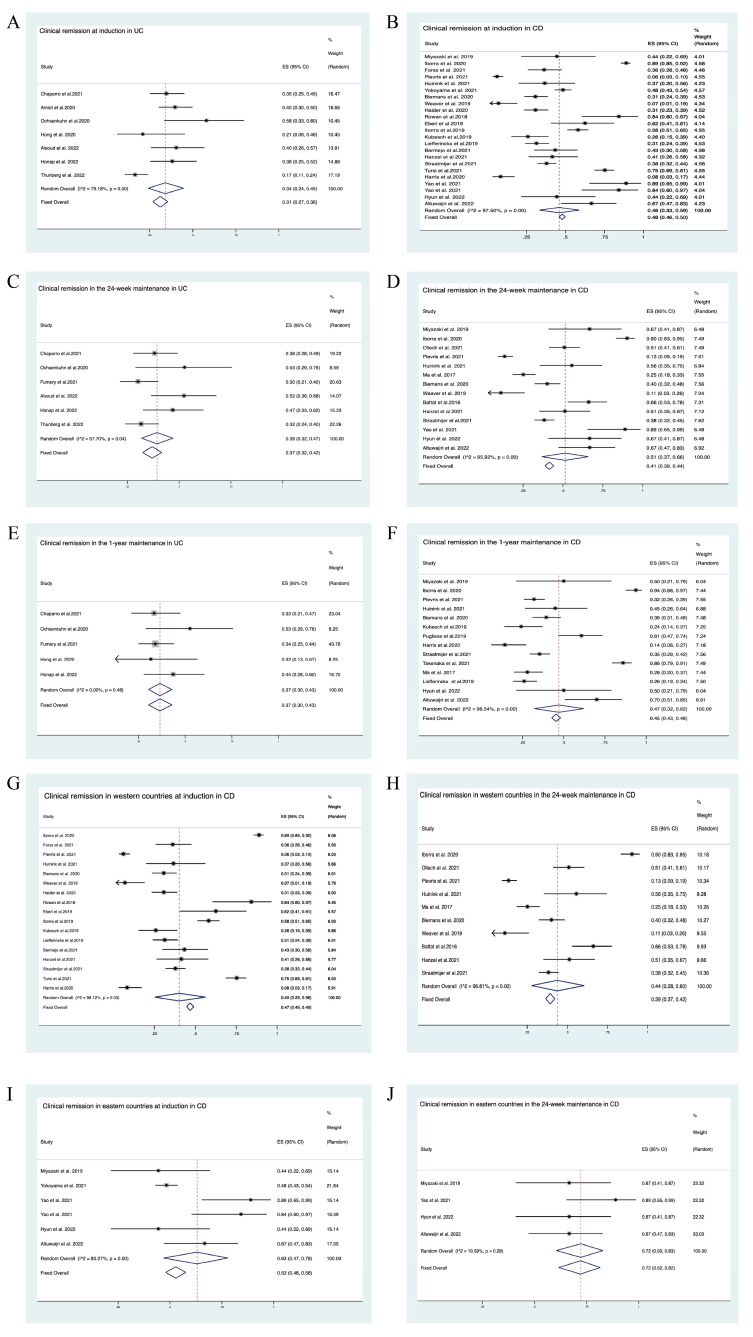
Meta-analysis of studies that assessed the clinical remission at induction and during the 24-week and 1-year maintenance periods. The size of each square represents the weight assigned to each study based on sample size. Error bars represent 95% CIs. Diamonds represent the point estimate of the average study rates; the lateral tips of the diamonds denote 95% CIs. (**A**) Clinical remission at induction in UC. (**B**) Clinical remission at induction in CD. (**C**) Clinical remission at 24 weeks of maintenance in UC. (**D**) Clinical remission at 24 weeks of maintenance in CD. (**E**) Clinical remission at the end of the 1-year maintenance period in UC. (**F**) Clinical remission at the end of the 1-year maintenance period in CD. (**G**) Clinical remission in Western countries at induction in CD. (**H**) Clinical remission in Western countries at 24 weeks in CD. (**I**) Clinical remission in Eastern countries at induction in CD. (**J**) Clinical remission in Eastern countries at 24 weeks in CD.

Clinical remission at induction in patients treated with UST was assessed in 7 studies of UC [12,51,52,54,57,58,59] and 23 studies of CD [3,18,19,20,22,26,27,29,30,31,32,33,34,40,41,42,43,44,46,48,49,50,61]. In UC, the pooled estimate of the clinical remission rate was 34% (95% CI: 24–45%; I^2^ = 79.18%—Figure 2A, Table 2), while in CD, the pooled estimate of the clinical remission rate was 46% (95% CI: 33–59%; I^2^ = 97.50%—Figure 2B, Table 2).

CS-free remission at induction in patients treated with UST was assessed in 5 studies of UC [52,55,56,58,59] and 6 studies of CD [19,33,34,38,40,50]. In UC, the pooled estimate of the CS-free remission rate was 38% (95% CI: 23–55%; I^2^ = 86.11%—Figure S3A, Table 2), while in CD, the pooled estimate of the CS-free remission rate was 44% (95% CI: 32–56%; I^2^ = 88.83%—Figure S4A, Table 2).

### 3.3. Clinical and Endoscopic Outcomes at Maintenance

Clinical response at 24 weeks of maintenance in patients treated with UST was assessed in 9 studies of CD [16,17,33,34,37,39,44,45,50], where the pooled estimate of the clinical response rate was 66% (95%s CI: 53–78%; I^2^ = 92.90%—Figure S2B, Table 2).

Clinical remission at 24 weeks of maintenance in patients administered UST was assessed in 6 studies of UC [12,51,53,54,58,59] and 14 studies of CD [18,24,26,30,32,33,34,37,39,42,43,48,49,50]. In UC, the pooled estimate of the clinical remission rate was 39% (95% CI: 32–47%; I^2^ = 57.70%—Figure 2C, Table 2). In CD, the pooled estimate of the clinical response rate was 51% (95% CI: 37–66%; I^2^ = 95.92%—Figure 2D, Table 2).

CS-free remission at the end of the 24-week maintenance period in patients administered UST was assessed in 5 studies of UC [51,53,55,59] and 6 studies of CD [21,33,34,37,41,50]. In UC, the pooled estimate of the CS-free remission rate was 40% (95% CI: 30–50%; I^2^ = 62.78%—Figure S3B, Table 2). In CD, the pooled estimate of the CS-free remission rate was 49% (95% CI: 39–59%; I^2^ = 85.39%—Figure S4B, Table 2).

Clinical response at the end of the 1-year maintenance period in patients administered UST was assessed in 10 studies of CD [16,17,22,25,28,31,33,34,40,50]. In CD, the pooled estimate of the clinical response rate was 55% (95% CI: 47–62%; I^2^ = 75.96%—Figure S2C, Table 2).

Clinical remission at the end of the 1-year maintenance period in patients administered UST was assessed in 5 studies of UC [51,53,54,57,59] and 14 studies of CD [4,18,22,30,31,32,33,34,39,40,47,48,49,50]. In UC, the pooled estimate of the clinical remission rate was 37% (95% CI: 30–43%; I^2^ = 0.00%—Figure 2E, Table 2). In CD, the pooled estimate of the clinical response rate was 47% (95% CI: 32–62%; I^2^ = 96.54%—Figure 2F, Table 2).

CS-free remission at the end of the 1-year maintenance period in patients administered UST was assessed in 6 studies of UC [51,53,55,57,59,60] and 7 studies on CD [16,18,25,28,33,34,50]. In UC, the pooled estimate of the CS-free remission rate was 37% (95% CI: 24–51%; I^2^ = 91.09%—Figure S3C, Table 2). In CD, the pooled estimate of the CS-free remission rate was 52% (95% CI: 41–62%; I^2^ = 87.16%—Figure S4C, Table 2).

The endoscopic response at the end of the 1-year maintenance period in patients administered UST was assessed in 9 studies of CD [18,35,36,37,38,39,43,45,48]. In CD, the pooled estimate of the endoscopic response rate was 65% (95% CI: 57–71%; I^2^ = 45.54%—Figure S5, Table 2).

Endoscopic remission at the end of the 1-year maintenance period in patients administered UST was assessed in 9 studies of CD [4,18,37,39,42,43,45,47,49]. In CD, the pooled estimate of the endoscopic remission rate was 29% (95% CI: 18–40%; I^2^ = 80.35%—Figure S6, Table 2).

Mucosal healing in the 1-year maintenance period in patients treated with UST was assessed in 7 studies of CD [21,28,30,35,36,41,48]. In CD, the pooled estimate of the clinical response rate was 31% (95% CI: 19–44%; I^2^ = 79.88%—Figure S7, Table 2).

### 3.4. Clinical Outcomes by Geographic Location

Analysis by geographic location showed variable combined clinical response and remission rates among patients with CD at induction and at week 24. Among studies conducted in Western countries, the response rate was 50% at induction (95%CI: 40–61%; I^2^ = 94.05%—Figure S8), while remission rates were 40% at induction (95%CI: 25–56%; I^2^ = 98.12%—Figure 2G) and 44% at week 24 (95% CI 28–60%; I^2^ = 96.81%—Figure 2H). Among the studies conducted in Eastern countries, the response rate was 75% at induction (95%CI: 43–97%; I^2^ = 94.26%—Figure S9), and remission rates were 63% at induction (95%CI: 47–78%; I^2^ = 80.27%—Figure 2I) and 72% at week 24 (95% CI: 60–83%; I^2^ = 19.59%—Figure 2J).

### 3.5. Safety

Safety outcomes were reported in 15 studies, while adverse events were assessed in 5 studies of UC [51,52,53,62] and 10 CD studies [6,16,17,18,21,30,32,33,35,38,61,63,64]. In UC, the pooled estimate of the incidence rate of total adverse events was 5% (95% CI: 3–8%; I^2^ = 13.69%—Figure S10), while in CD, the pooled estimate of the incidence rate of total adverse events was 11% (95% CI: 6–18%; I^2^ = 91.38%—Figure S11).

### 3.6. Clinical Remission of Biologics-Naive and Biologics-Experienced Patients

Biologics-naive and biologics-experienced patients were reported in 5 studies [19,23,46,48]. In biologics-naive patients, the pooled estimate of the incidence rate of clinical remission was 97% (95% CI: 80–100%; I^2^ = 0%—Figure S12), while in biologics-experienced patients, the value was 58% (95% CI: 48–68%; I^2^ = 60.08%—Figure S13).

### 3.7. Publication Bias and Subgroup Analysis

Publication bias analysis was performed on the data of more than 6 included studies, including clinical response at induction in CD, clinical remission at induction in CD, CS-free remission at induction in CD, clinical response at the end of the 24-week maintenance period in CD, clinical remission at the end of the 24-week maintenance period in CD, CS-free remission at the end of the 24-week maintenance period in CD, clinical response at the end of the 1-year maintenance period in CD, clinical remission at the end of the 1-year maintenance period in CD, endoscopic response at the end of the 1-year maintenance period in CD, endoscopic remission at the end of the 1-year maintenance period in CD, mucosal healing at the end of the 1-year maintenance period in CD, and adverse events in CD cases.

As the funnel plot, Begg’s adjusted rank correlation test, and Egger’s regression test showed heterogeneity in clinical response at induction in CD (Figure S14), we conducted subgroup analysis according to regional differences (Europe/America or Asia). However, the obtained results were not significant (*p* > 0.05), indicating that regional differences were not the cause of this heterogeneity (Figure S15). There was no significant heterogeneity in other outcomes (Figures S16–S26).

## 4. Discussion

Based on real-world data, this meta-analysis revealed that UST is an effective drug for both CD and UC with a promising safety profile. Though the effectiveness was better in biologics-naïve patients, almost 90% of patients with biological failure were treated with ustekinumab in the real world. Remarkably, the effectiveness of this biologic for IBD patients in Eastern countries was not inferior to that of patients in Western countries.

**Figure 3:**
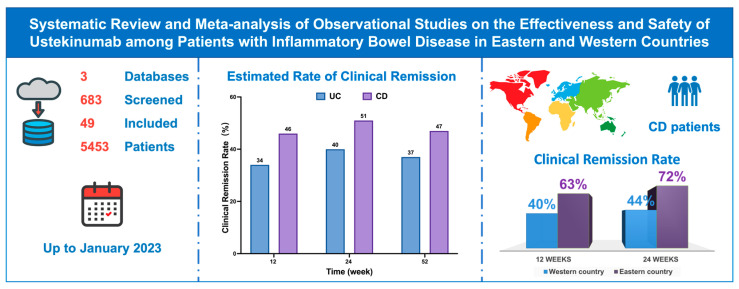
Clinical remission rates significantly differed among CD patients enrolled in Western and Eastern countries. Based on the data obtained from 49 real-world observational studies, this meta-analysis determined the estimated rates of IBD patients administered UST for clinical remission at 12 weeks, 24 weeks and 48 weeks before January 2023. Especially among CD patients, there was a huge difference in clinical remission between Western and Eastern countries.

Our results revealed that clinical remission rates at the end of the 12-week induction period and the 1-year maintenance period could be indirectly compared with findings for UST reported by the UNIFI and UNITI trials [1,2]. Although both the UNIFI and UNITI trials were RCTs, they had critical inclusion and exclusion criteria, which might not fully represent real-world circumstances. This single-arm meta-analysis of aggregate data from 49 real-world observational studies of UST in IBD showed pooled estimate rates in CD patients treated with UST of 55% for clinical response and 46% for clinical remission at induction. In contrast, at the end of the 1-year maintenance period, the pooled estimate rates of clinical response, clinical remission, CS-free remission, endoscopic response, and endoscopic remission were 55%, 47%, 52%, 65%, and 29%, respectively. In the UNIFI trial, the pooled estimate rates were 61.8% for clinical response and 15.5% for clinical remission at induction. In contrast, at the end of the 1-year maintenance period, the pooled estimate rates of clinical response, clinical remission, CS-free remission, and endoscopic response were 71%, 43.8%, 42%, and 51%, respectively. These suggested that the clinical outcomes in CD were even better in real-world data than in RCT data (Figure 3).

In UC patients, pooled estimate rates were 61% for clinical response and 34% for clinical remission at induction. In contrast, at the end of the 1-year maintenance period, the pooled estimate rates of clinical remission and CS-free remission were 37% and 37%, respectively. In the UNITI trial, pooled estimate rates were 37.8% for clinical response and 20.9% for clinical remission at induction. In contrast, at the end of the 1-year maintenance period, the pooled estimate rates of clinical response and clinical remission were 59.4% and 53.1%, respectively. The pooled rates for clinical outcomes were also higher in UC patients compared with those reported by the UNITI RCT. Regardless, 89.1% of patients failed to respond to biologics in the real-world study.

Anti-tumor necrosis factor-α (TNF) therapy is a first-line biological drug for the treatment of moderate-to-severe inflammatory bowel disease, refractory to conventional therapy (mesalamine, steroids, and immunosuppressants) [65,66]. About one-third of Crohn’s disease patients not receiving biologics may not respond to induction therapy, and among those who respond, up to 45% lose response over time [67,68]. Several new therapies have been approved in the past five years for moderate-to-severe UC and CD, including vedolizumab and UST. A randomized, double-blind, phase III SEAVUE multicenter study conducted a head-to-head clinical trial of UST and adalimumab, revealing no significant difference in endoscopic remission rates between UST and adalimumab at week 52; however, UST showed better improvement in SES-CD score [69]. Additionally, a multicenter, real-world study found that fewer failures after treatment with biologics were associated with higher rates of mucosal healing in CD with UST among 1113 CD patients [70]. Combining the results of our investigation, nearly 90% of patients administered UST as second-line treatment still achieved good efficacy, suggesting that UST is safe and effective as either first- or second-line treatment.

Even though no RCTs of UST have been conducted in China, there are a few reports from small centers on the use of UST in China. In 2021, three real-world studies assessed the use of UST in the treatment of Crohn’s disease in China. Among these studies, Yao et al. [43] evaluated the short-term efficacy of UST in 18 CD patients. They revealed that 72.2% (13/18) of patients achieved clinical remission and 77.8% (14/18) of cases had a clinical response in week 8; 88.9% (16/18) of patients achieved clinical remission, and 94.4% (17/18) had a clinical response in week 16/20; at week 16/20, the endoscopic remission and response rates were 28.6% (4/14) and 78.6% (11/14), respectively. According to Yao et al., the clinical remission and response rates of UST in the induction and maintenance phases were higher than those obtained in this analysis. In another study, the latter group [44] explored whether different concentrations of ustekinumab affected clinical and endoscopic outcomes in patients with refractory Crohn’s disease. They included 19 eligible patients, reporting clinical response, clinical remission, endoscopic response, and endoscopic remission rates of 89.5%, 84.2%, 42.2%, and 73.7% at week 16/20 after UST initiation, respectively. A study by Gu et al. [71] reviewed the use of UST in three cases of moderate-to-severe CD in a single center, further confirming the efficacy and safety of UST. Besides the abovementioned clinical studies, two case reports [72,73] performed in China also confirmed the efficacy and safety of UST in the treatment of CD. In the meantime, our IBD research center also conducted a national real-world study of UST. Our unpublished results showed pooled estimate rates for clinical response of 62.9% and 83.6% at 8 and 20 weeks, respectively, and clinical remission rates of 54.2% and 60.9%, respectively. Taken together, it seems UST showed a good effect among Chinese IBD patients, but more prolonged and real-world observations of the effectiveness and safety of UST in China are still required. This study also indicated similar effectiveness of UST among IBD patients in both Eastern and Western countries. Specifically, the clinical remission rate at 12 and 24 weeks among patients in Eastern countries was significantly higher than that in Western countries.

Considering possible differences in the efficacy of UST targeting IL12/23 in Western and Eastern countries, it was shown that the IL-23/IL-17 axis plays an important role in IBD development [74]. IL-23 is essential for the expansion and maintenance of the Th17 response [75,76]. Moreover, IL-23R is expressed in various cells and can directly activate a subset of macrophages, monocytes, natural killer cells, and dendritic cells, which secrete IL-17 [77,78]. Currently, there are differences in various studies assessing the correlation between IL12/23 and IBD, while results from Eastern and Western regions were also different. In the Finnish population, IL-23 polymorphisms were shown to increase CD susceptibility [79], while in Swedish cases, IL-23 was associated with UC but not CD [80]. An opposite finding was obtained in a study by Magyaril et al. [81]. These authors indicated that the IL-23 gene variants appeared to increase CD susceptibility but not UC susceptibility in the Hungarian population. Song et al. [82] found that IL-23/IL-17 pathway genes were locally and systemically upregulated in Chinese IBD patients. In a study, Zhao et al. [83] found that IL-12B gene (rs6887695 and rs2288831) polymorphisms increased the risk of UC in the Chinese population. Nevertheless, discrepant results were also reported. A study revealed that of the three IL-23R SNPs studied, Arg381Gln and Val362Ile were present but were not associated with IBD in the Chinese population [84]. In Japan, Keiko et al. [85] could not confirm the associations of candidate genetic variations in the IL-23R and ATG16L1 genes with the 5p13.1 locus. All the above data suggest different correlations between IL-12/23 and IBD among studies and races. Therefore, there may be differences in the efficacy of UST in targeting IL-12/23 among IBD patients in Western and Eastern countries, which is the point of this meta-analysis.

The present meta-analysis also had several limitations, including potential publication bias. The bias may be related to the included reports being real-world studies. Because real-world studies do not include a control group for comparison, only reporting single-group data, we only performed an analysis of pooled rates, which could introduce bias. Real-world data may be less stringent than RCT data, which are obtained by rigorous data collection and quality control of data integrity. However, real-world data provide greater insights into the effectiveness of UST in heterogeneous and more complex patients, which is also more representative of clinical practice.

In conclusion, this study reviewed and analyzed the literature, including all real-world studies, which showed the effectiveness of UST among CD patients in Eastern countries is not inferior to that in Western countries. Though UST has only been approved for CD treatment in China, this analysis proved the efficacy and safety of UST in UC treatment, which might provide novel insights into the treatment of UC patients in China.

## Figures and Tables

**Table 1:** Study characteristics and patient demographics in the studies included in the systematic literature review.

Study	Country and Region	IBD Type	Sample Size, N	Mean Age, Years (±SD, IQR)	Age at Onset, %			Male Sex,%	Mean Disease Duration, Years (±SD, IQR)	MINORS Scale
					<17	17–40	>40			
Kopylov et al. 2020 [16]	Europe and Canada	CD	142	35 (26–49)	NR	NR	NR	38.7	10 (5–17)	16
Khorrami et al. 2016 [17]	Spain	CD	116	37 (28–48)	20.7	69	10.3	37.1	10 (6–17)	16
Iborra et al. 2020 [18]	Spain	CD	407	45.28 (34.86–55.93)	NR	NR	NR	48	11.06 (5.7–19.03)	18
Iborra et al. 2019 [19]	Spain	CD	305	43.7 (34.4, 53.6)	NR	NR	NR	49	11.7 (5.6, 18.8)	16
Bermejo et al. 2022 [20]	Spain	CD	53	45 ± 12	NR	NR	NR	49.1	14 ± 6.4	19
Hoffmann et al. 2019 [21]	Germany	CD	57	43.0 (21–68)	7	70	23	52.6	43 (21–68)	17
Kubesch et al. 2019 [22]	Germany	CD	106	39.5(19–73)	NR	NR	NR	41.5	11 (2–39)	16
Thomann et al. 2020 [23]	Germany	CD	72	38.9 (14.2)	NR	NR	NR	37.5	NR	19
Ollech et al. 2021 [24]	US	CD	506	35.8 (27.2–51.4)	33.6	60	4.5	NR	NR	17
Dalal et al. 2020 [25]	US	CD	64	42.2	NR	NR	NR	39.1	15.5	19
Weaver et al. 2019 [26]	US	CD	56	24.2 (11.7)	NR	NR	NR	43	NR	19
Haider et al. 2020 [27]	US	CD	143	42.2 (18–83)	NR	NR	NR	44.1	14.3 (1–38)	18
Garg et al. 2022 [28]	US	CD	78	37.6 ± 13.5	32.1	55.1	12.8	52.6	13.3 ± 7.9	18
Forss et al. 2021 [29]	Sweden	CD	114	40 (31–54)	15	66	19	53	NR	19
Plevris et al. 2021 [30]	UK	CD	216	39.0 (28.8–51.8)	NR	NR	NR	NR	9.9 (6.0–16.5)	19
Harris et al. 2020 [31]	UK	CD	84	41.6 (14.7)	NR	NR	NR	46.9	12.3 (8.9)	17
Huinink et al. 2021 [32]	Netherlands	CD	31	37 (30–48)	13	77.3	9.7	35.5	NR	19
Biemans et al. 2020 [33]	Netherlands	CD	221	38.2 (29.3–52.2)	NR	NR	NR	39.8	12.3 (7.5–19.3)	18
Straatmijer et al. 2021 [34]	Netherlands	CD	252	NR	NR	NR	NR	39.7	15 (10–22)	17
Wils et al. 2017 [35]	France	CD	88	32.5 (25.8–39.3)	NR	NR	NR	27	11.8 (7.7–17.1)	17
Wils et al. 2016 [36]	France	CD	122	33.8 (27.5–43.9)	NR	NR	NR	29	11.5 (6.9–17.1)	16
Battat et al. 2016 [37]	Canada	CD	62	NR	33.9	53.2	12.6	38.7	NR	17
Greenup et al. 2017 [38]	Canada	CD	73	37 (30–50)	NR	NR	NR	38	17 (10–23)	16
Ma et al. 2017 [39]	Canada	CD	104	44.6 (32.2–57.5)	NR	NR	NR	43.3	13.8 (9.1–22.9)	18
Rowan et al. 2018 [61]	Ireland	CD	19	36.2 (25.4–40.8)	NR	NR	NR	15.8	18.9 (5.9–23.2)	17
Eberl et al. 2019 [3]	Finland	CD	48	42.2 ± 14.9	NR	NR	NR	54.2	13.9 ± 10.3	16
Liefferinckx et al. 2019 [40]	Belgium	CD	152	41 (19–74)	3.3	67.8	27.7	30.9	NR	19
Pugliese et al. 2019 [4]	Italy	CD	64	41.75 (20.3–72.3)	NR	NR	NR	35.7	10 (0.50–33.8)	16
Tursi et al. 2021 [41]	Italy	CD	194	48 (38–58)	NR	NR	NR	55.7	13 (7–22)	19
Hanzel et al. 2021 [42]	Slovenia	CD	41	48 (31–55)	NR	NR	NR	NR	16 (7–26)	18
Yao et al. 2021 [43]	China	CD	18	30.5 (26.0, 38.0)	NR	NR	NR	66.7	6.5 (1.9, 10.0)	16
Yao et al. 2021 [45]	China	CD	127	31.0 ± 11.3	NR	NR	NR	57.5	NR	18
Yao et al. 2021 [44]	China	CD	19	29.1 ± 9.1	0	89.5	10.5	57.9	5.5 ± 4.7	18
Yokoyama et al. 2021 [46]	Japan	CD	339	37.2 (13.4)	NR	NR	NR	67.3	11.0 (9.1)	19
Takenaka et al. 2021 [47]	Japan	CD	143	36 (26–46)	NR	NR	NR	71	10 (4– 15)	17
Miyazaki et al. 2019 [48]	Japan	CD	47	42 (35–49)	NR	NR	NR	51.1	15.3 (10.5–24.5)	17
Hyun et al. 2022 [49]	Republic of Korea	CD	18	34.4 (26.1–40.8)	NR	NR	NR	43.8	19.7 (17.1–24.6)	18
Altuwaijri et al. 2022 [50]	Saudi Arabia	CD	30	34.2 ± 17.9	NR	NR	NR	50	10.6 ± 4.9	18
Chaparro et al. 2021 [51]	Spain	UC	95	47(16)	NR	NR	NR	44	NR	17
Amiot et al. 2020 [52]	France	UC	103	39.3 (29.1–52.3)	7.8	66	26.2	60.2	7.6 (3.6–12.9)	18
Fumery et al. 2021 [53]	France	UC	103	39.3 (29.1–52.3)	7.8	66	26.2	60.2	7.6 (3.6–12.9)	16
Ochsenkuhn et al. 2020 [54]	Germany	UC	19	46 (26–81)	NR	NR	NR	55	5 (2–15)	16
Chiappetta et al. 2021 [55]	Italy	UC	68	30 (2–65)	NR	NR	NR	63	NR	17
Dalal et al. 2021 [56]	US	UC	108	39 (30–56)	NR	NR	NR	43.5	9 (4–16)	18
Hong et al. 2020 [57]	US	UC	19	42.7 ± 17.0	NR	NR	NR	47.4	9.6 ± 8.4	16
Alsoud et al. 2022 [12]	Belgium	UC	42	40 (30–53)	NR	NR	NR	43	8 (4–15)	18
Thunberg et al. 2022 [58]	Sweden	UC	133	38 (28–48)	NR	NR	NR	60	7 (3–12)	19
Honap et al. 2022 [59]	UK	UC	110	40 (29–59)	NR	NR	NR	59	7 (3–13)	18
Pilon et al. 2022 [60]	Canada	UC	2645	44.0 ± 15.9	NR	NR	NR	52.6	NR	17

NR, not reported; UK, United Kingdom; US, United States.

**Table 2:** Summary of the main findings in patients with ulcerative colitis and Crohn’s disease.

			UC Patients		CD Patients
		N	Effect Size	N	Effect Size
		Studies		Studies	
Clinical response					
	Week 12	6	61% (95% CI: 55–67%; I^2^ = 34.62%)	17	55% (95% CI: 46–65%; I^2^ = 93.71%)
	Week 24			8	66% (95% CI: 53–78%; I^2^ = 92.90%)
	Week 52			9	55% (95% CI: 47–62%; I^2^ = 75.96%)
Clinical remission					
	Week 12	7	34% (95% CI: 24–45%; I^2^ = 79.18%)	23	46% (95% CI: 36–59%; I^2^ = 97.50%)
	Week 24	6	40% (95% CI:30–50%; I^2^ = 62.78%)	14	51% (95% CI: 37–66%; I^2^ = 95.92%)
	Week 52	5	37% (95% CI: 30–43%; I^2^ = 0.00%)	10	47% (95% CI: 32–62%; I^2^ = 96.54%)
CS-free remission					
	Week 12	5	38% (95% CI: 23–55%; I^2^ = 86.11%)	6	44% (95% CI: 32–56%; I^2^ = 88.83%)
	Week 24	5	38% (95% CI: 28–48%; I^2^ = 69.87%)	8	49% (95% CI: 39–59%; I^2^ = 85.39%)
	Week 52	6	37% (95% CI: 24–51%; I^2^ = 91.09%)	7	52% (95% CI: 41–62%; I^2^ = 87.16%)
Endoscopic response					
	Week 52			9	65% (95% CI: 57–71%; I^2^ = 45.54%)
Endoscopic remission					
	Week 52			9	29% (95% CI: 18–40%; I^2^ = 80.35%)
Mucosal healing					
	Week 52			7	31% (95% CI: 19–44%; I^2^ = 79.88%)
Adverse events		5	5% (95% CI: 3–8%; I^2^ = 13.69%)	10	11% (95% CI: 6–18%; I^2^ = 91.38%)

CS, corticosteroid.

## Data Availability

The data presented in this study are available on request from the corresponding author.

## Supplementary Materials

The following supporting information can be downloaded at https://www.mdpi.com/article/10.3390/jcm12051894/s1: Table S1, Information of patient medication in studies included in the systematic literature review; Table S2, Cochrane Handbook for Systematic Reviews and the Preferred Reporting Items for Systematic Reviews and Meta-Analyses (PRISMA) guidelines; Figure S1, Clinical response at induction in ulcerative colitis; Figure S2, Clinical response in Crohn’s disease; Figure S3, CS-free remission in ulcerative colitis; Figure S4, CS-free remission in Crohn’s disease; Figure S5, Endoscopic response in the 1-year maintenance period in Crohn’s disease; Figure S6, Endoscopic remission in the 1-year maintenance period in Crohn’s disease; Figure S7, Mucosal healing in the 1-year maintenance period in Crohn’s disease; Figure S8, Clinical response in Western countries in Crohn’s disease; Figure S9, Clinical response in Eastern countries in Crohn’s disease; Figure S10, Adverse events in ulcerative colitis; Figure S11, Adverse events in Crohn’s disease; Figure S12, Clinical remission in biologics-naive patients; Figure S13, Clinical remission in biologics-experienced patients; Figure S14, Publication bias of clinical response at induction in Crohn’s disease; Figure S15, Clinical response rates by geographic location at induction in Crohn’s disease; Figure S16, Publication bias of clinical remission at induction in Crohn’s disease; Figure S17, Publication bias of CS-free remission at induction in Crohn’s disease; Figure S18, Publication bias of clinical response in the 24-week maintenance period in Crohn’s disease; Figure S19, Publication bias of clinical remission in the 24-week maintenance period in Crohn’s disease; Figure S20, Publication bias of CS-free remission in the 24-week maintenance period in Crohn’s disease; Figure S21, Publication bias of clinical response in the 1-year maintenance period in Crohn’s disease; Figure S22, Publication bias of clinical remission in the 1-year maintenance period in Crohn’s disease; Figure S23, Publication bias of endoscopic response in the 1-year maintenance period in Crohn’s disease; Figure S24, Publication bias of endoscopic remission in the 1-year maintenance period in Crohn’s disease; Figure S25, Publication bias of mucosal healing in the 1-year maintenance period in Crohn’s disease; Figure S26, Publication bias of adverse events in Crohn’s disease.
