# Supplementary material for: Systematic Review and Meta-Analysis of Observational Studies on the Effectiveness and Safety of Ustekinumab among Patients with Inflammatory Bowel Disease in Eastern and Western Countries
Source: J Clin Med. 2023 Feb 27;12(5):1894. doi: 10.3390/jcm12051894 (PMC10004158; doi:10.3390/jcm12051894)
Supplement: Supplementary file 1 [file jcm-12-01894-s001.zip › jcm-2227959-supplementary.pdf]

## Content

Supplementary Table S1: Information of patient medication from studies included in the systematic literature review ........ 3

Supplementary Table S2: Cochrane Handbook for Systematic Reviews and the Preferred Reporting Items for Systematic Reviews and Meta-Analyses (PRISMA) guidelines ........ 7

Supplementary Figure S1: Clinical response at the induction in ulcerative colitis ........ 10

Supplementary Figure S2: Clinical response in Crohn's disease ........ 11

Supplementary Figure S3: CS-free remission in ulcerative colitis ........ 14

Supplementary Figure S4: CS-free remission in Crohn's disease ........ 17

Supplementary Figure S5: Endoscopic response in the 1-year maintenance in Crohn's disease ........ 20

Supplementary Figure S6: Endoscopic remission in the 1-year maintenance in Crohn's disease ........ 21

Supplementary Figure S7: Mucosal healing in the 1-year maintenance in Crohn's disease ........ 22

Supplementary Figure S8: Clinical response in Western countries in Crohn's disease ........ 23

Supplementary Figure S9: Clinical response in Eastern countries in Crohn's disease ........ 24

Supplementary Figure S10: Adverse events in ulcerative colitis ........ 25

Supplementary Figure S11: Adverse events in Crohn's disease ........ 26

Supplementary Figure S12: Clinical remission in biological native patients ........ 27

Supplementary Figure S13: Clinical remission in biological experienced patients ........ 28

Supplementary Figure S14: Publication bias of clinical response at induction in Crohn's disease ........ 29

Supplementary Figure S15: Clinical response rates by geographic location at induction in Crohn's disease ........ 32
Supplementary Figure S16: Publication bias of clinical remission at induction in Crohn's disease ........ 33
Supplementary Figure S17: Publication bias of cs-free remission at induction in Crohn's disease ........ 36
Supplementary Figure S18: Publication bias of clinical response in the 24-week maintenance in Crohn's disease ........ 39
Supplementary Figure S19: Publication bias of clinical remission in the 24-week maintenance in Crohn's disease ........ 42
Supplementary Figure S20: Publication bias of cs-free remission in the 24-week maintenance in Crohn's disease ........ 45
Supplementary Figure S21: Publication bias of clinical resopone in the 1-year maintenance in Crohn's disease ........ 48
Supplementary Figure S22: Publication bias of clinical remission in the 1-year maintenance in Crohn's disease ........ 51
Supplementary Figure S23: Publication bias of endoscopic response in the 1-year maintenance in Crohn's disease ........ 54
Supplementary Figure S24: Publication bias of endoscopic remission in the 1-year maintenance in Crohn's disease ........ 57
Supplementary Figure S25: Publication bias of mucosal healing in the 1-year maintenance in Crohn's disease ........ 60
Supplementary Figure S26: Publication bias of adverse events in Crohn's disease ........ 63

Supplementary Table S1: Information of patient medication from studies included in the systematic literature review

| Study | Prior Biologics, % | | | | | Prior anti-TNF therapy, % | Prior CS therapy, % | Prior IMS therapy,% | | | | | Concomitant IMS therapy,% | | | | Concomitat CS therapy, % | Fistulizing disease, % | Prior surgery, % |
|---|---|---|---|---|---|---|---|---|---|---|---|---|---|---|---|---|---|---|---|
| | Biologics naive | Infliximab | Adalimumab | Vedolizumab | other | | | Thiopurines | Methotrexate | Tacrolimus | Azathioprine | other | Azathioprine | Methotrexate | Thiopurines | other | | | |
| Kopylov et al. 2020 [16] | 0 | 100 | 95 | NR | 11 | 100 | NR | NR | 44.7 | 2.6 | NR | NR | 5.3 | 5.3 | NR | NR | 57.8 | NR | 44.7 |
| Khorrami et al. 2016 [17] | 6.9 | 90.5 | 93.1 | NR | 24.1 | NR | NR | 95.7 | 59.5 | 8.6 | NR | 4.3 | NR | NR | NR | 36.2 | 31.9 | 50 | 48.3 |
| Hoffmann et al. 2019 [21] | 5.3 | NR | NR | NR | NR | 94.7 | 63.2 | NR | NR | NR | NR | NR | NR | NR | NR | 5.3 | 35.1 | NR | NR |
| Miyazaki et al. 2019 [48] | 12.88 | 80.9 | 46.8 | NR | NR | 87.2 | NR | NR | NR | NR | NR | NR | 38.3 | NR | NR | NR | NR | 70.2 | 80.9 |
| Biemans et al. 2020 [33] | 0 | NR | NR | NR | NR | 100 | NR | NR | NR | NR | NR | NR | NR | NR | NR | 23.5 | 11.8 | 10.6 | 22.4 |
| Iborra et al. 2020 [18] | 3.5 | NR | NR | 22 | NR | 96 | 33 | NR | 20 | NR | 71 | 6 | NR | NR | NR | NR | NR | NR | 55 |
| Ollech et al. 2021 [24] | 7.3 | NR | NR | 46.4 | NR | 92.7 | 60.9 | 51.8 | 45.5 | NR | NR | NR | NR | NR | NR | NR | NR | NR | NR |

| | | | | | | | | | | | | | | | | | | | |
|---|---|---|---|---|---|---|---|---|---|---|---|---|---|---|---|---|---|---|---|
| Dalal et al. 2020 [25] | 0 | NR | NR | NR | NR | 100 | 31.3 | NR | NR | NR | NR | NR | NR | NR | NR | NR | NR | 37.5 | 64.1 |
| Forss et al. 2021 [29] | NR | NR | NR | NR | NR | NR | 32 | NR | NR | NR | NR | NR | NR | NR | NR | 23 | 18 | NR | NR |
| Plevris et al. 2021 [30] | NR | NR | NR | NR | NR | NR | NR | NR | NR | NR | NR | NR | NR | NR | NR | 25.5 | 40.7 | 17.1 | 55.1 |
| Takenaka et al. 2021 [47] | 54 | 46 | 31 | NR | NR | NR | NR | NR | NR | NR | NR | NR | NR | NR | NR | 48 | 6 | NR | 36 |
| Huinink et al. 2021 [32] | NR | NR | NR | 64.5 | NR | NR | NR | NR | NR | NR | NR | NR | NR | NR | NR | 6.5 | 29 | 29 | 64.5 |
| Yokoyama et al. 2021 [46] | NR | 76.7 | 50.6 | 0.4 | NR | NR | 38.3 | NR | NR | NR | NR | NR | NR | NR | NR | NR | NR | 19.5 | 45.1 |
| Wils et al. 2017 [35] | 0 | 97 | 90 | NR | NR | 100 | NR | 93 | 66 | NR | NR | NR | 9 | 6 | NR | NR | 15 | NR | 64 |
| Weaver et al. 2019 [26] | 36 | NR | NR | 39 | NR | 64 | NR | 32 | 32 | NR | NR | NR | NR | NR | NR | NR | NR | 35 | NR |
| Haider et al. 2020 [27] | 8.4 | NR | NR | 36.4 | NR | 91.6 | NR | NR | 35.7 | NR | NR | NR | NR | NR | NR | NR | NR | 23.9 | 65.7 |
| Wils et al. 2016 [36] | 0 | 97 | 91 | NR | 40 | 100 | NR | NR | NR | 4 | NR | 16 | NR | 6 | 9 | 15 | 16 | NR | 62 |
| Battat et al. 2016 [37] | 1.6 | NR | NR | NR | NR | 98.4 | NR | NR | NR | NR | NR | NR | NR | 9.7 | 16.1 | NR | 30.7 | 27.4 | 51.6 |
| Greenup et al. 2017 [38] | 1 | NR | NR | NR | NR | 99 | NR | NR | NR | NR | NR | NR | NR | NR | NR | 42 | 26 | NR | 70 |

| | | | | | | | | | | | | | | | | | | | |
|---|---|---|---|---|---|---|---|---|---|---|---|---|---|---|---|---|---|---|---|
| Hyun et al. 2022 [49] | NR | NR | NR | NR | NR | NR | NR | NR | NR | NR | NR | NR | NR | NR | NR | NR | NR | NR | NR |
| Ma et al. 2017 [39] | 14.4 | 85.6 | 75 | NR | 92.3 | NR | NR | NR | NR | NR | NR | NR | NR | NR | NR | 42.3 | 38.5 | 24 | 63.5 |
| Rowan et al. 2018 [61] | 0 | NR | NR | 5.3 | NR | 100 | NR | NR | NR | NR | NR | NR | NR | NR | NR | 42.1 | 15.8 | NR | 68.4 |
| Eberl et al. 2019 [3] | 14.6 | 85.4 | 66.7 | 43.8 | NR | NR | NR | NR | NR | NR | NR | NR | NR | 16.7 | 16.7 | NR | 47.9 | NR | 62.5 |
| Kubesch et al. 2019 [22] | 3.8 | NR | NR | 34.4 | NR | 96.2 | NR | NR | NR | NR | NR | NR | NR | NR | NR | NR | 35.8 | NR | NR |
| Liefferinckx et al. 2019 [40] | 0.6 | NR | NR | 69.7 | NR | 99.4 | NR | NR | NR | NR | NR | NR | 10.5 | 5.4 | NR | NR | 44.7 | 40.8 | 59.2 |
| Pugliese et al. 2019 [4] | 4.3 | NR | NR | 7.1 | NR | 95.7 | 88.6 | 54.3 | NR | NR | NR | 12.9 | NR | 7.1 | 8.6 | NR | 32.9 | NR | NR |
| Harris et al. 2020 [31] | 2.4 | 82.1 | 82.1 | 42.9 | 11.9 | 3.6 | NR | NR | NR | NR | NR | NR | 16.7 | 22.6 | 6 | NR | 7.1 | 65 | 51 |
| Thomann et al. 2020 [23] | NR | NR | NR | NR | NR | NR | NR | NR | NR | NR | NR | NR | NR | NR | NR | NR | NR | NR | NR |
| Bermejo et al. 2022 [20] | NR | 73.5 | 81.8 | 39.6 | NR | NR | NR | 20.7 | 39.6 | NR | 92.5 | NR | NR | NR | NR | 24.5 | NR | 41.5 | 73.6 |
| Garg et al. 2022 [28] | NR | 33.3 | 33.3 | 8.6 | 24.7 | NR | 47.4 | NR | NR | NR | NR | NR | NR | NR | NR | 14.1 | 49.3 | NR | 72.4 |

| | | | | | | | | | | | | | | | | | | | |
|---|---|---|---|---|---|---|---|---|---|---|---|---|---|---|---|---|---|---|---|
| Hanzel et al. 2021 [42] | 42.5 | NR | NR | 22 | NR | 58.5 | NR | NR | NR | NR | NR | NR | 7.3 | 2.4 | NR | 9.7 | 19.5 | 14.6 | 63.4 |
| Straatmijer et al. 2021 [34] | 0.88 | NR | NR | 42.9 | NR | 99.2 | NR | 19.4 | 6 | NR | NR | NR | NR | NR | NR | NR | 18.7 | 13.1 | NR |
| Tursi et al. 2021 [41] | 24.2 | NR | NR | 24.2 | NR | 75.8 | 76.3 | NR | 12.4 | NR | 50 | NR | NR | NR | NR | NR | 91.2 | NR | NR |
| Yao et al. 2021 [43] | 11.1 | 68.8 | 22.2 | 5 | 88.9 | NR | 61.1 | NR | NR | NR | NR | NR | NR | NR | NR | NR | NR | 16.7 | 33.3 |
| Yao et al. 2021 [45] | NR | NR | NR | NR | NR | NR | NR | NR | NR | NR | NR | NR | NR | NR | NR | NR | NR | NR | NR |
| Yao et al. 2021 [44] | NR | NR | NR | NR | NR | NR | NR | NR | NR | NR | NR | NR | NR | NR | NR | NR | NR | NR | NR |
| Chaparro et al. 2021 [51] | NR | 98 | NR | 82 | NR | NR | NR | NR | NR | NR | NR | NR | NR | NR | NR | NR | NR | NR | NR |
| Amiot et al. 2020 [52] | 1 | NR | NR | 85.4 | NR | 99 | NR | NR | 24.3 | NR | NR | NR | NR | NR | NR | NR | NR | NR | NR |
| Chiappetta et al. 2021 [55] | 3 | NR | NR | 2 | NR | 29 | NR | NR | NR | NR | NR | NR | 22 | NR | NR | NR | 54 | NR | NR |
| Fumery et al. 2021 [53] | 1 | NR | NR | 85.4 | NR | 99 | NR | NR | 24.3 | NR | NR | NR | NR | NR | NR | NR | NR | NR | NR |
| Dalal et al. 2021 [56] | 8.3 | NR | NR | NR | NR | 91.7 | NR | NR | NR | NR | NR | NR | NR | NR | NR | NR | NR | NR | NR |

*IMS:* immunosuppressant, *TNF:* tumor necrosis factor, *NR:* not reported, *CS:* corticosteroid

Supplementary Table S2: Cochrane Handbook for Systematic Reviews and the Preferred Reporting Items for Systematic Reviews and Meta-Analyses (PRISMA) guidelines

| Section/topic | # | Checklist item | Reported on page # |
|---|---|---|---|
| TITLE | | | |
| Title | 1 | Identify the report as a systematic review, meta-analysis, or both. | 1 |
| ABSTRACT | | | |
| Structured summary | 2 | Provide a structured summary including, as applicable: background; objectives; data sources; study eligibility criteria, participants, and interventions; study appraisal and synthesis methods; results; limitations; conclusions and implications of key findings; systematic review registration number. | 2 |
| INTRODUCTION | | | |
| Rationale | 3 | Describe the rationale for the review in the context of what is already known. | 1 |
| Objectives | 4 | Provide an explicit statement of questions being addressed with reference to participants, interventions, comparisons, outcomes, and study design (PICOS). | 2 |
| METHODS | | | |
| Protocol and registration | 5 | Indicate if a review protocol exists, if and where it can be accessed (e.g., Web address), and, if available, | 2 |

| | | | |
|---|---|---|---|
| | | provide registration information including registration number. | |
| Eligibility criteria | 6 | Specify study characteristics (e.g., PICOS, length of follow-up) and report characteristics (e.g., years considered, language, publication status) used as criteria for eligibility, giving rationale. | 2 |
| Information sources | 7 | Describe all information sources (e.g., databases with dates of coverage, contact with study authors to identify additional studies) in the search and date last searched. | 2 |
| Search | 8 | Present full electronic search strategy for at least one database, including any limits used, such that it could be repeated. | 2 |
| Study selection | 9 | State the process for selecting studies (i.e., screening, eligibility, included in systematic review, and, if applicable, included in the meta-analysis). | 2 |
| Data collection process | 10 | Describe method of data extraction from reports (e.g., piloted forms, independently, in duplicate) and any processes for obtaining and confirming data from investigators. | 2 |
| Data items | 11 | List and define all variables for which data were sought (e.g., PICOS, funding sources) and any assumptions and simplifications made. | 2 |
| Risk of bias in individual studies | 12 | Describe methods used for assessing risk of bias of individual studies (including specification of whether this was done at the study or outcome level), and how this information is to be used in any data synthesis. | 2 |

| | | | |
|---|---|---|---|
| Summary measures | 13 | State the principal summary measures (e.g., risk ratio, difference in means). | 2 |
| Synthesis of results | 14 | Describe the methods of handling data and combining results of studies, if done, including measures of consistency (e.g., $I^2$) for each meta-analysis. | 3 |

Supplementary Figure S1: Clinical response at the induction in ulcerative colitis

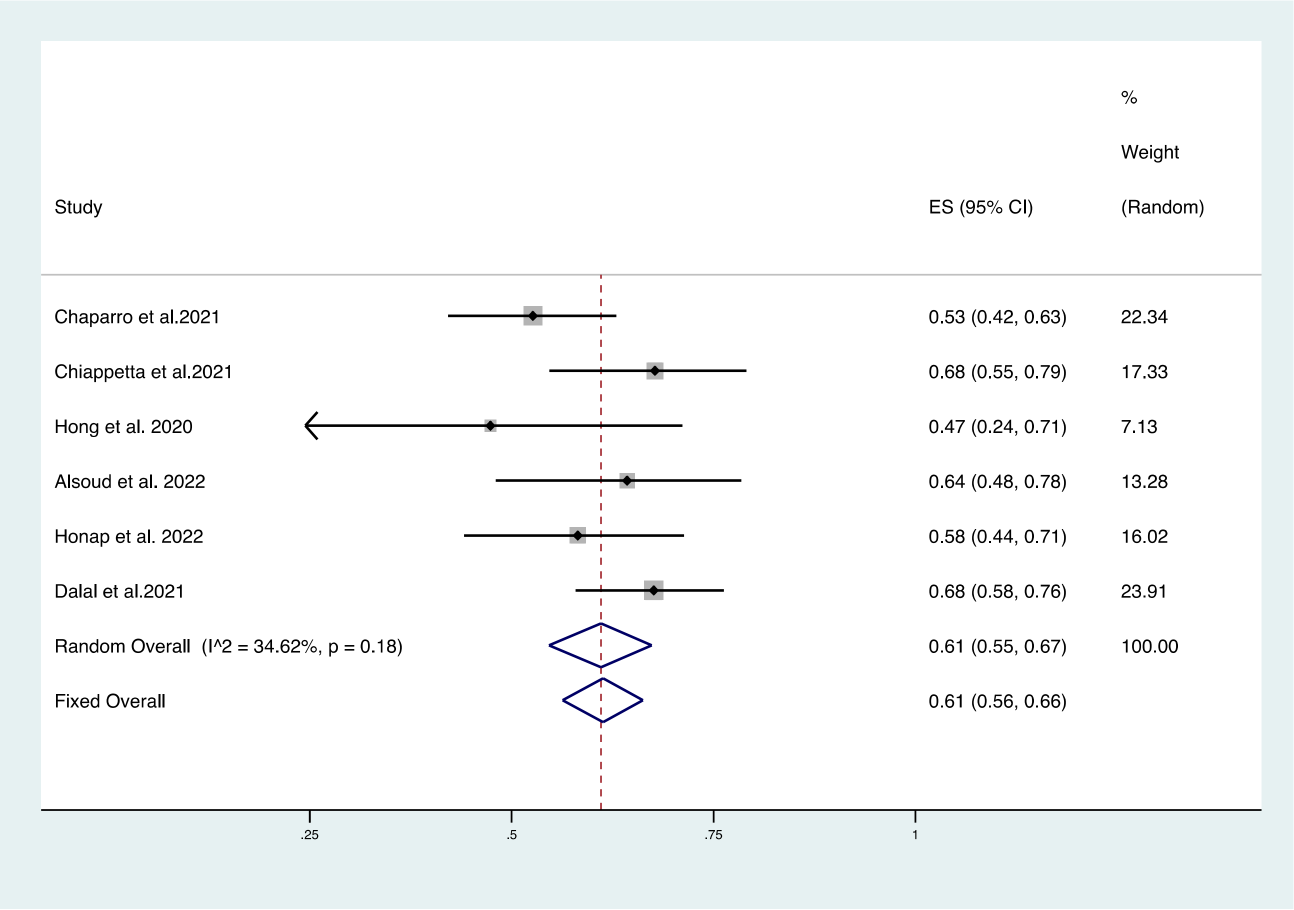

Supplementary Figure S2: Clinical response in Crohn's disease

A： 12-week (induction)

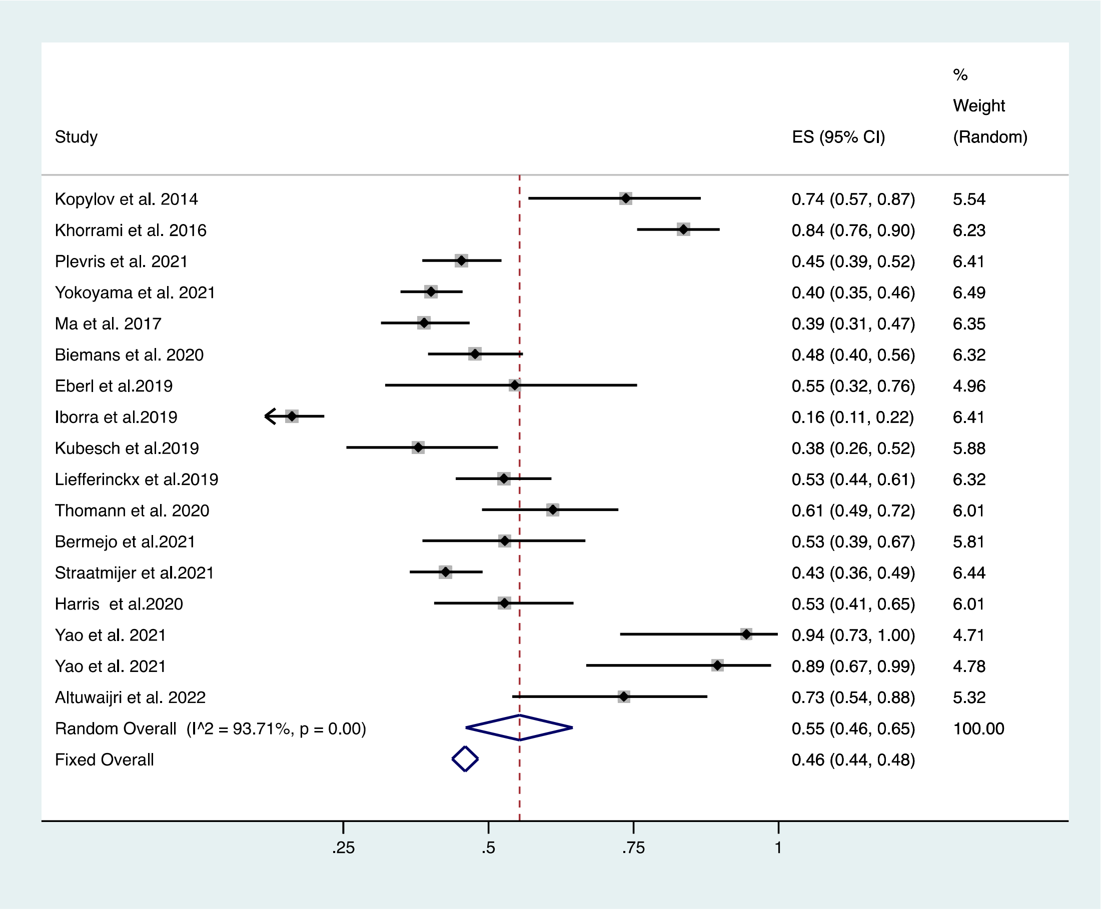

B：24-week (maintenance)

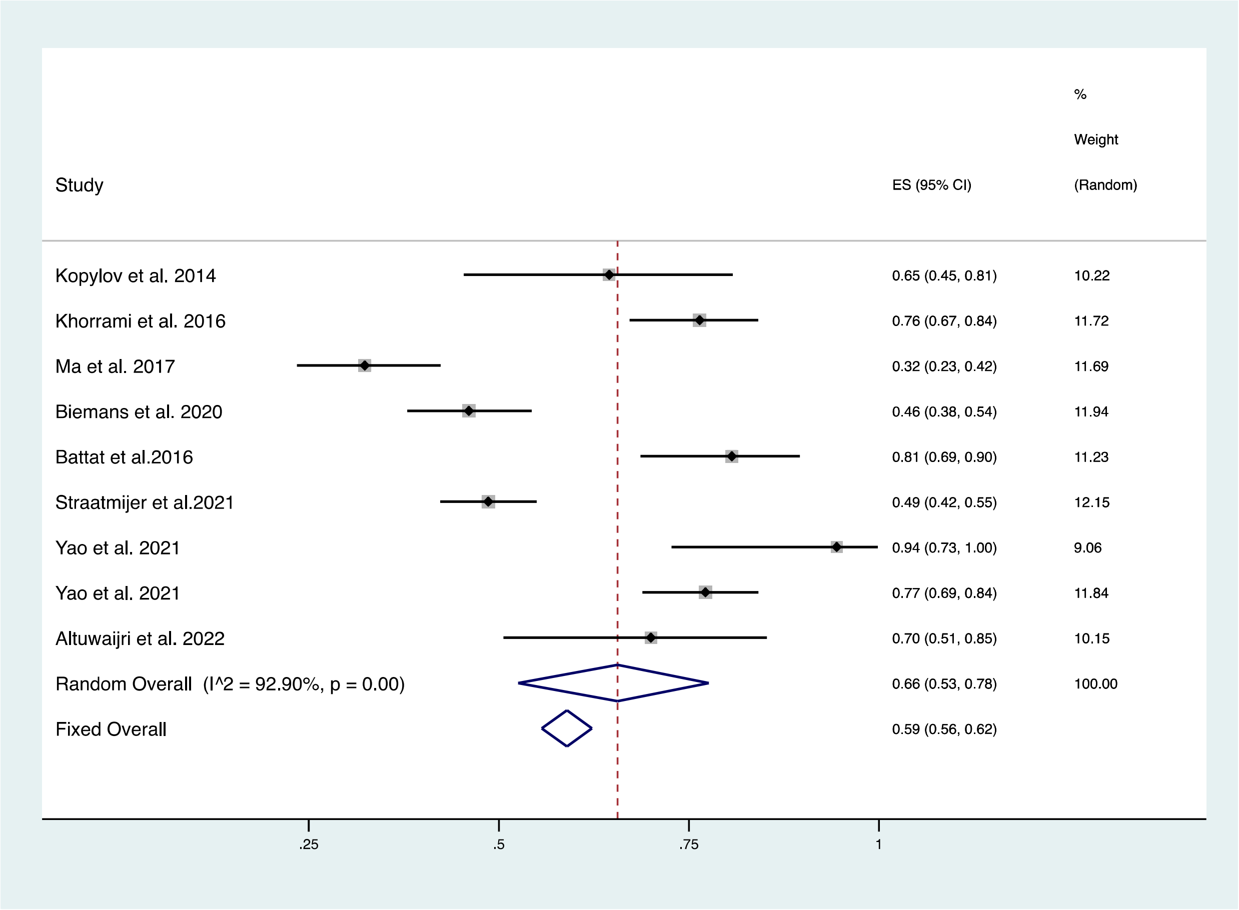

C： 1-year (maintenance)

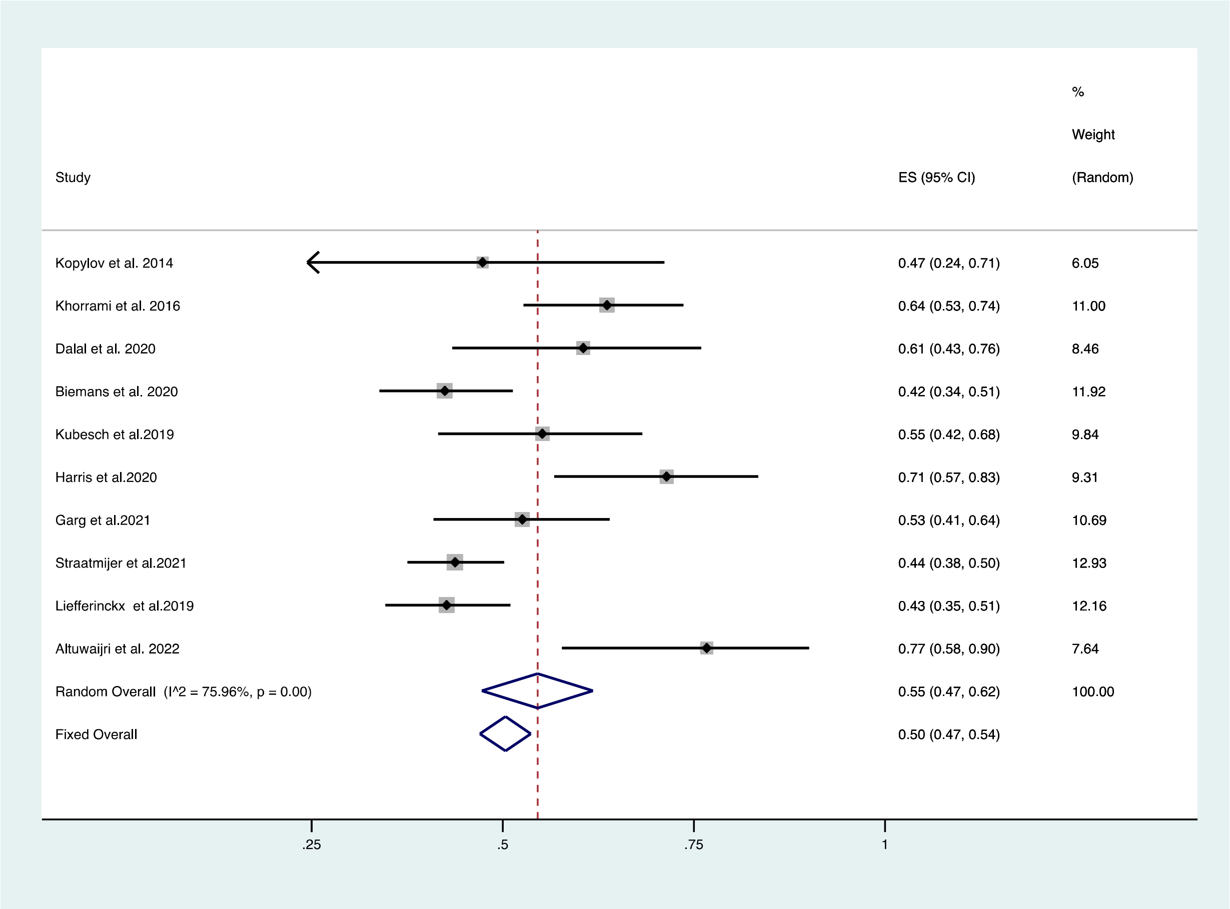

Supplementary Figure S3: CS-free remission in ulcerative colitis

A： week 12 (induction)

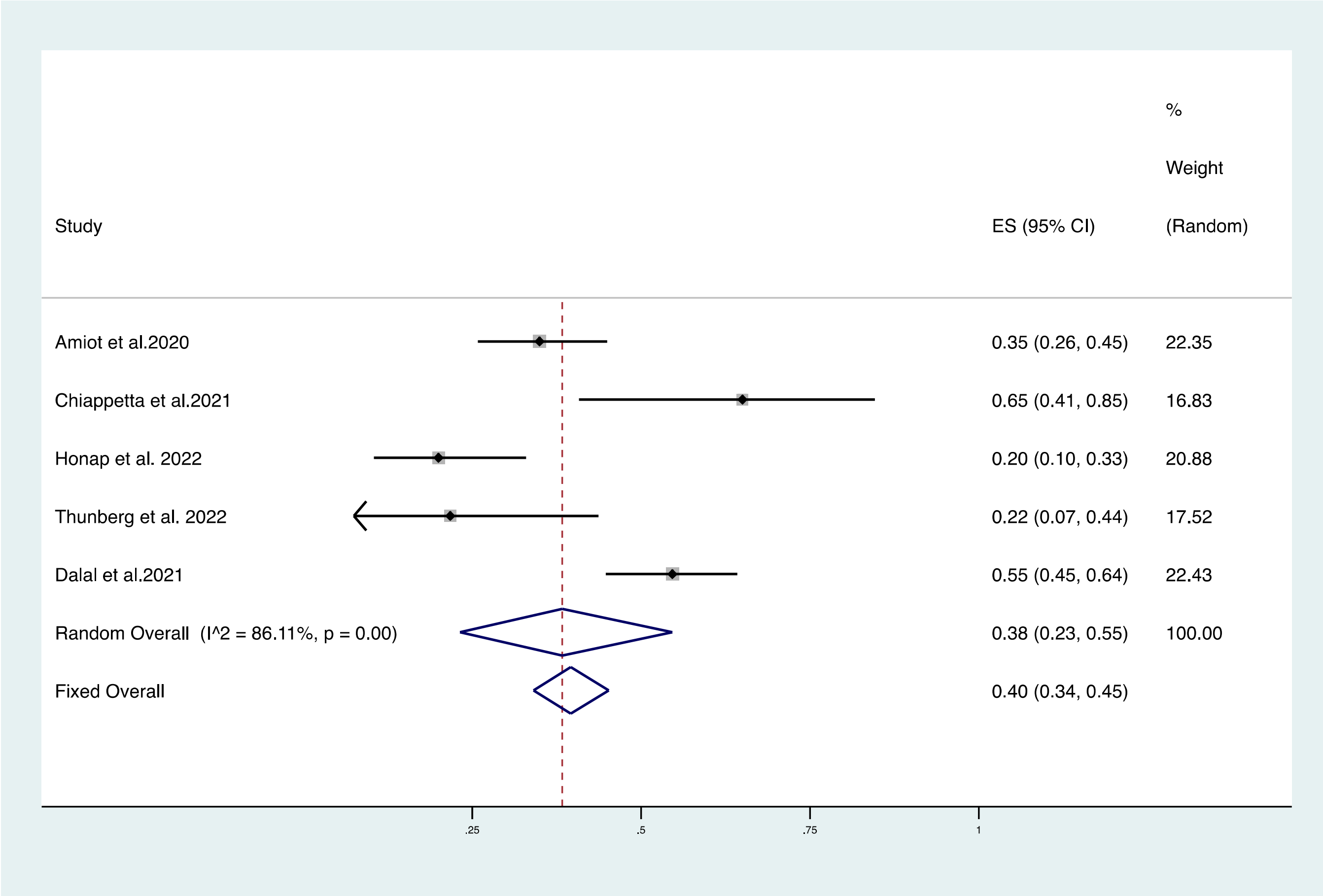

B： week 24 (maintenance)

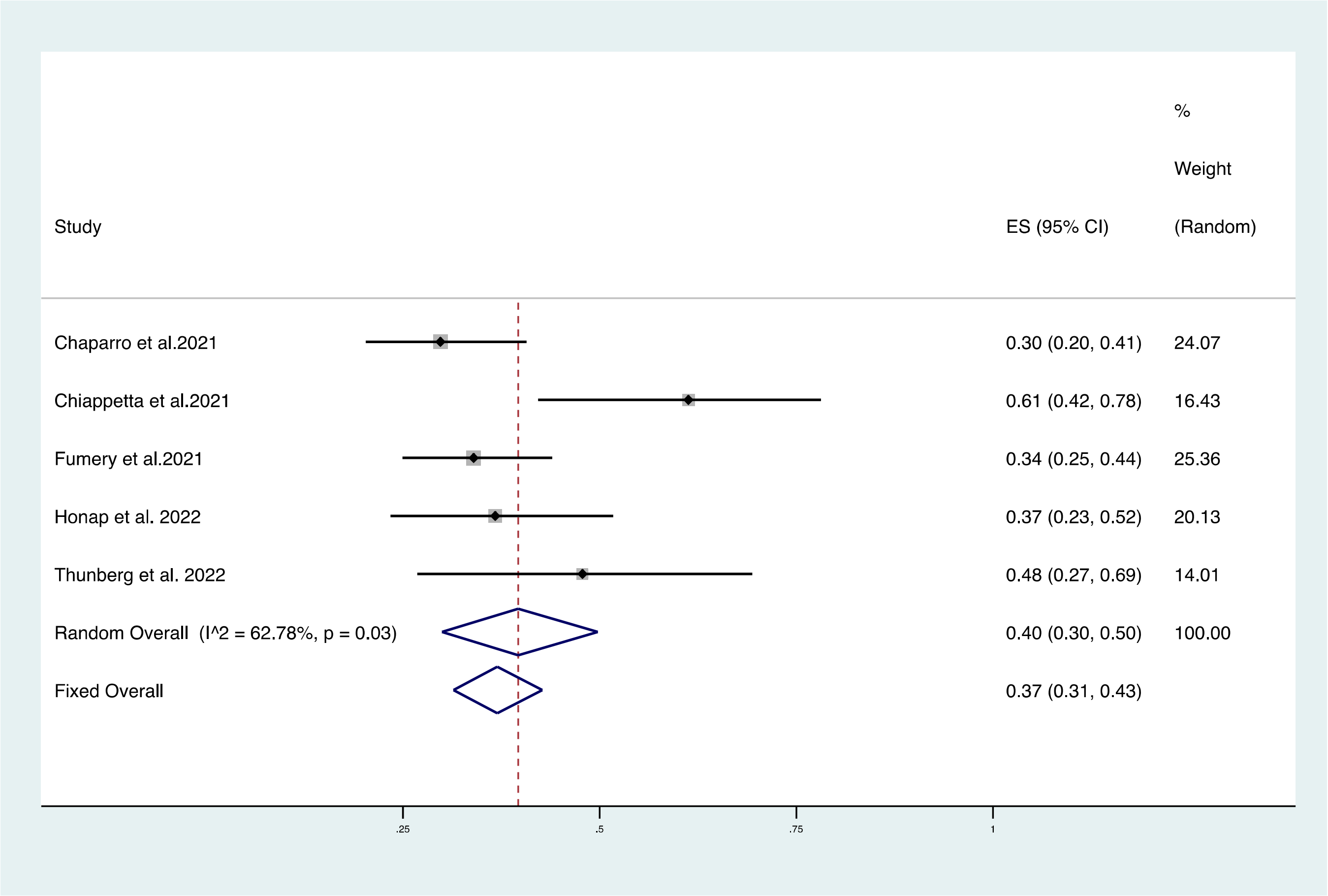

C： 1-year (maintenance)

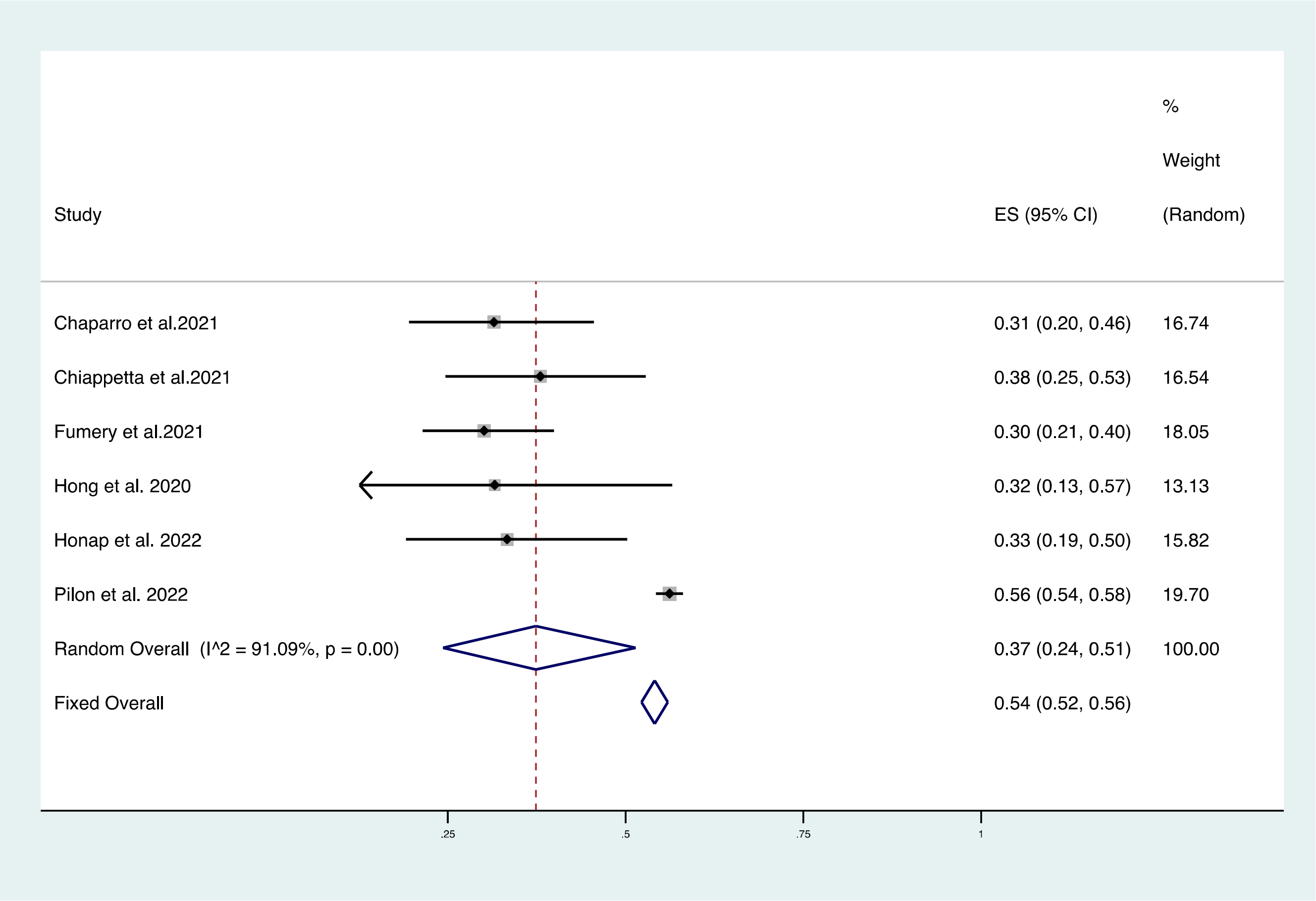

Supplementary Figure S4: CS-free remission in Crohn's disease

A： week 12 (induction)

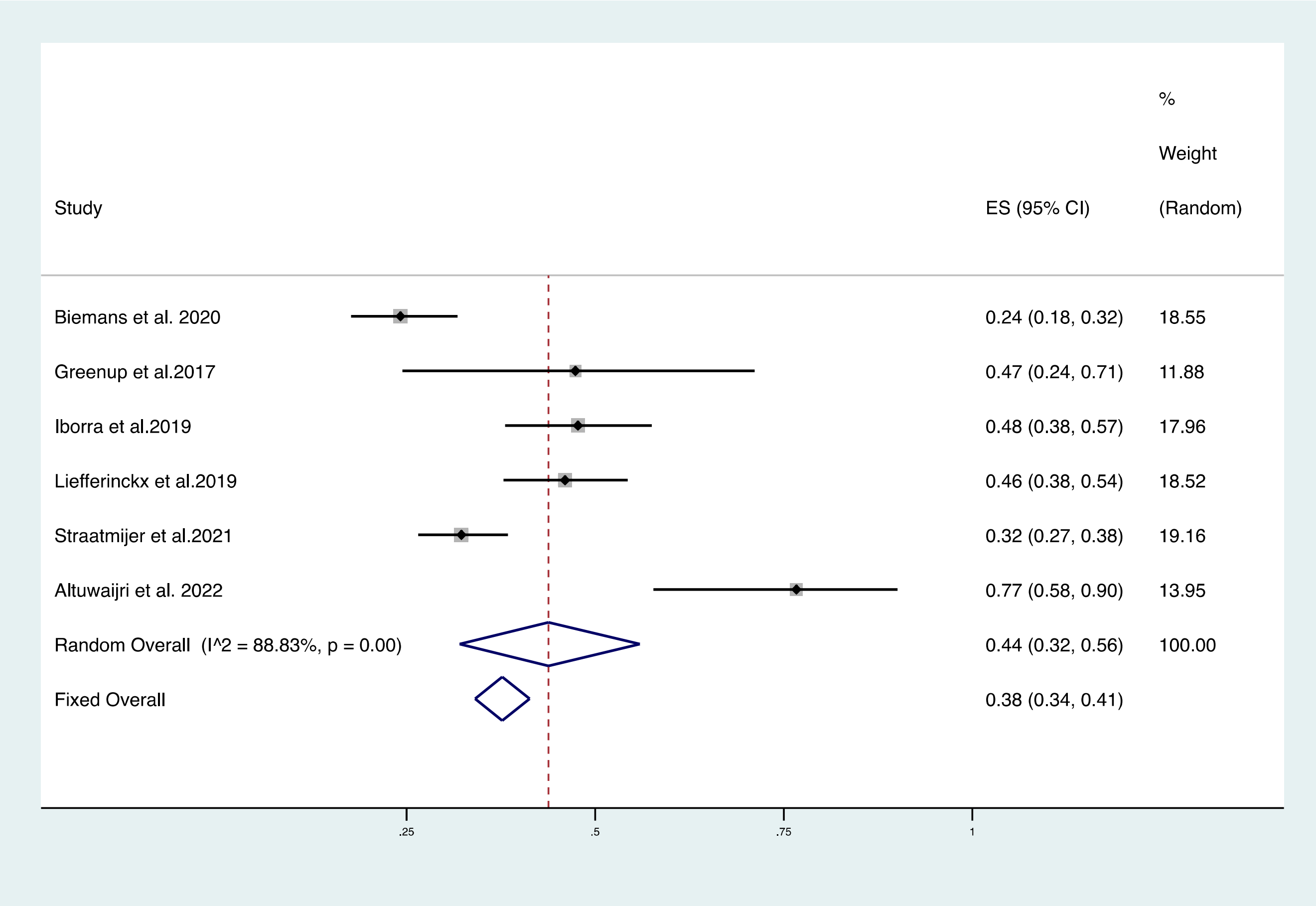

B： week 24 (maintenance)

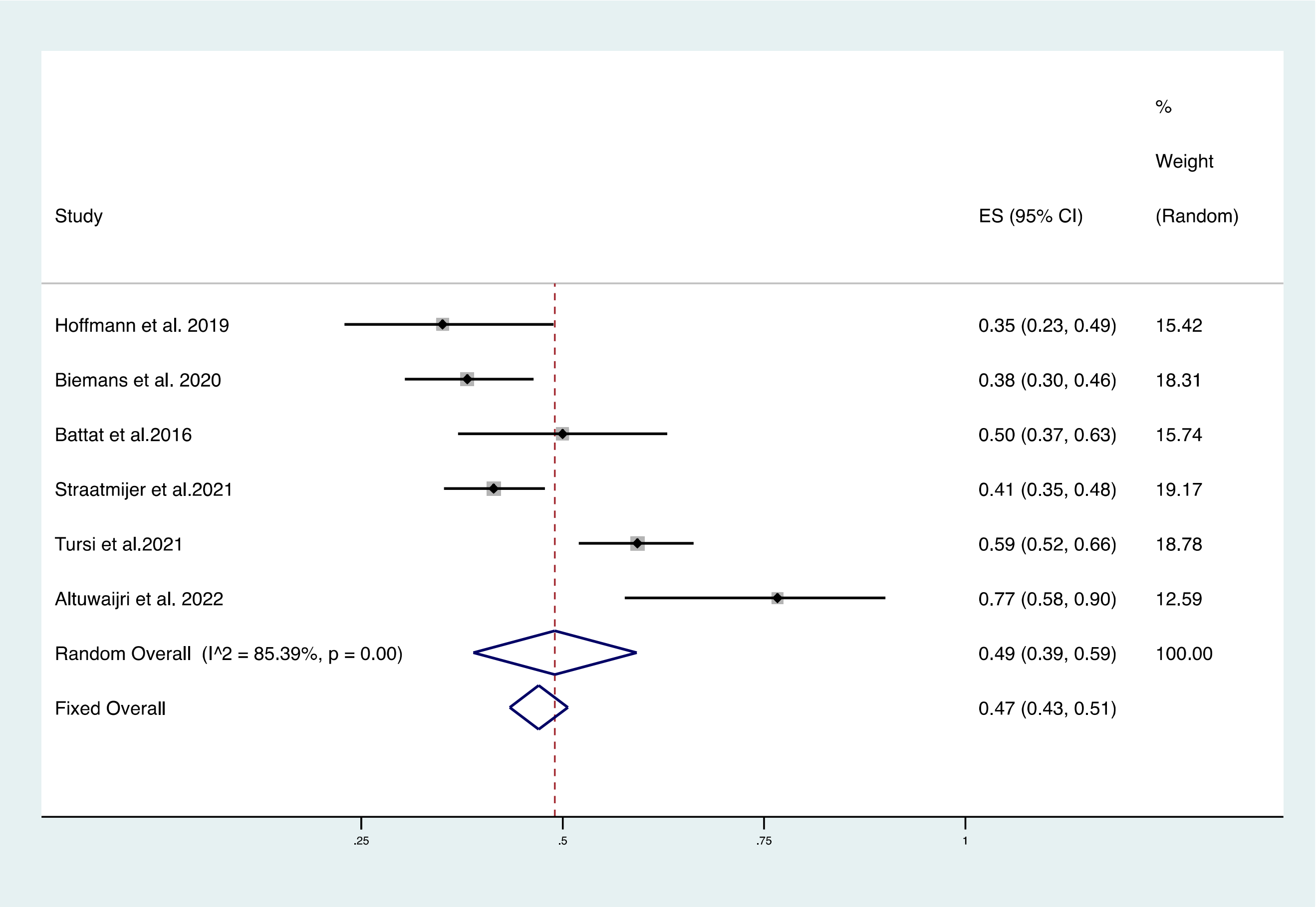

C: 1-year (maintenance)

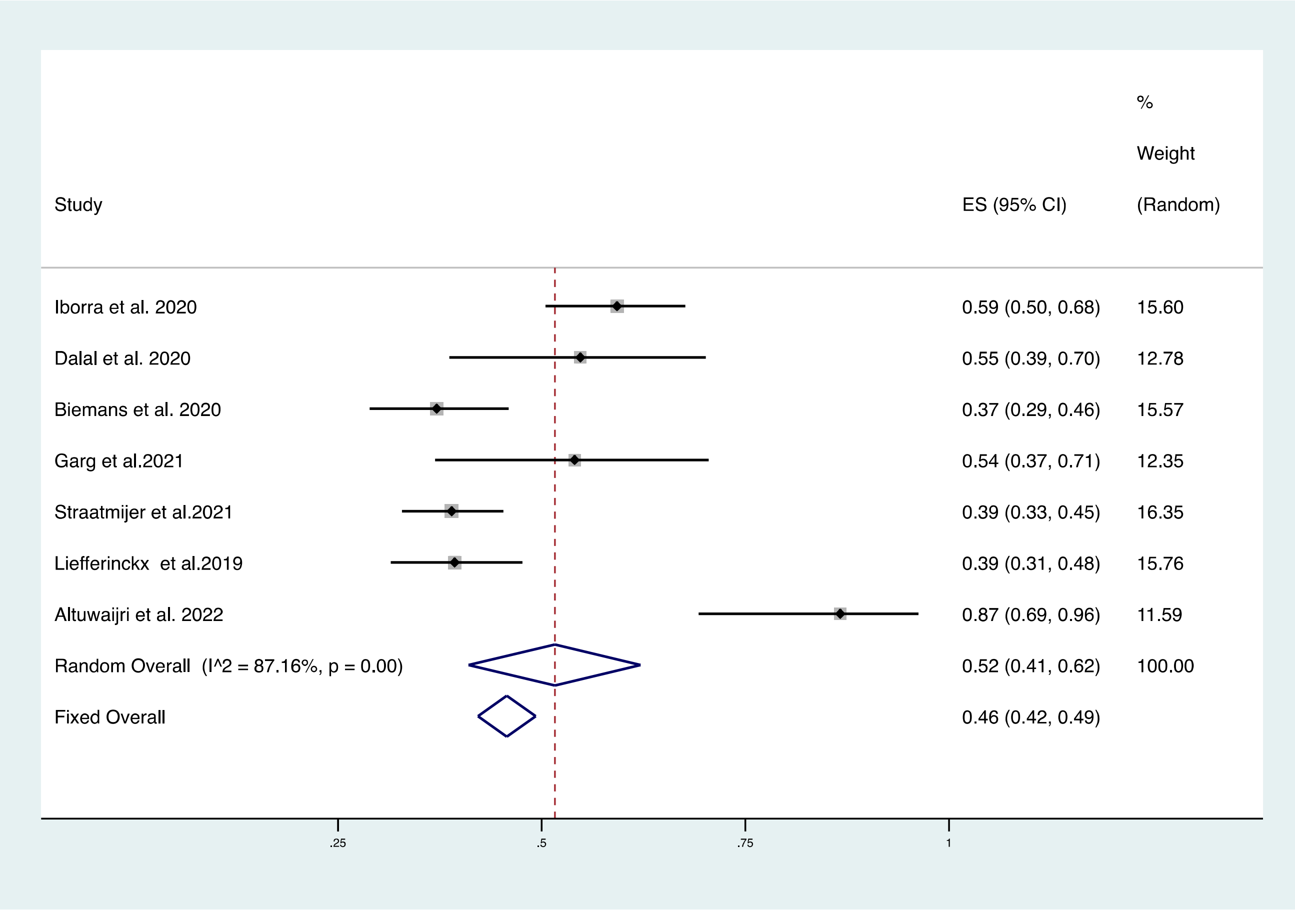

Supplementary Figure S5: Endoscopic response in the 1-year maintenance in Crohn's disease

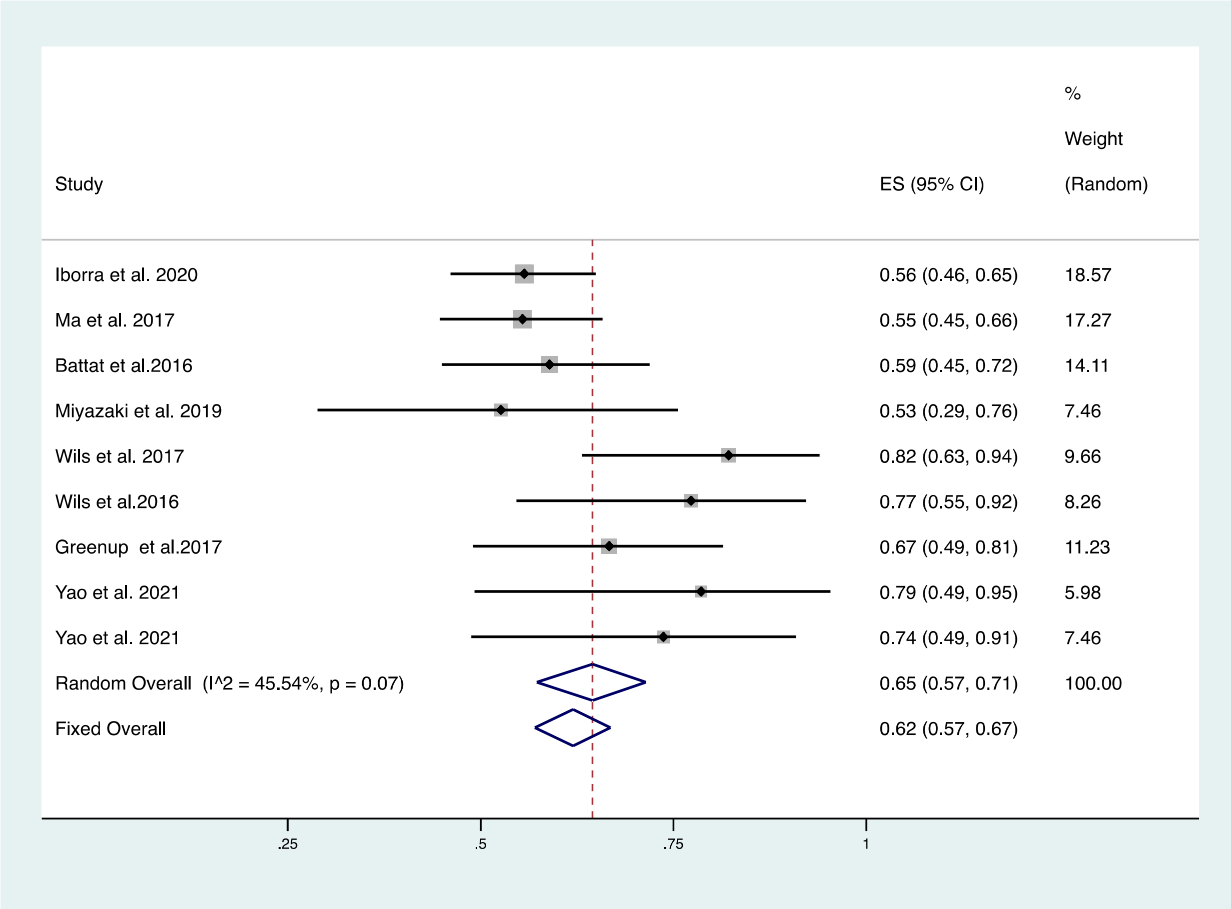

Supplementary Figure S6: Endoscopic remission in the 1-year maintenance in Crohn's disease

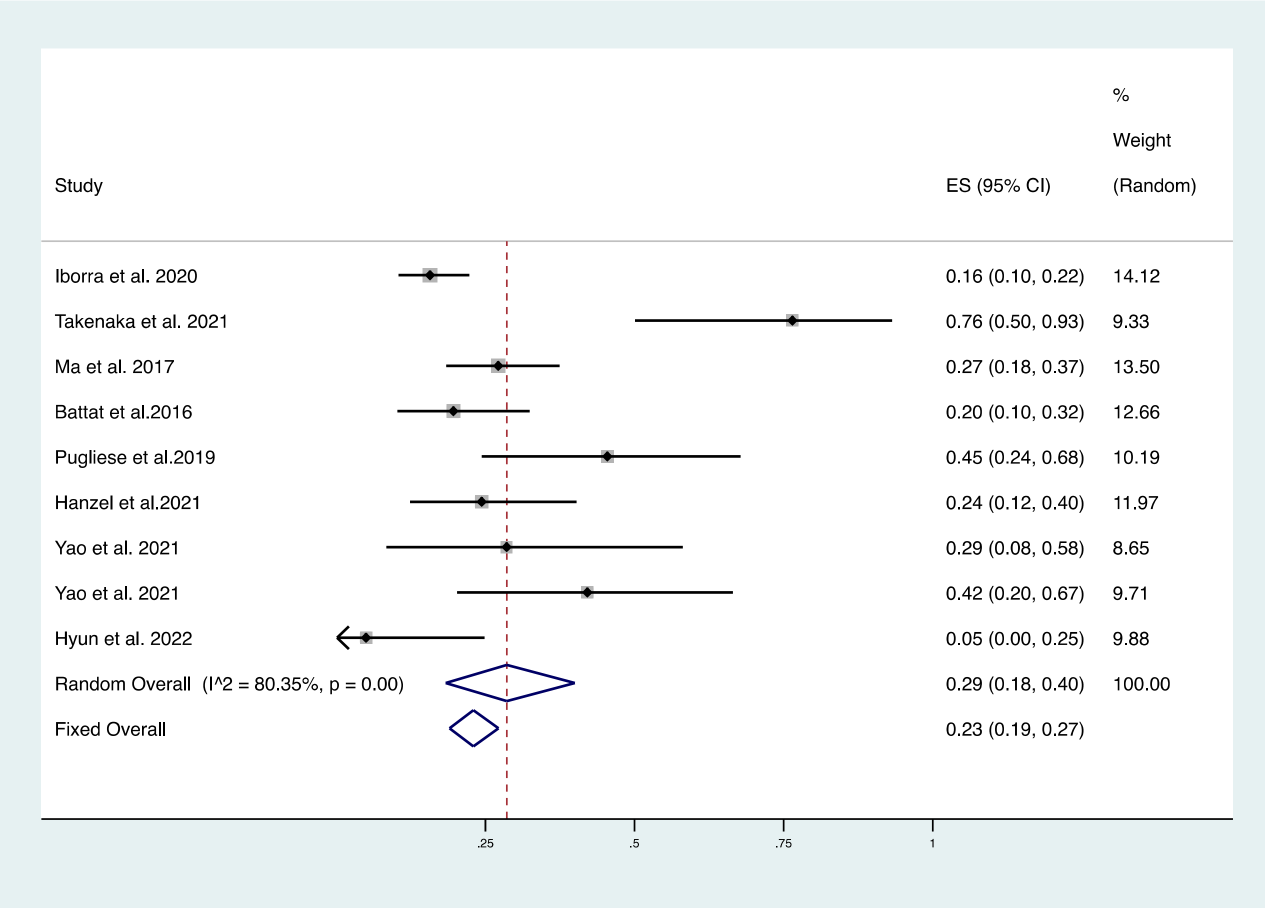

Supplementary Figure S7: Mucosal healing in the 1-year maintenance in Crohn's disease

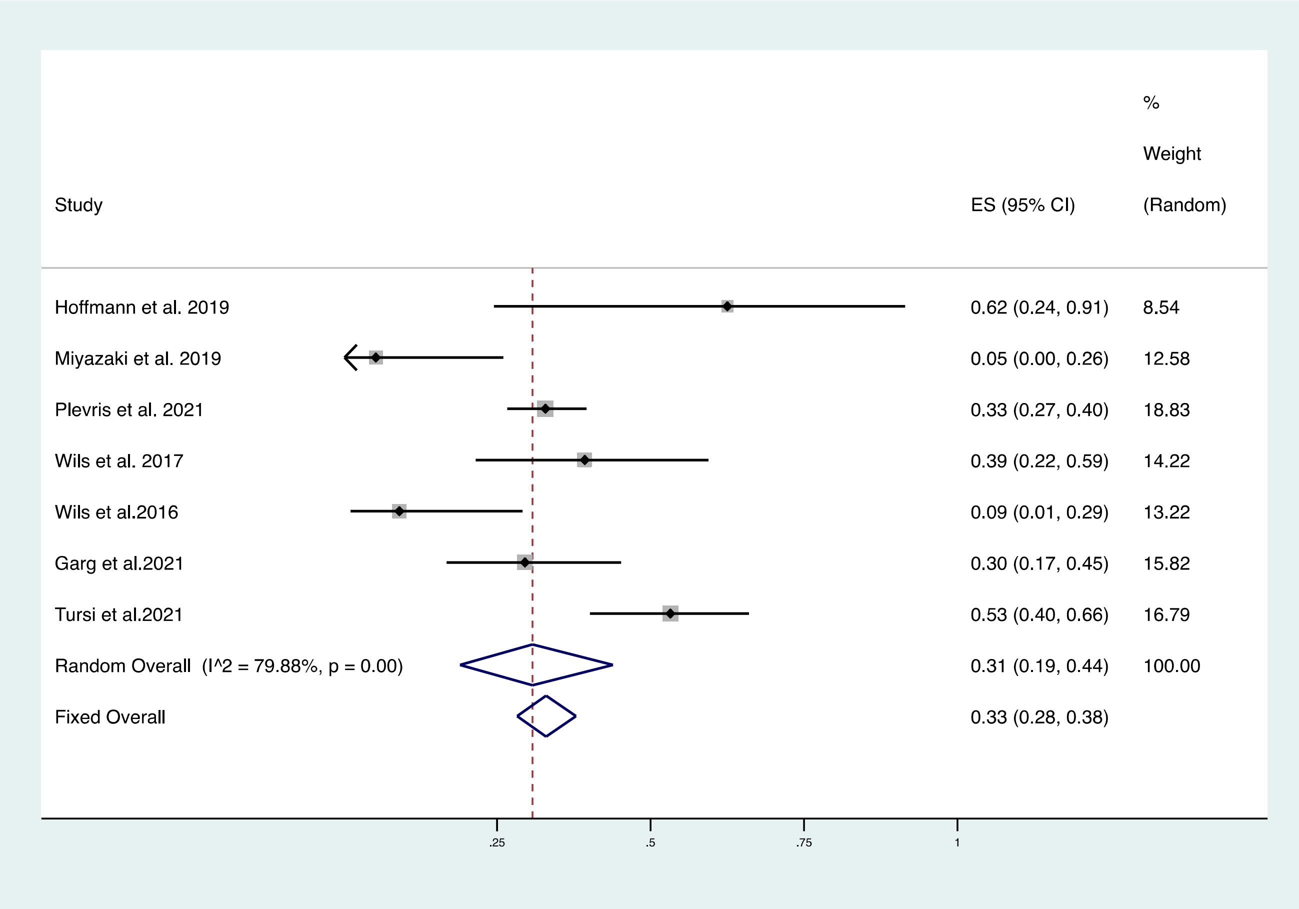

Supplementary Figure S8: Clinical response in Western countries in Crohn's disease

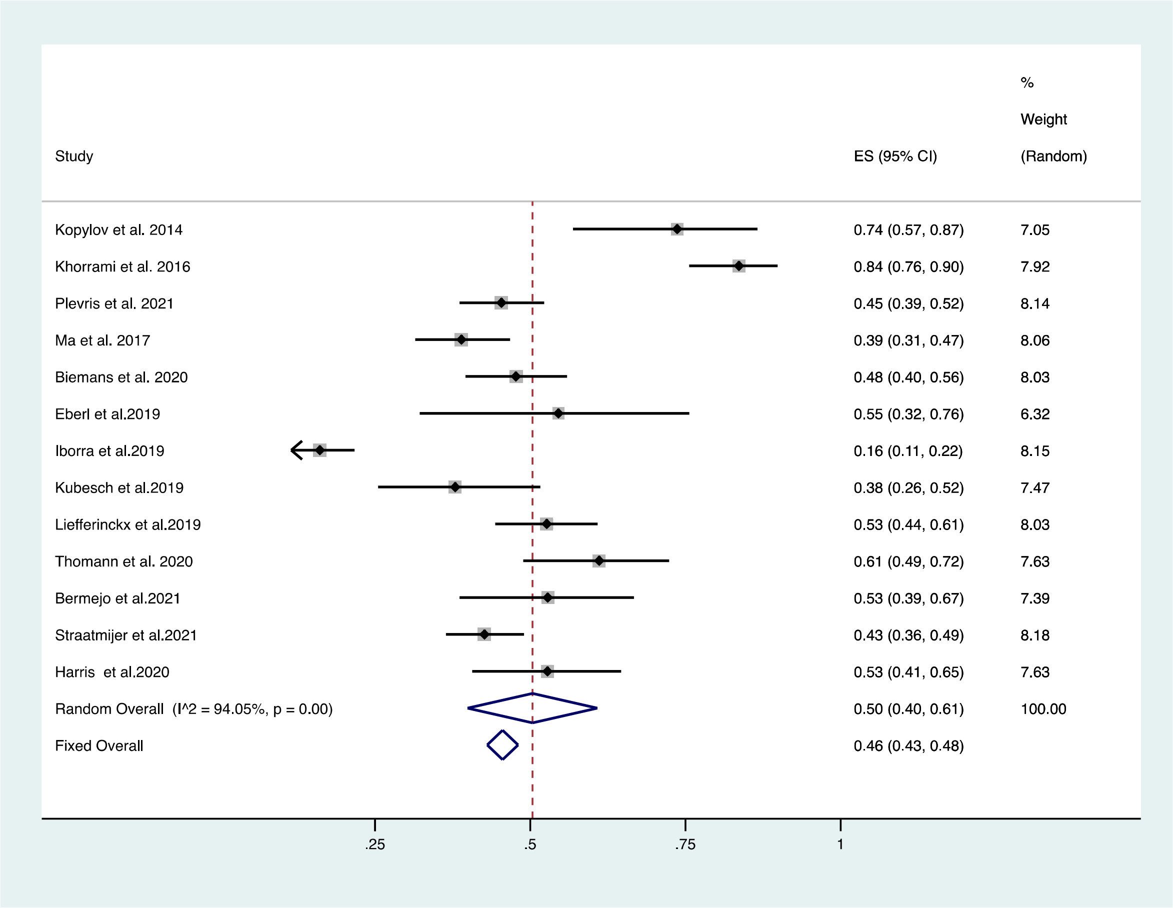

Supplementary Figure S9: Clinical response in Eastern countries in Crohn's disease

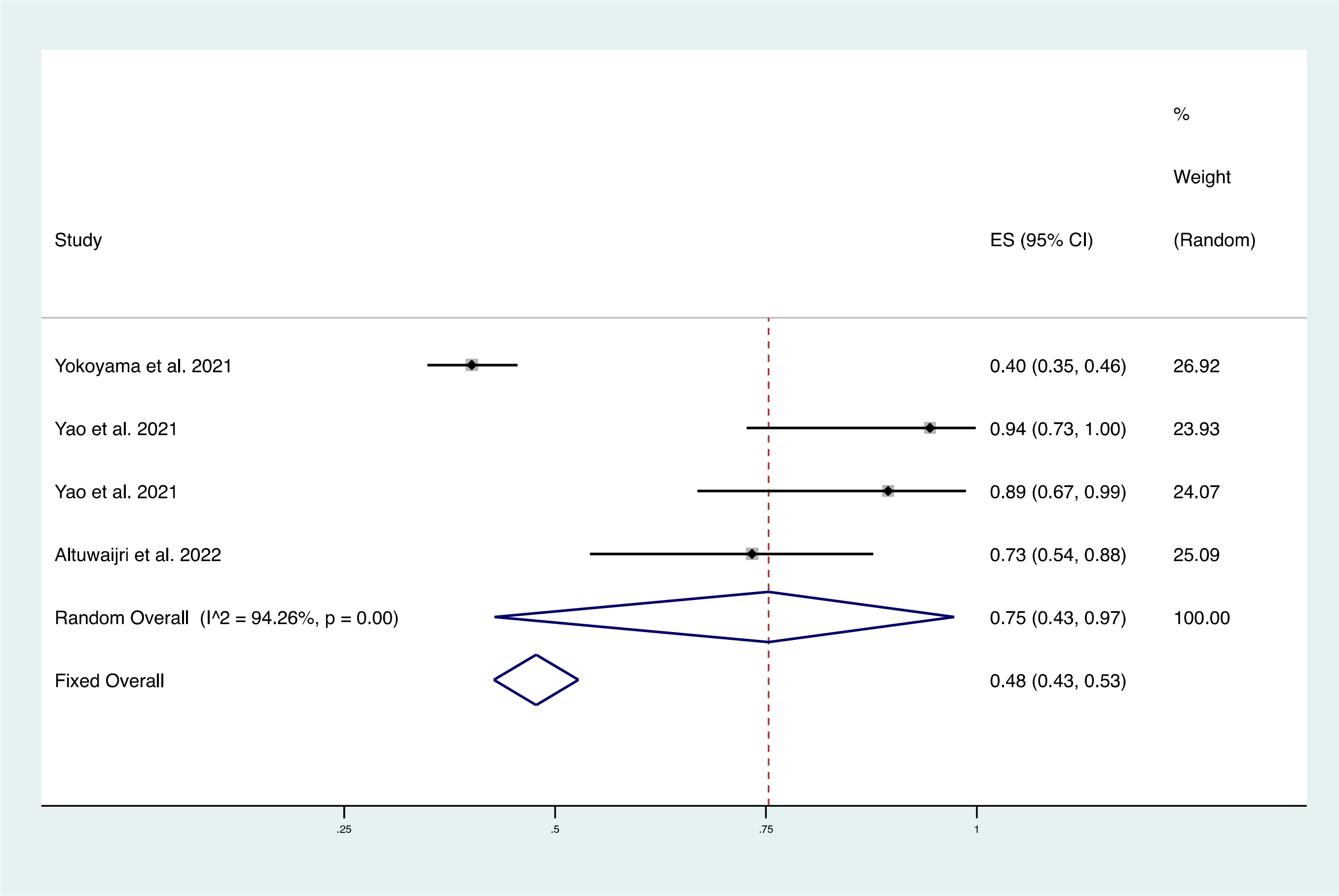

Supplementary Figure S10: Adverse events in ulcerative colitis

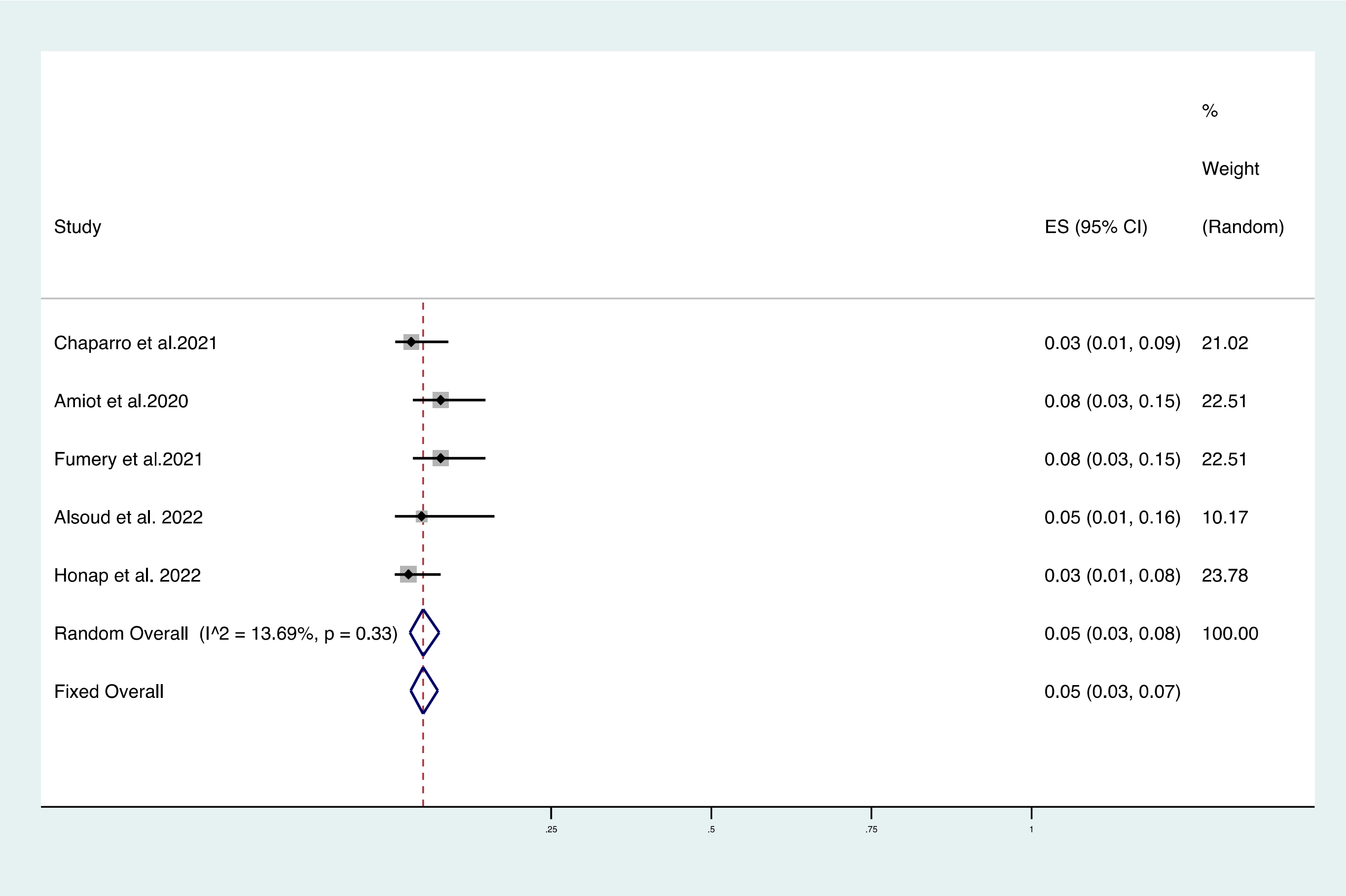

Supplementary Figure S11: Adverse events in Crohn’s disease

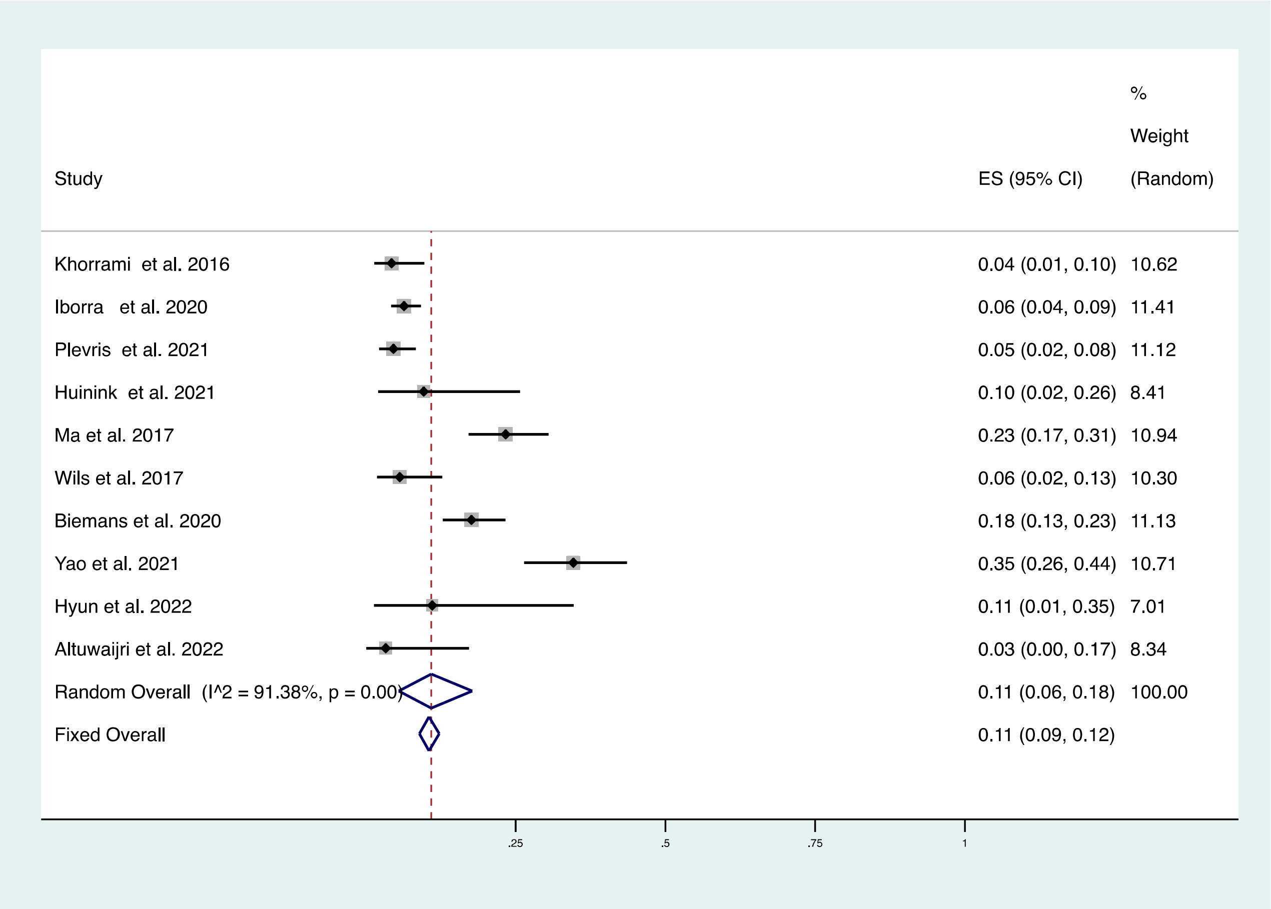

Supplementary Figure S12: Clinical remission in biological native patients

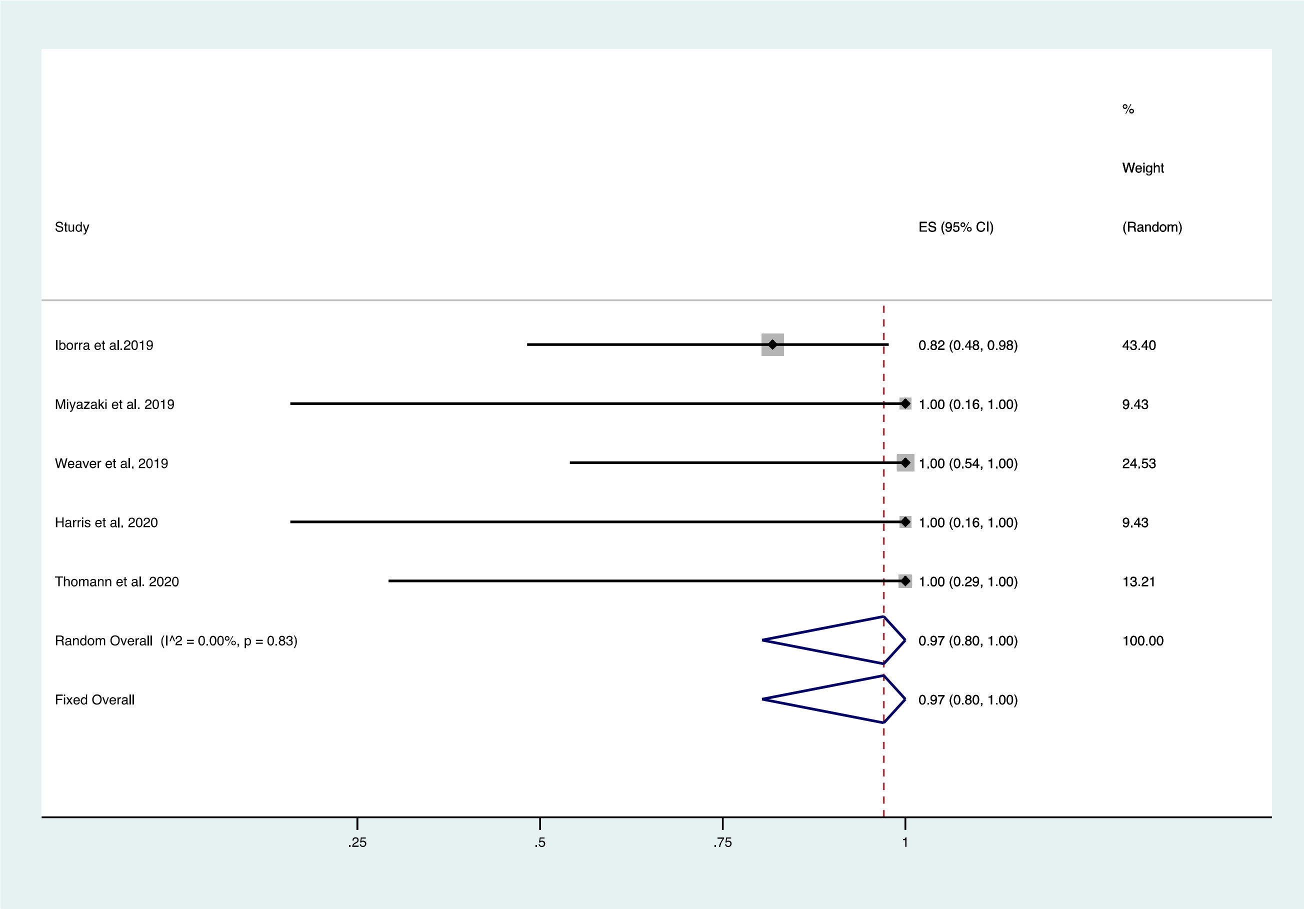

Supplementary Figure S13: Clinical remission in biological experienced patients

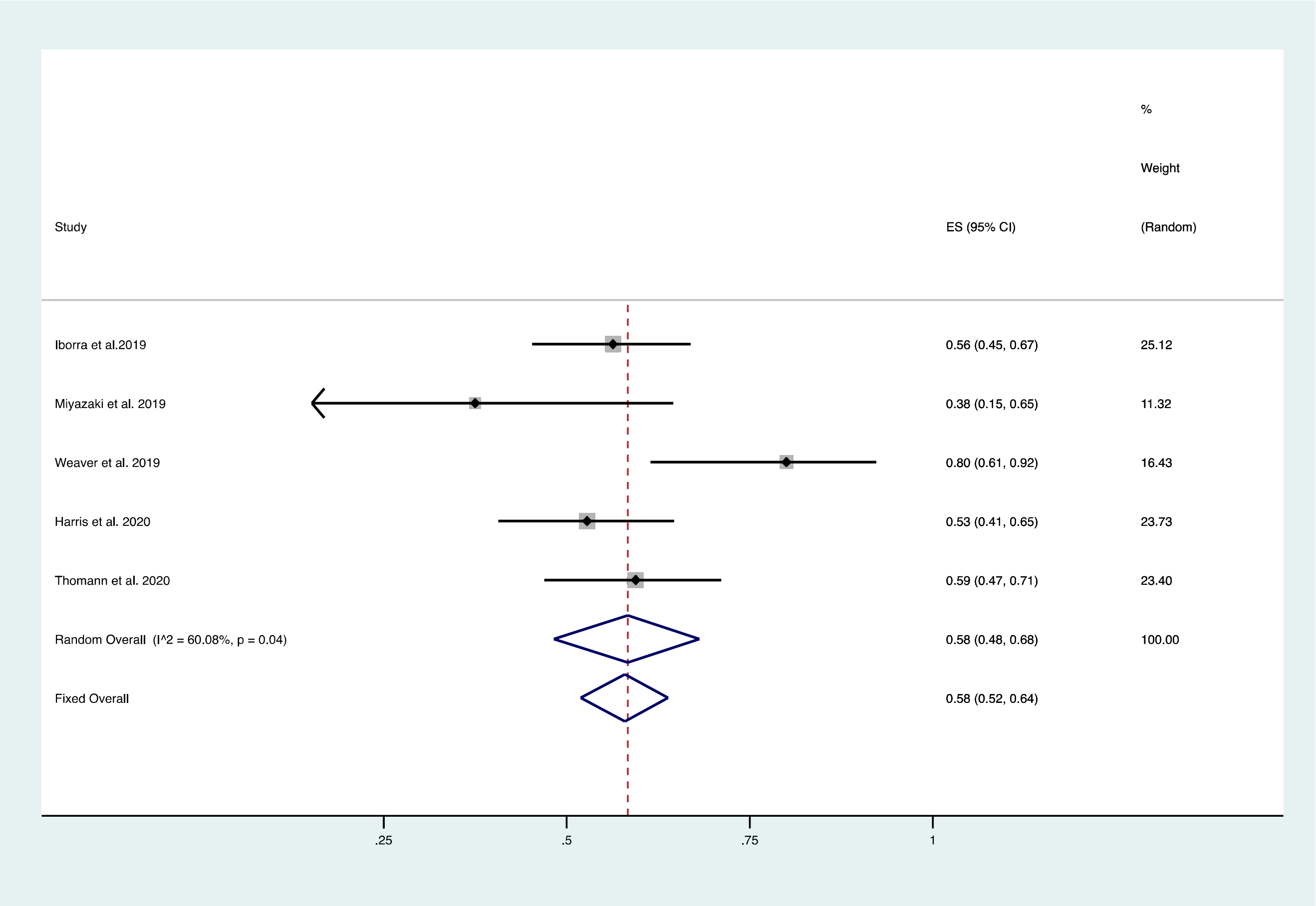

Supplementary Figure S14: Publication bias of clinical response at induction in Crohn’s disease

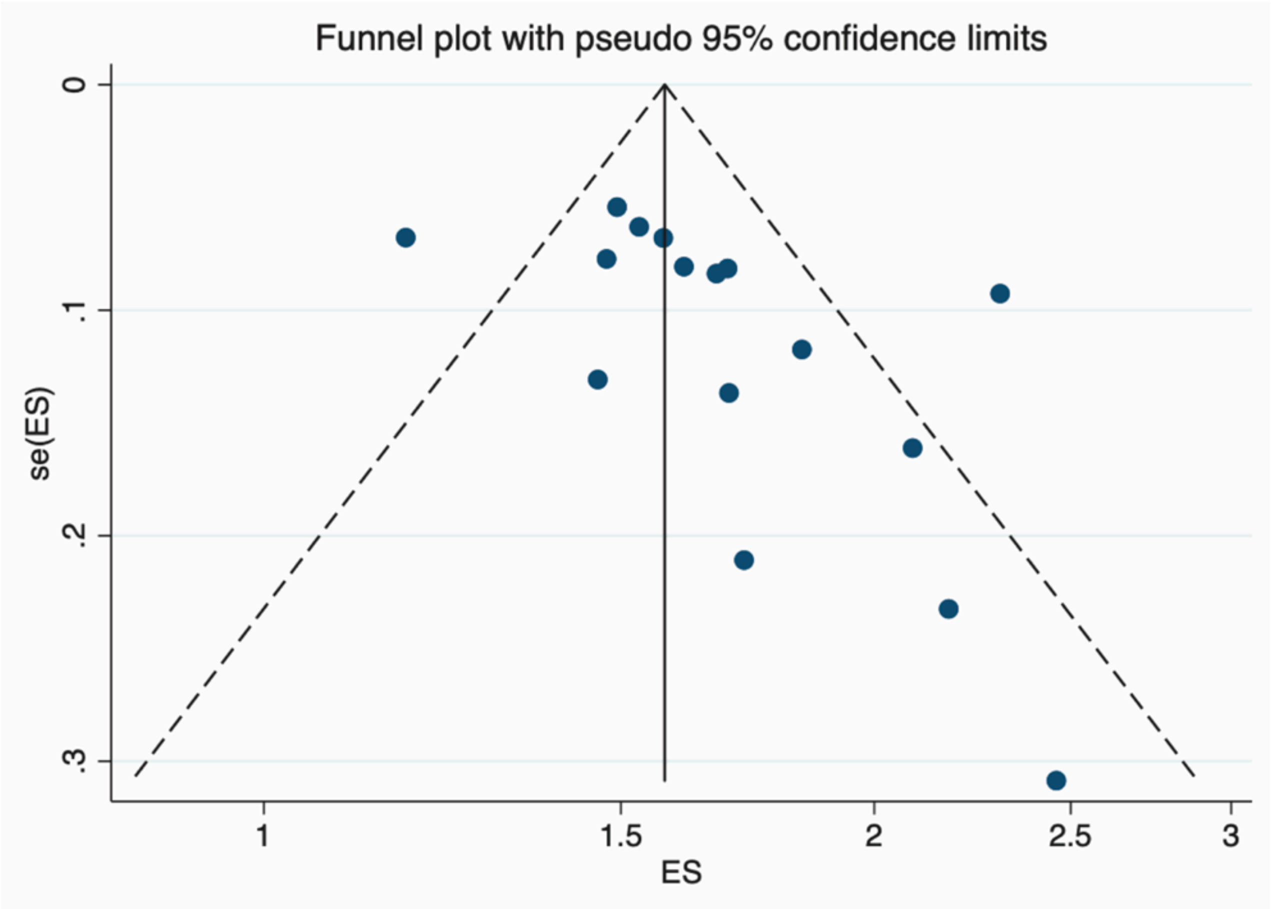

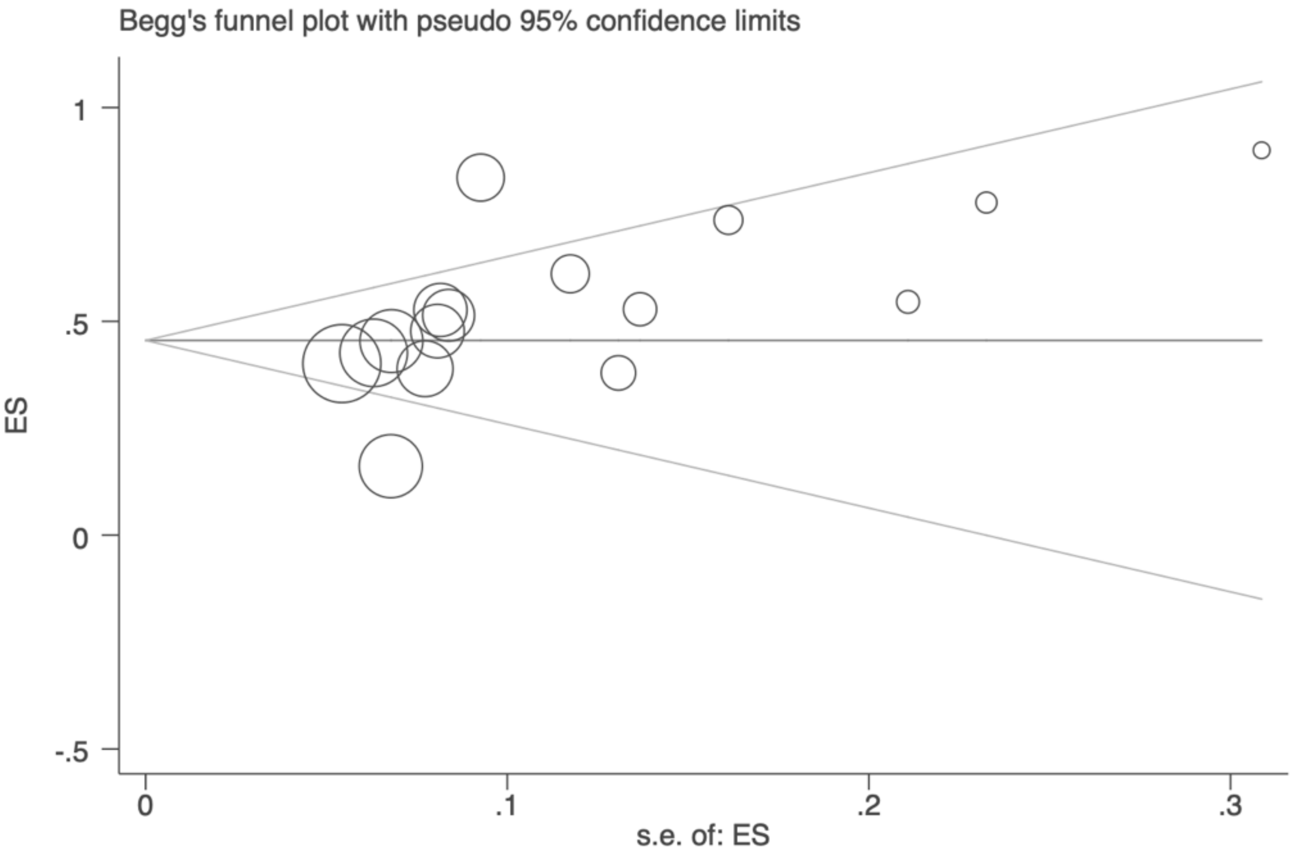
Begg's funnel plot with pseudo 95% confidence limits
1
.5
0
-.5
ES
0
.1
.2
.3
s.e. of: ES

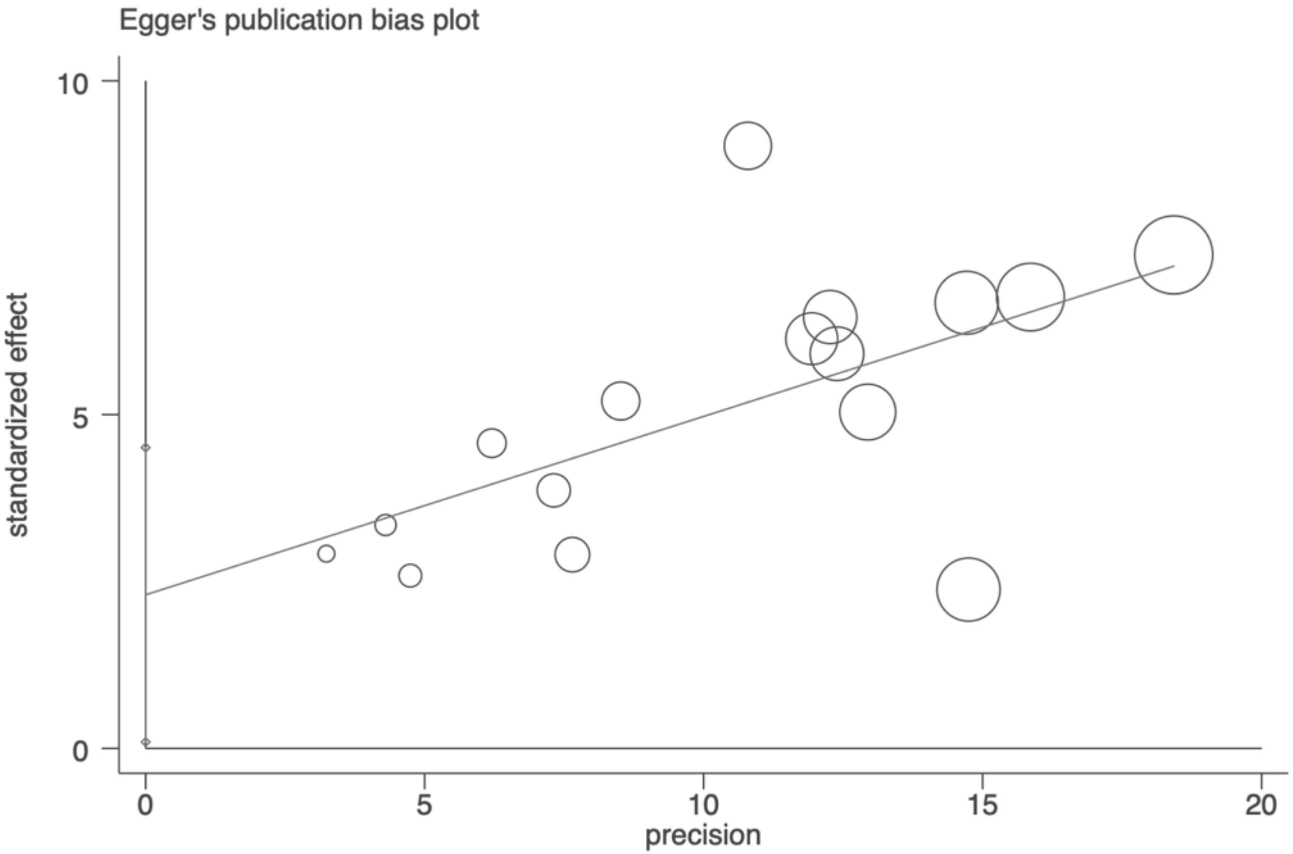
Egger's publication bias plot
standardized effect
precision
10
5
0
0
5
10
15
20

Supplementary Figure S15: Clinical response rates by geographic location at induction in Crohn's disease

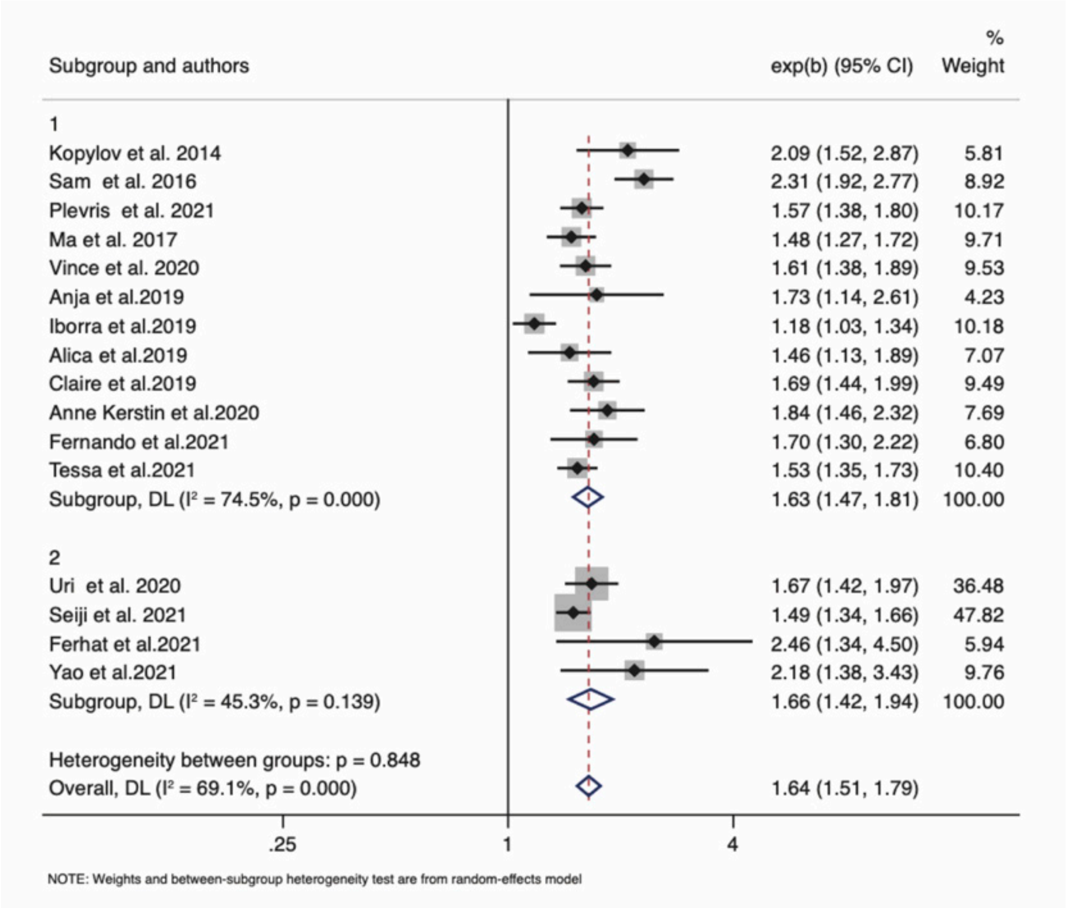

Supplementary Figure S16: Publication bias of clinical remission at induction in Crohn’s disease

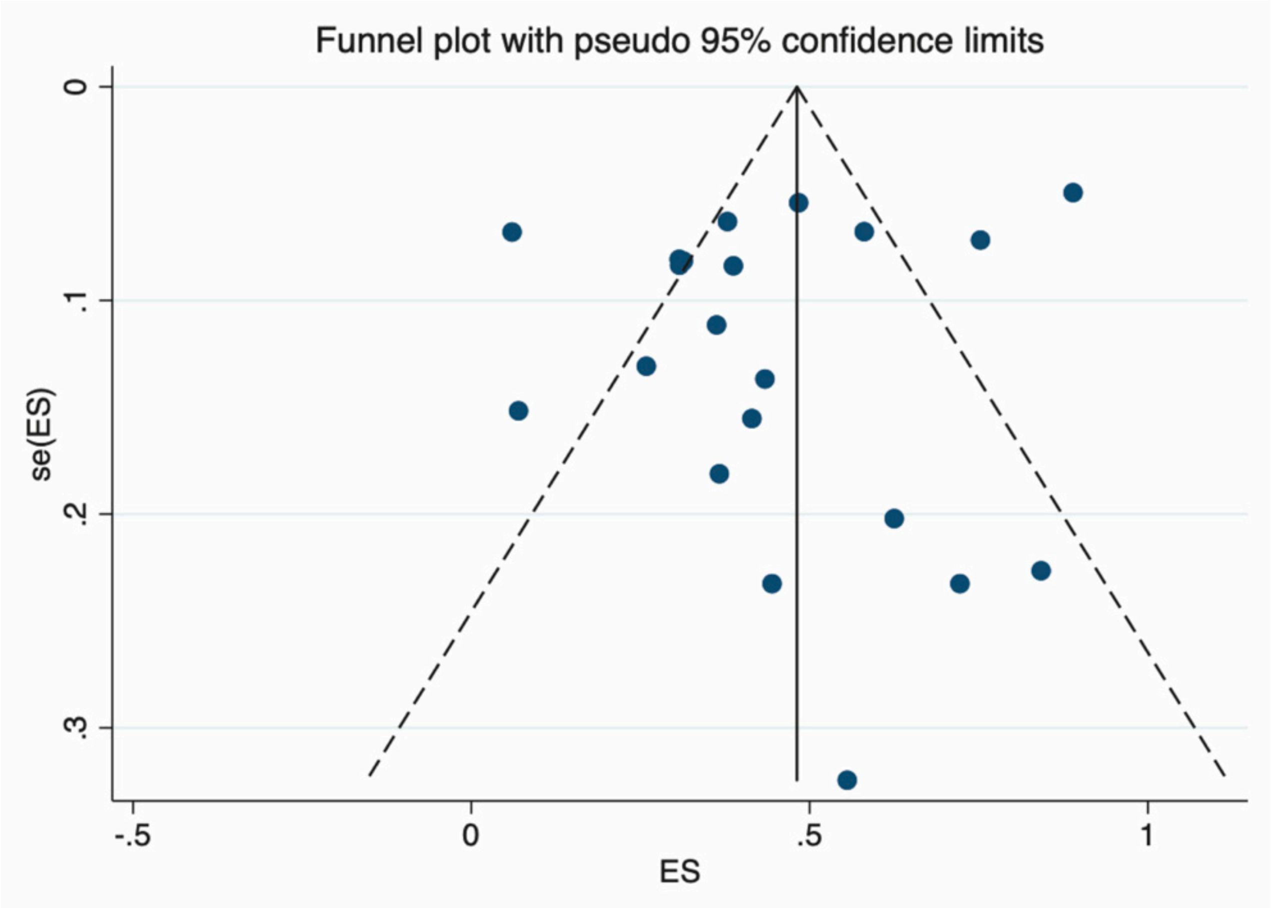

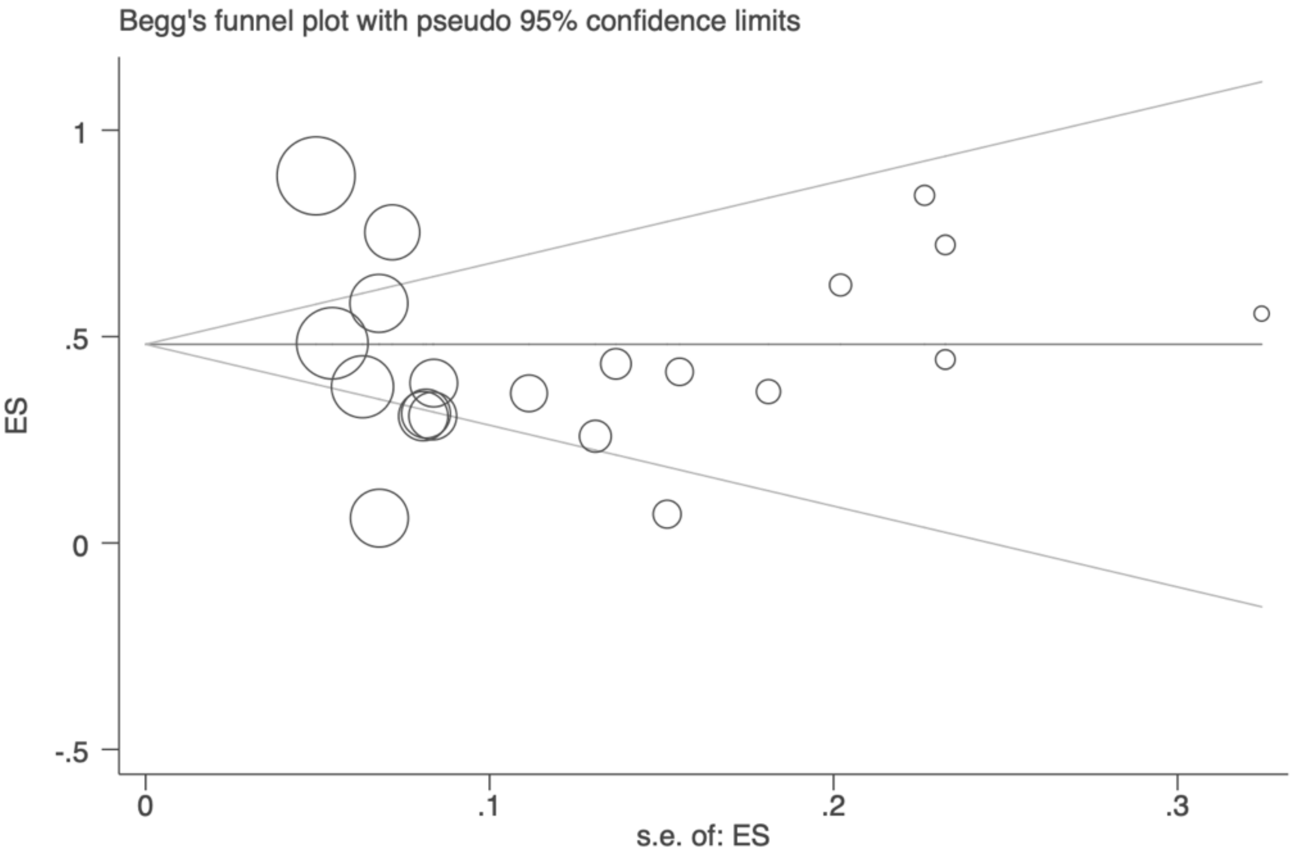

Begg's funnel plot with pseudo 95% confidence limits
1
.5
0
-.5
ES
0
.1
.2
.3
s.e. of: ES

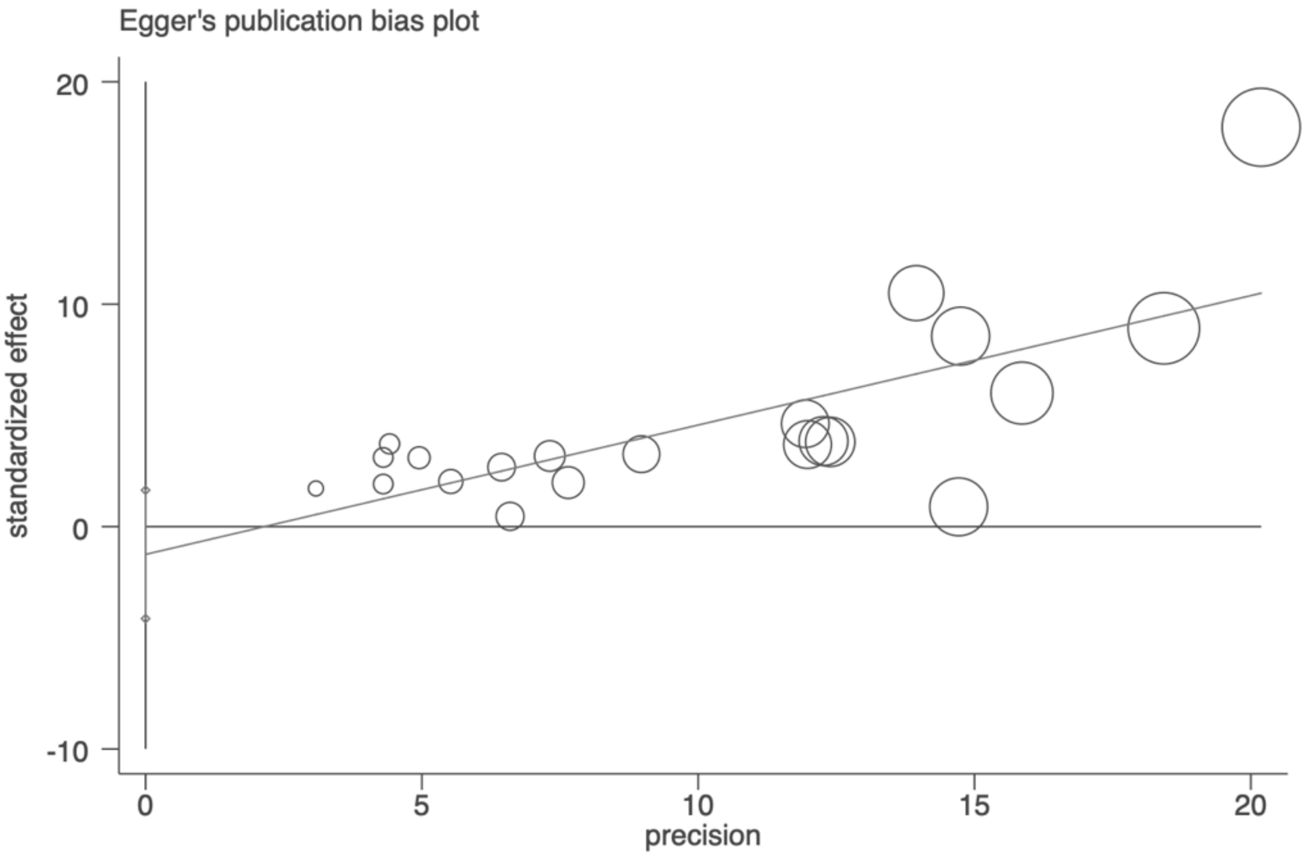
Egger's publication bias plot
standardized effect
precision
20
10
0
-10
0
5
10
15
20

Supplementary Figure S17: Publication bias of cs-free remission at induction in Crohn’s disease

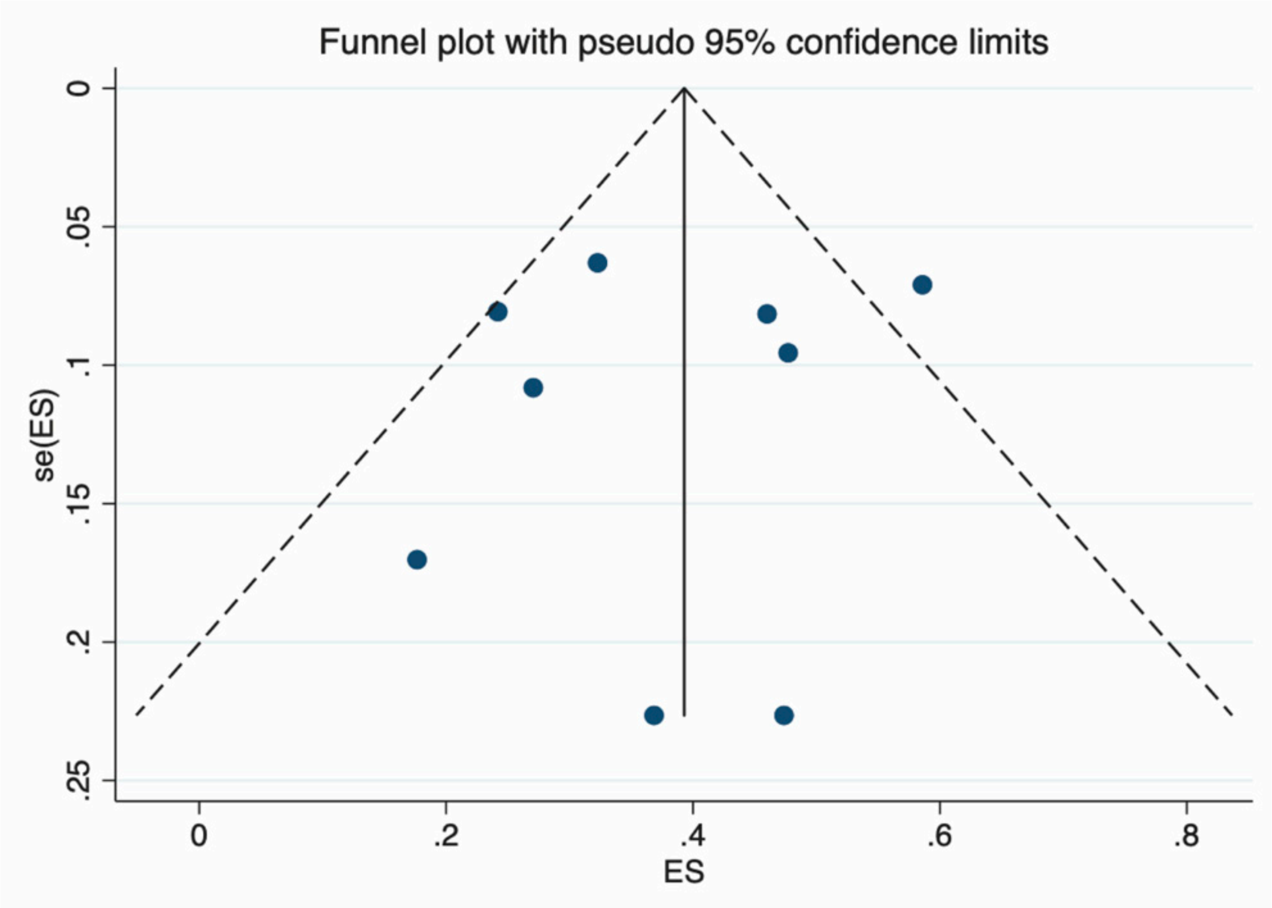

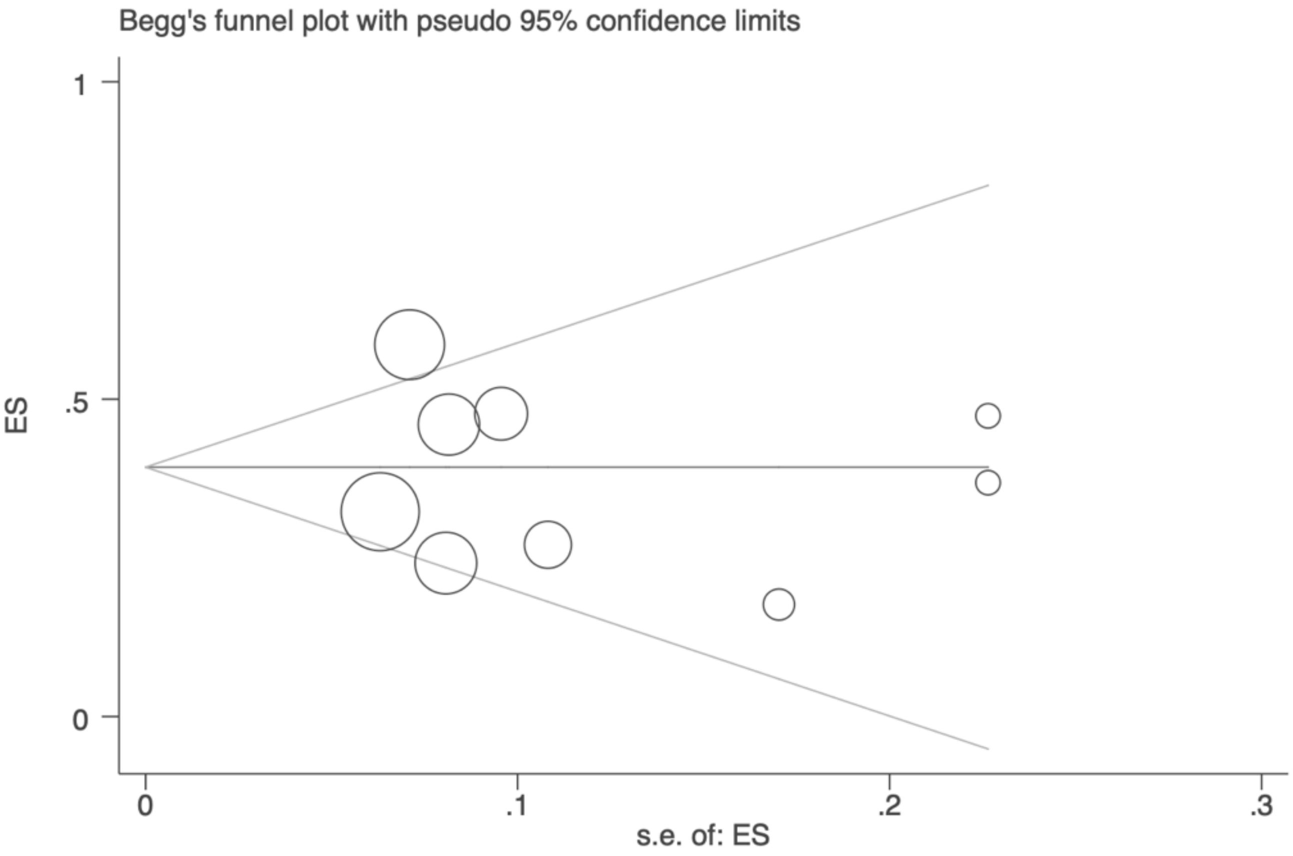

Begg's funnel plot with pseudo 95% confidence limits
1
.5
0
ES
0
.1
.2
.3
s.e. of: ES

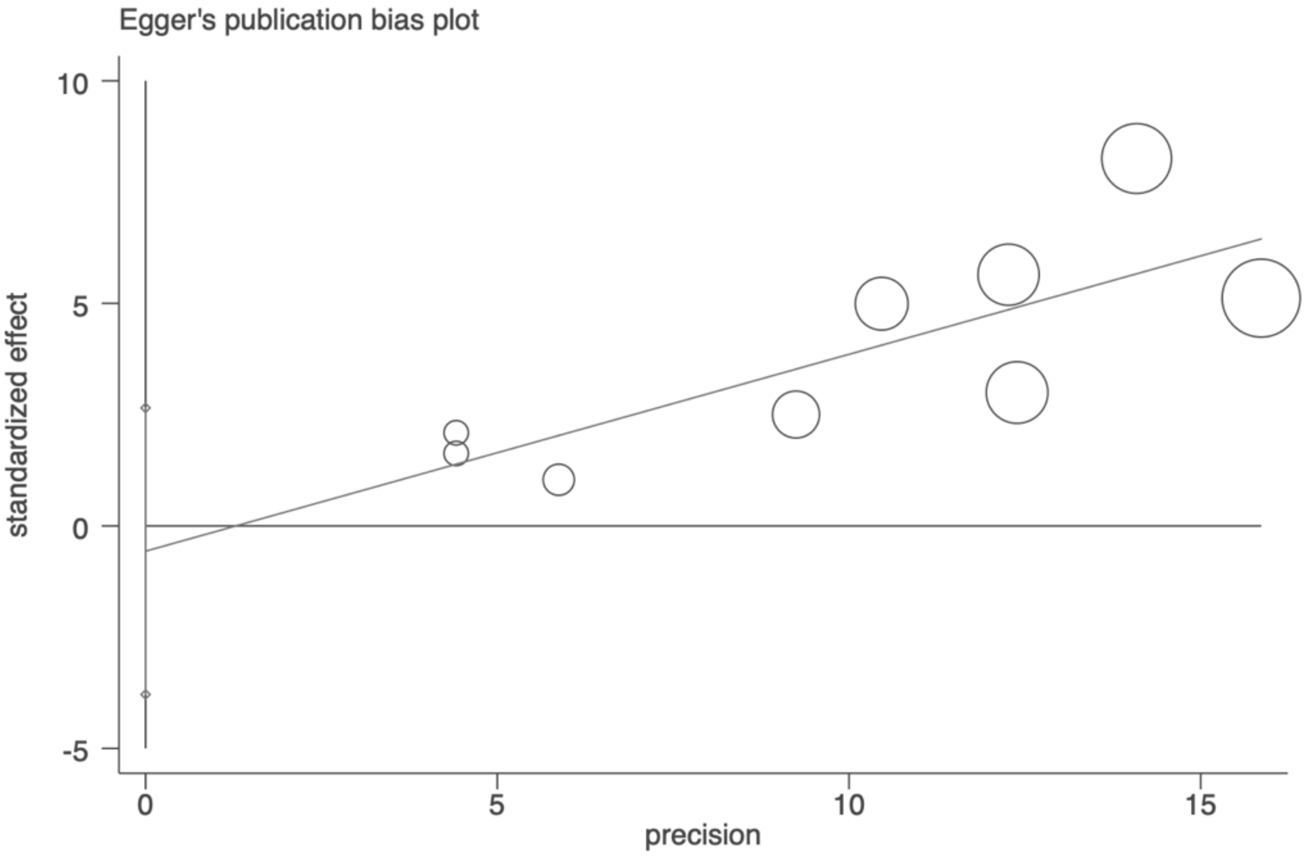

Egger's publication bias plot
10
5
0
-5
standardized effect
0
5
10
15
precision

Supplementary Figure S18: Publication bias of clinical response in the 24-week maintenance in Crohn's disease

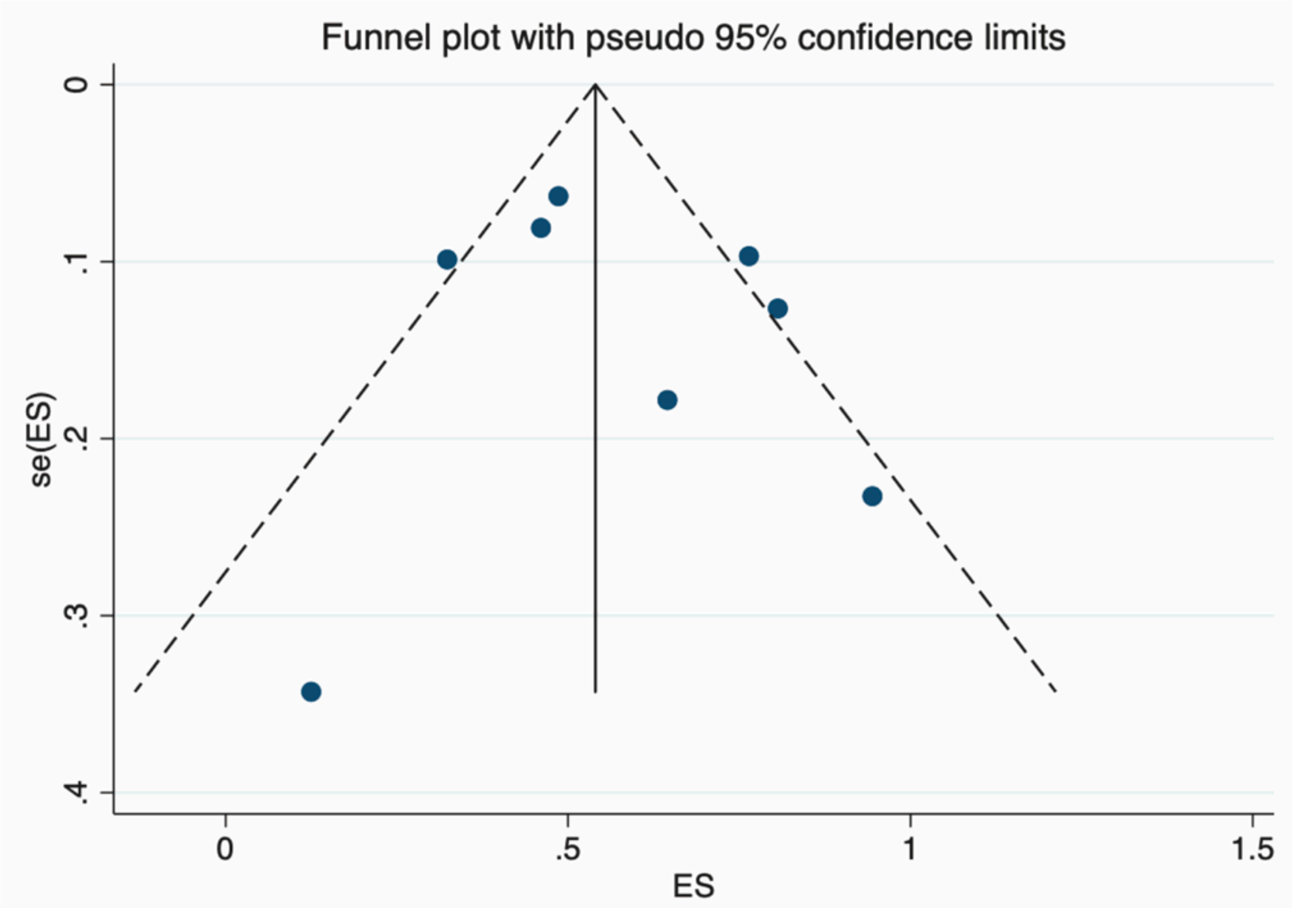

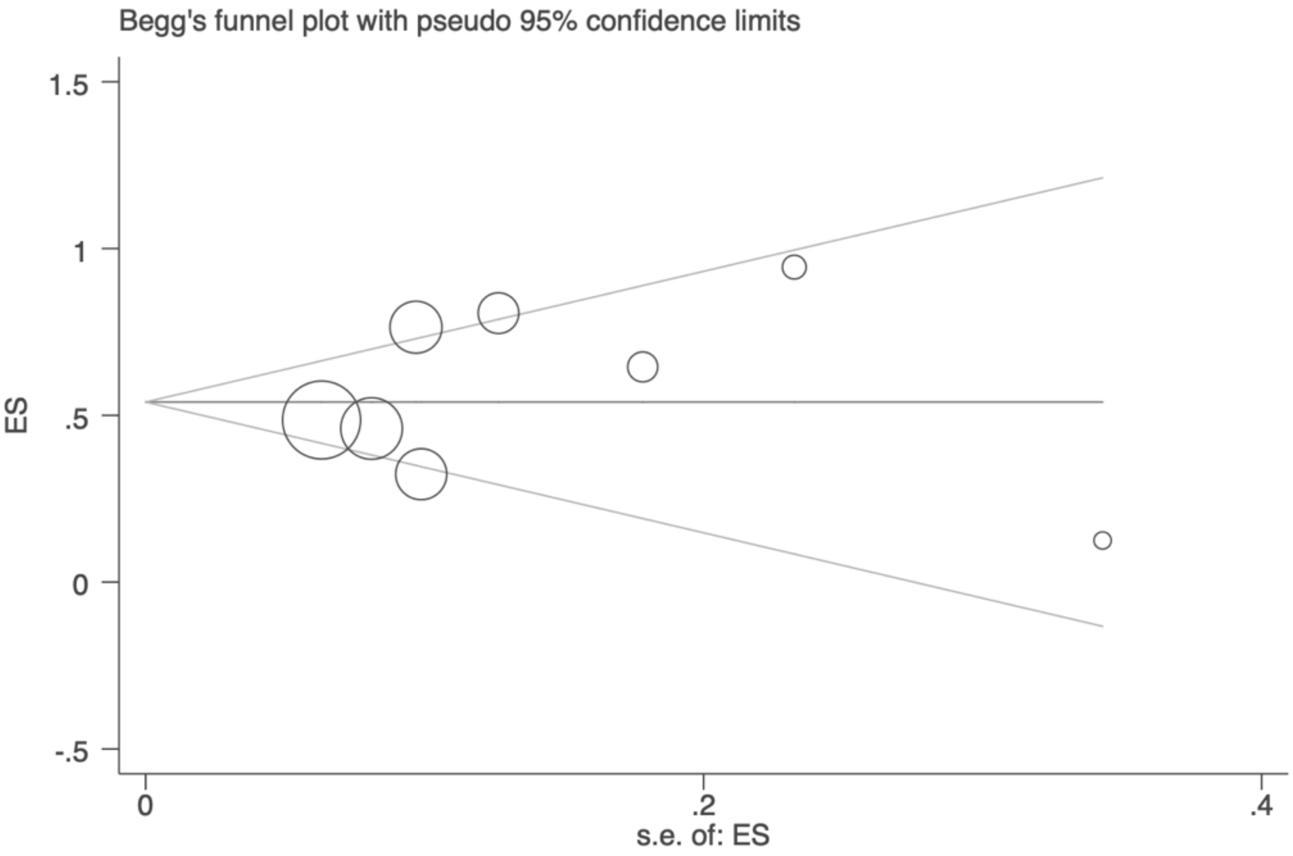
Begg's funnel plot with pseudo 95% confidence limits
1.5
1
.5
0
-.5
ES
0
.2
.4
s.e. of: ES

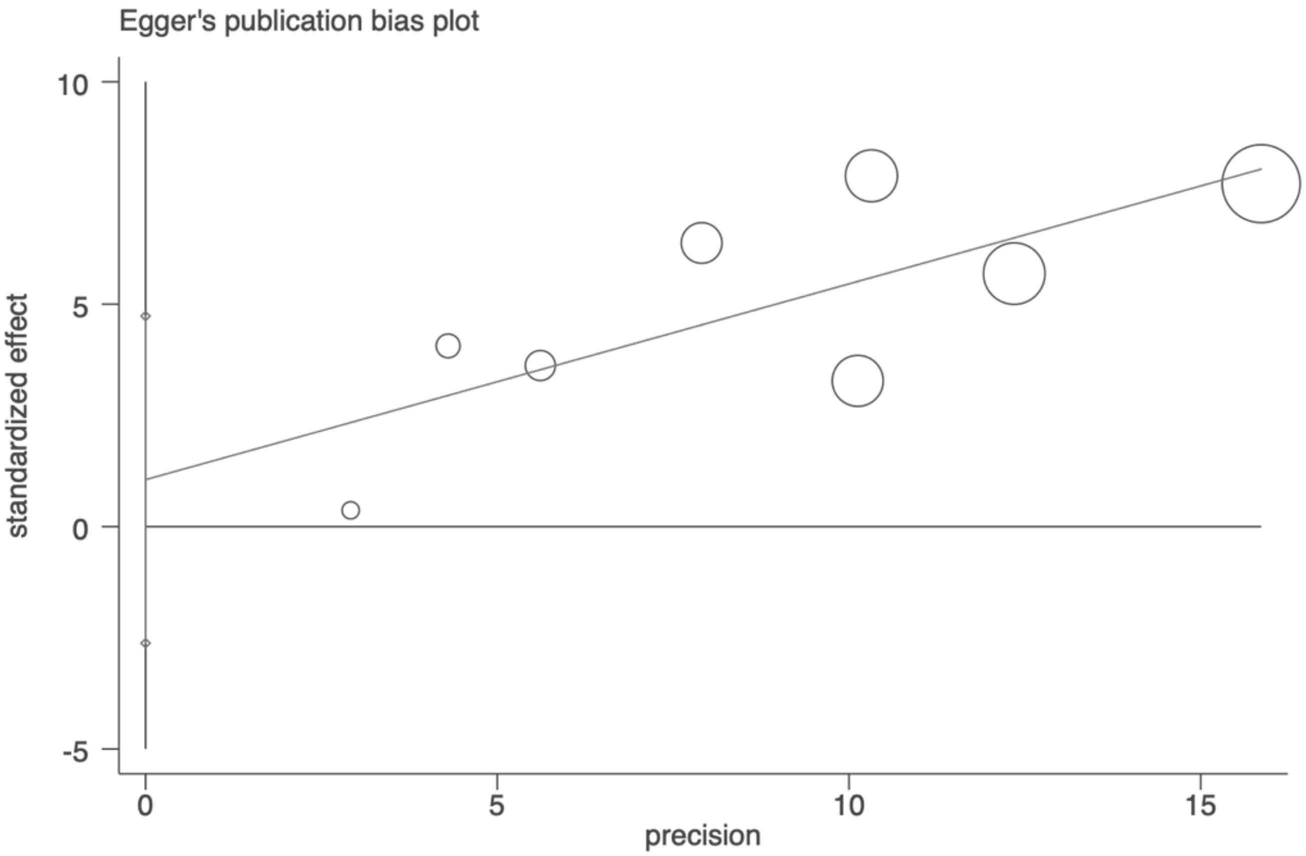

Egger's publication bias plot
10
5
0
-5
standardized effect
0
5
10
15
precision

Supplementary Figure S19: Publication bias of clinical remission in the 24-week maintenance in Crohn’s disease

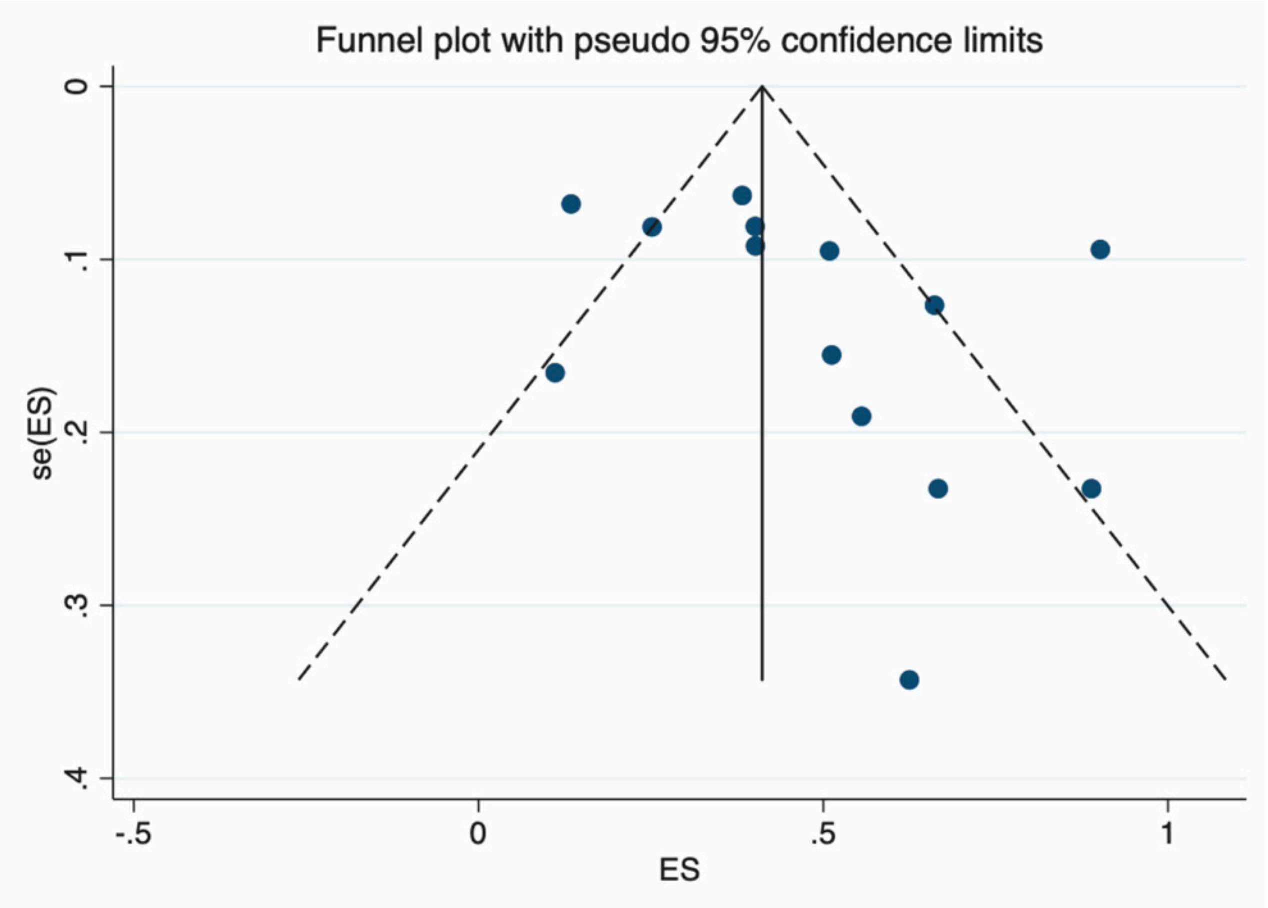

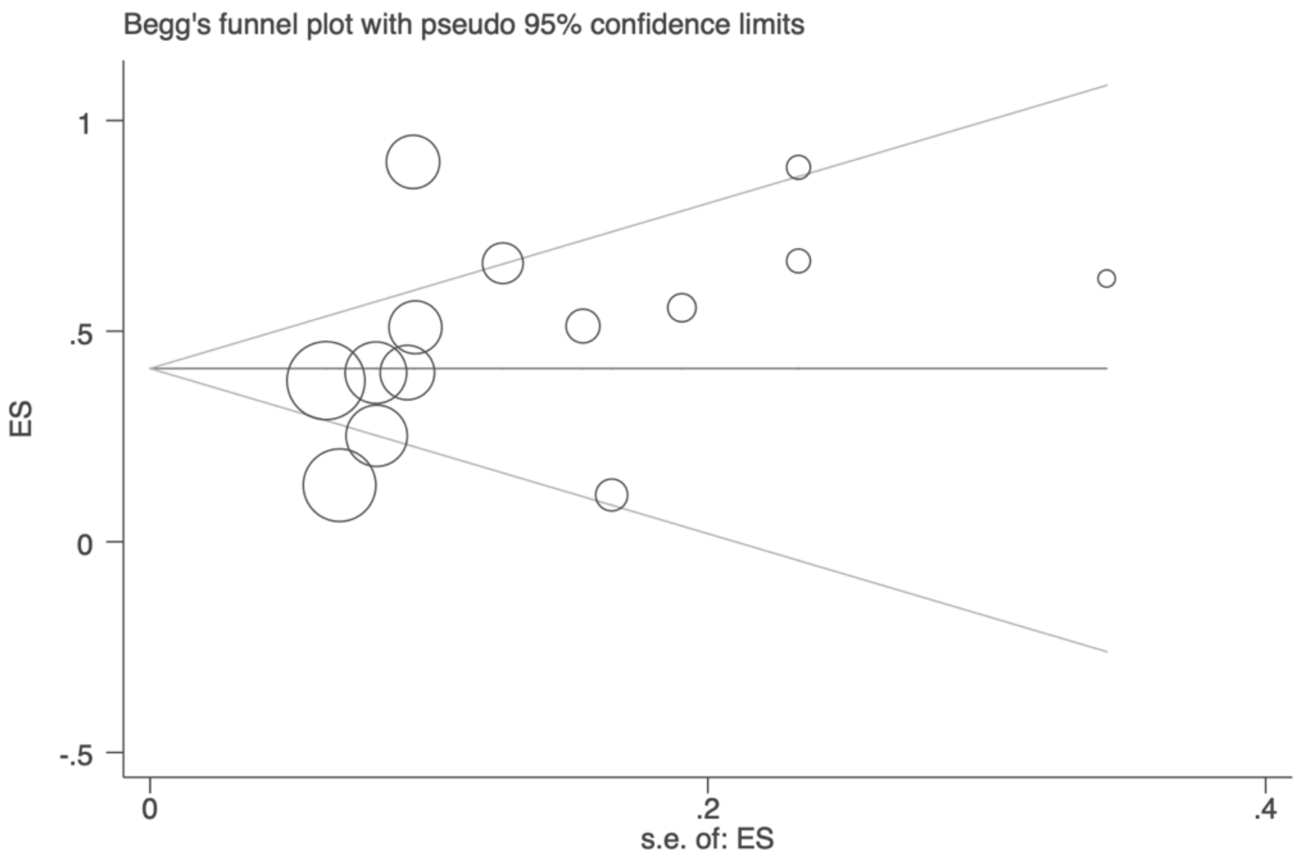
Begg's funnel plot with pseudo 95% confidence limits
1
.5
0
-.5
ES
0
.2
.4
s.e. of: ES

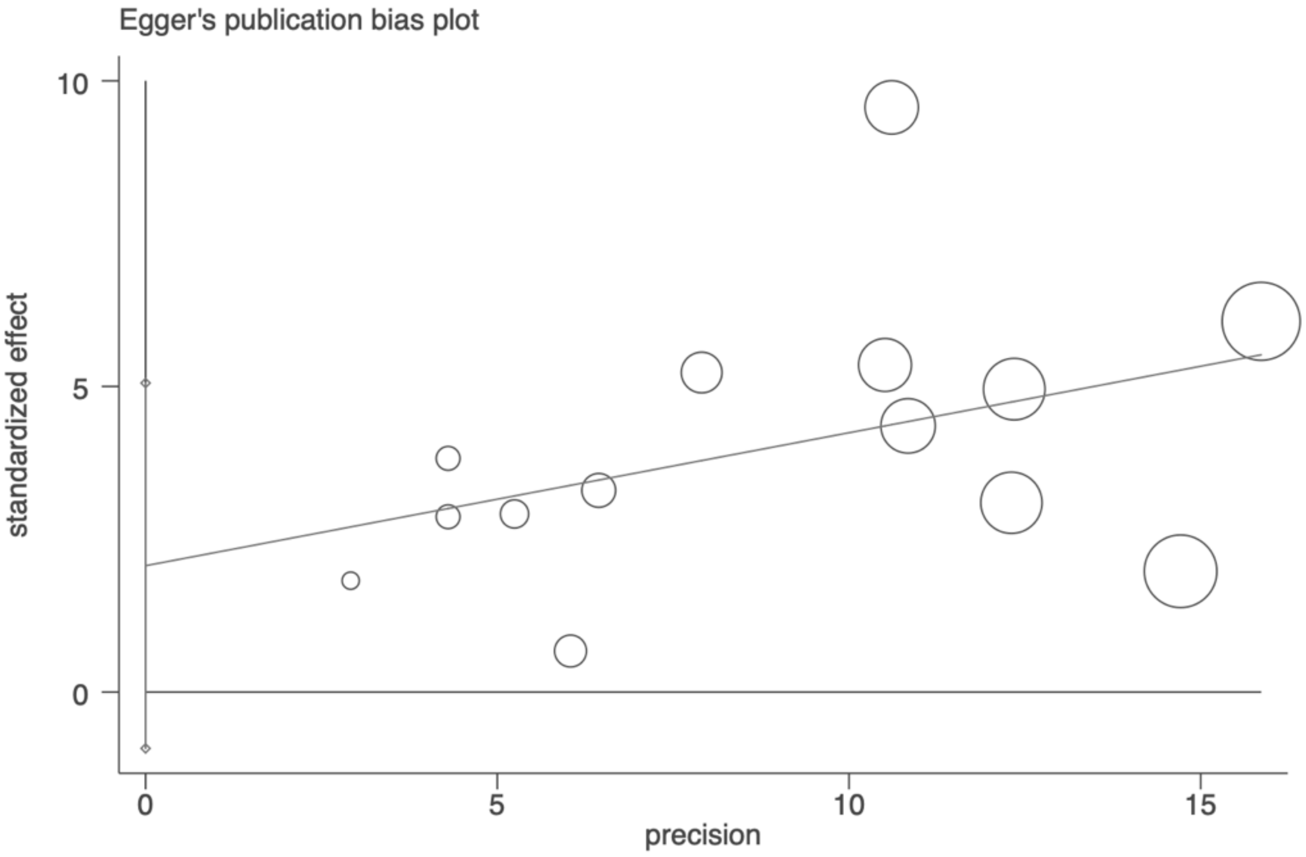

Egger's publication bias plot
standardized effect
precision
10
5
0
0
5
10
15

Supplementary Figure S20: Publication bias of cs-free remission in the 24-week maintenance in Crohn's disease

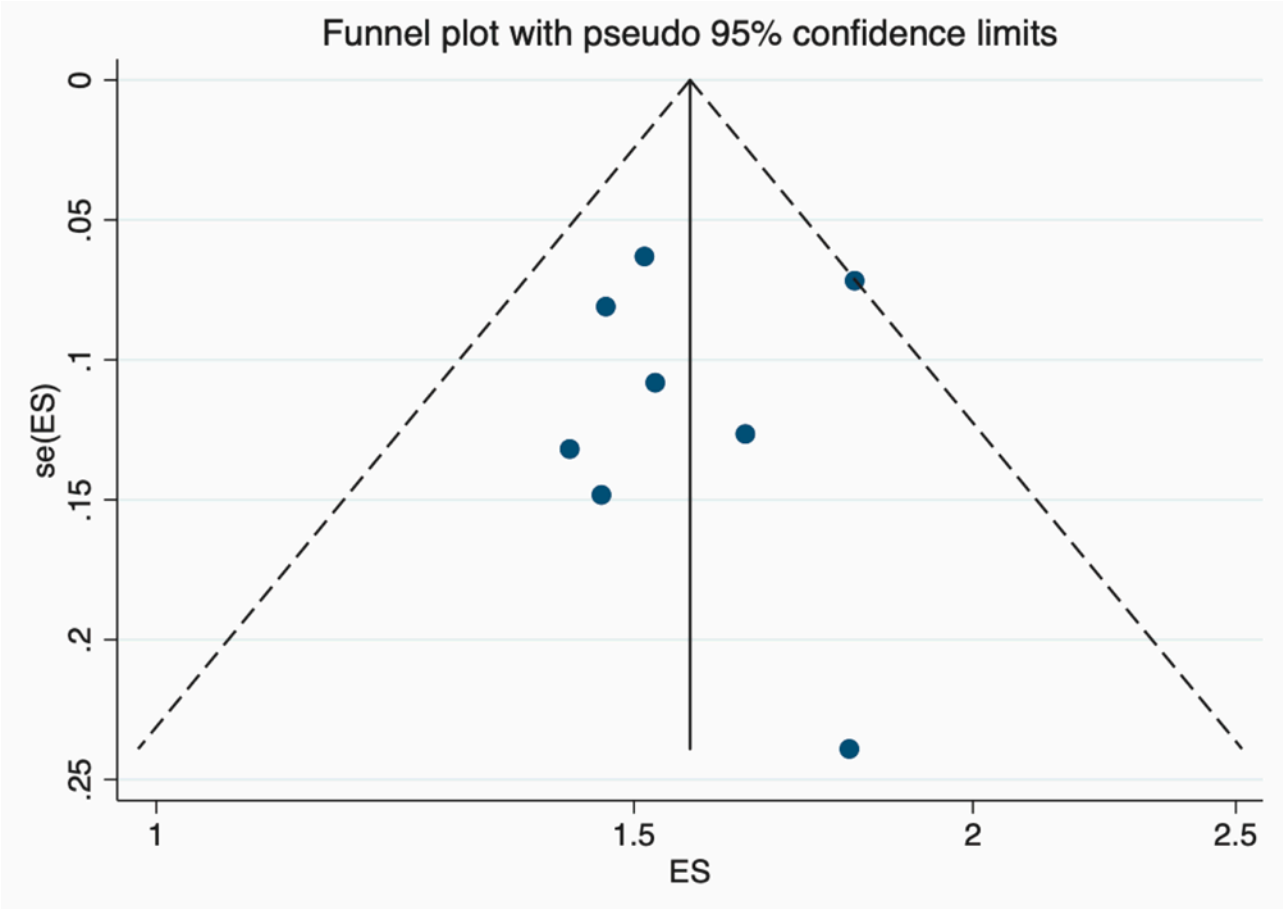

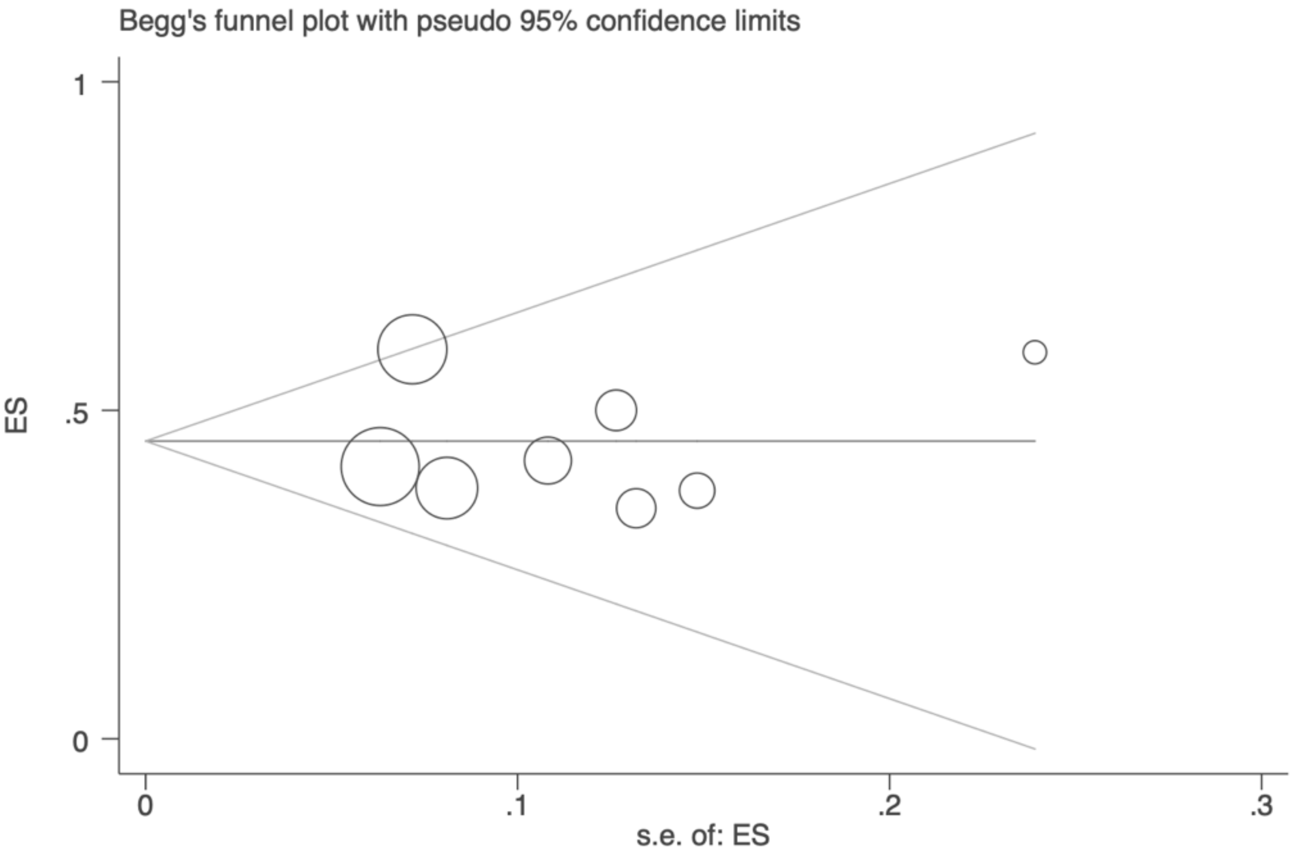

Begg's funnel plot with pseudo 95% confidence limits
1
.5
0
ES
0
.1
.2
.3
s.e. of: ES

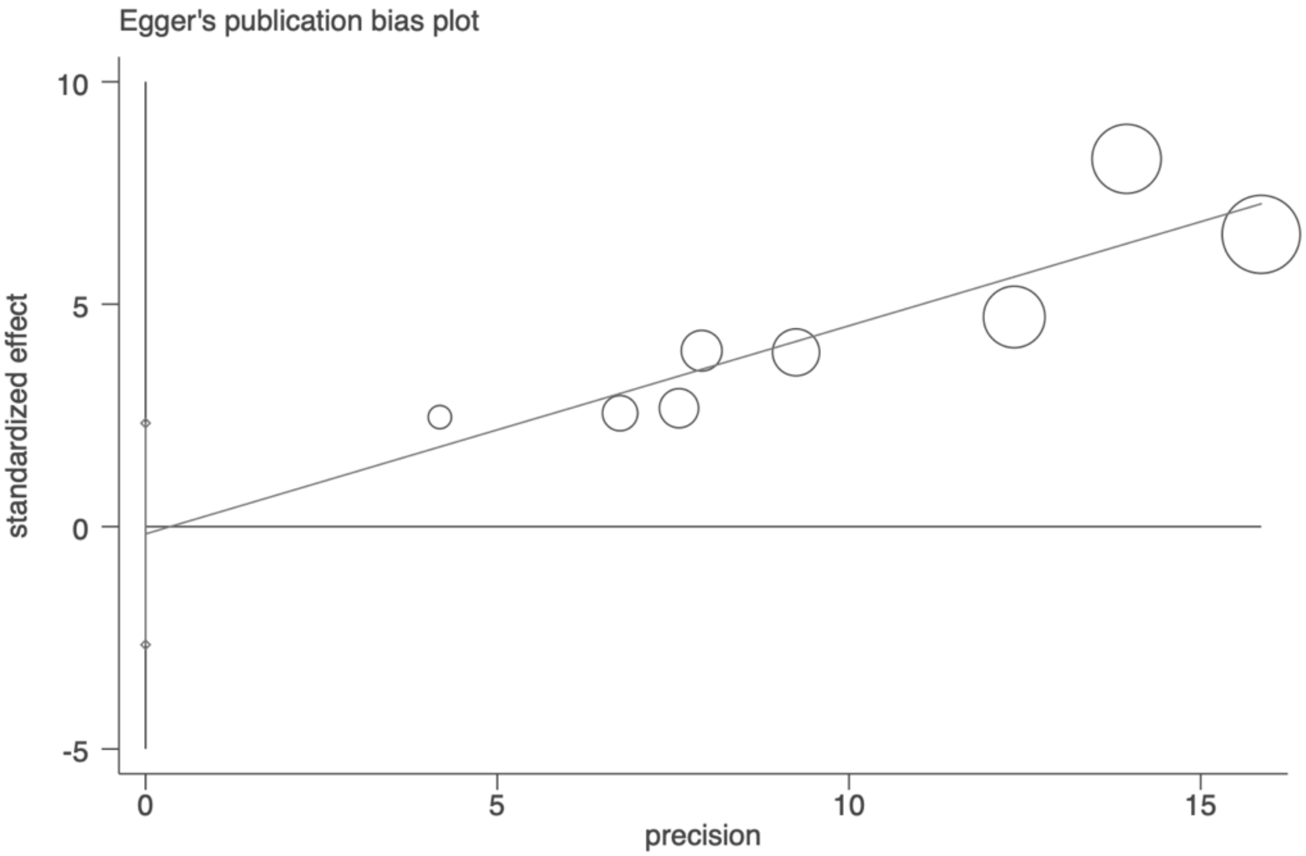
Egger's publication bias plot
standardized effect
precision
10
5
0
-5
0
5
10
15

Supplementary Figure S21: Publication bias of clinical resopone in the 1-year maintenance in Crohn's disease

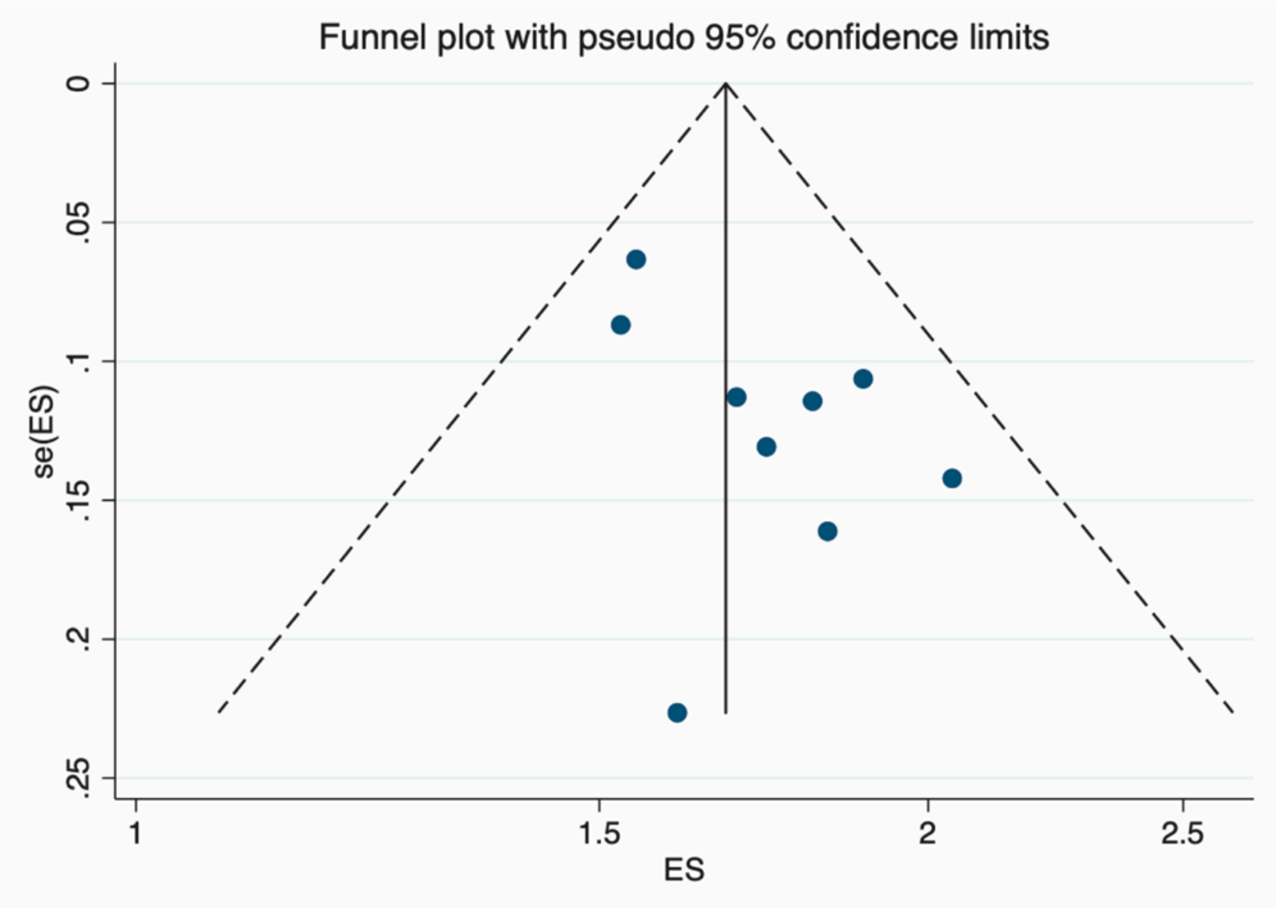

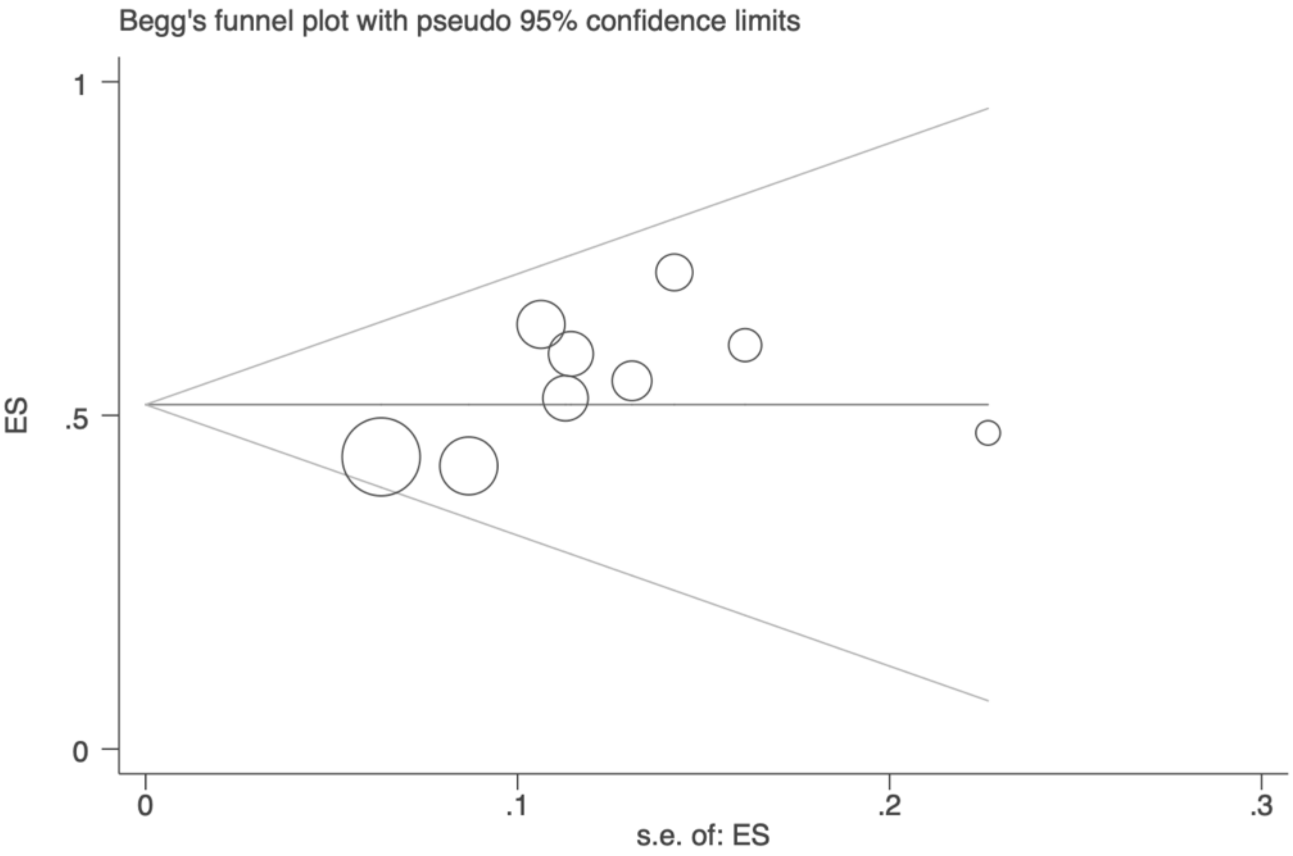

Begg's funnel plot with pseudo 95% confidence limits
1
.5
0
ES
0
.1
.2
.3
s.e. of: ES

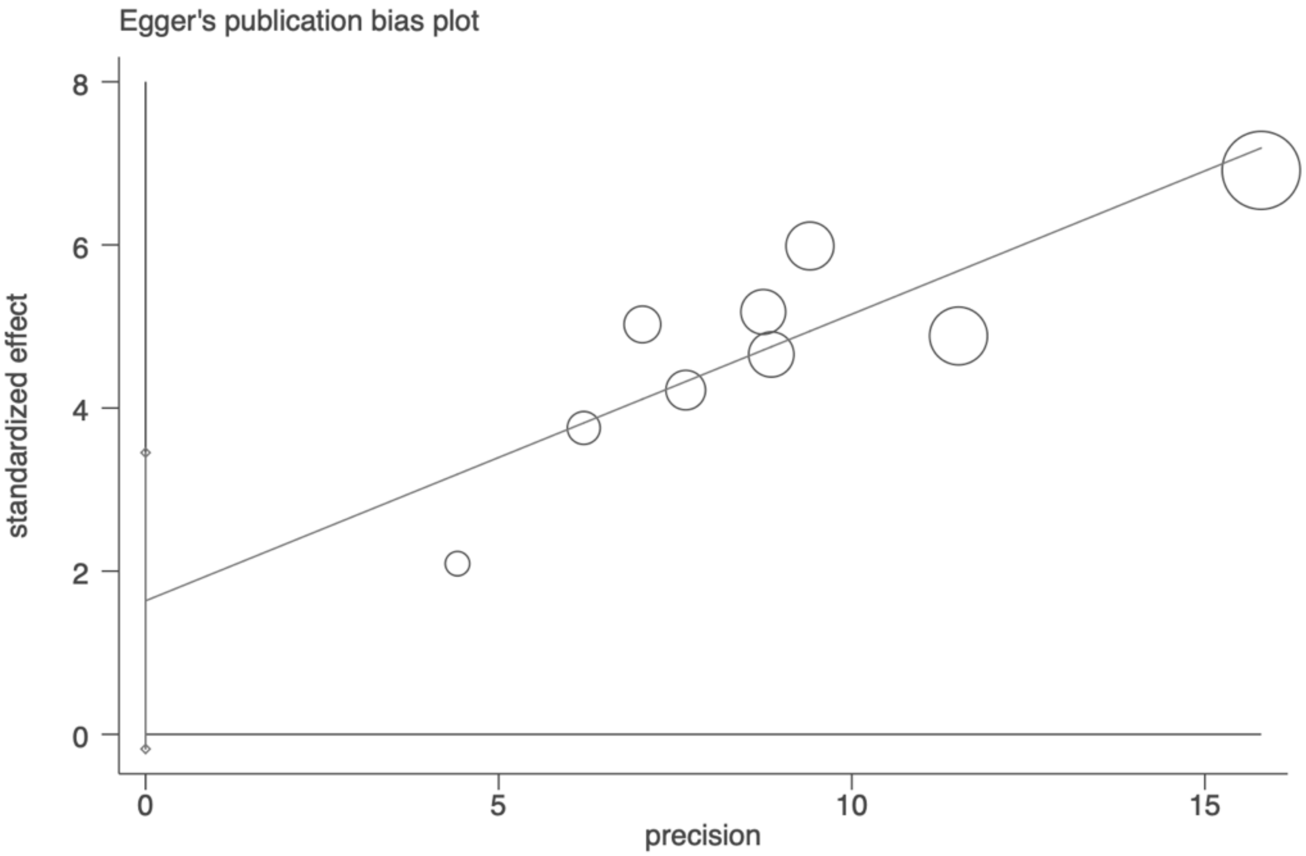
Egger's publication bias plot
standardized effect
precision
8
6
4
2
0
0
5
10
15

Supplementary Figure S22: Publication bias of clinical remission in the 1-year maintenance in Crohn's disease

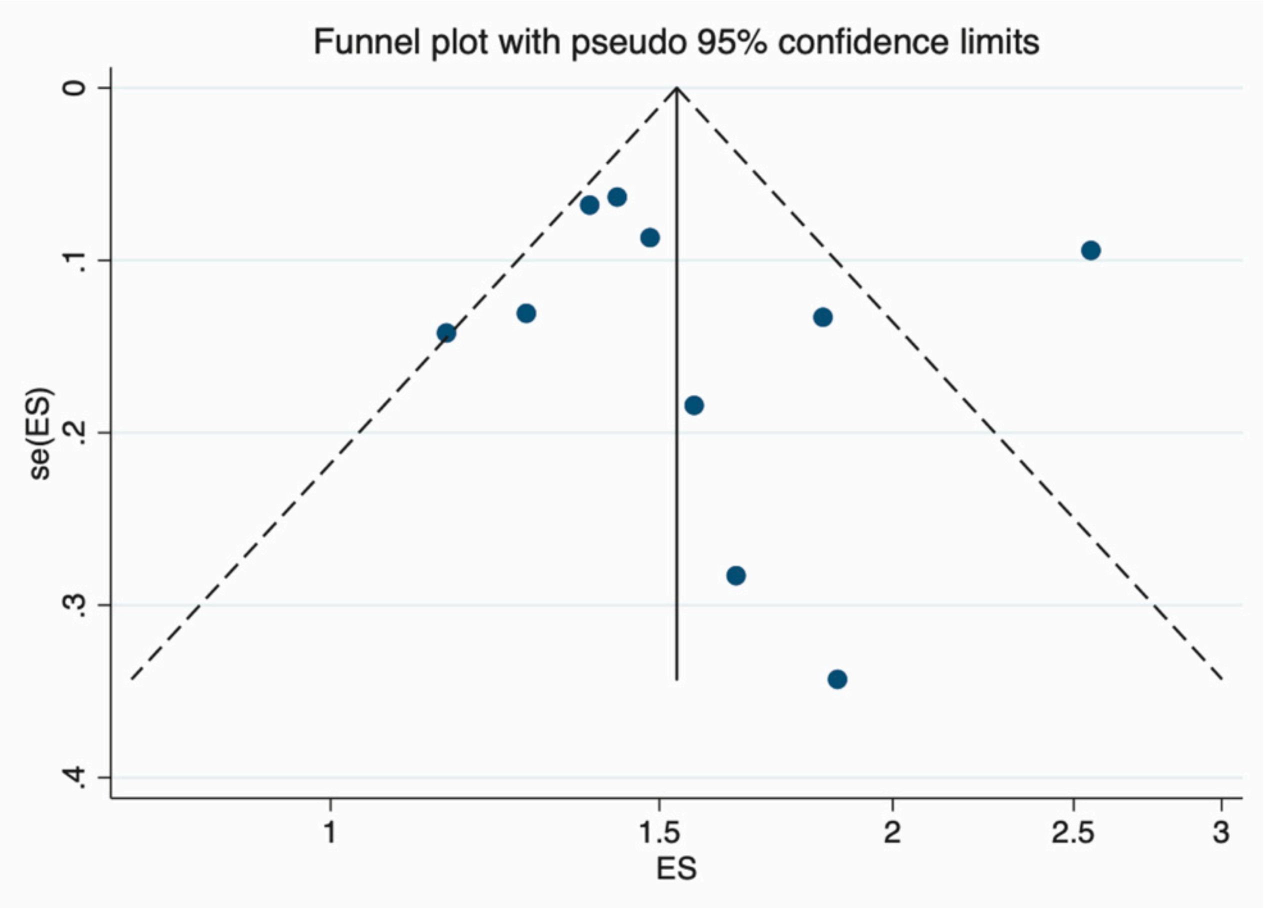

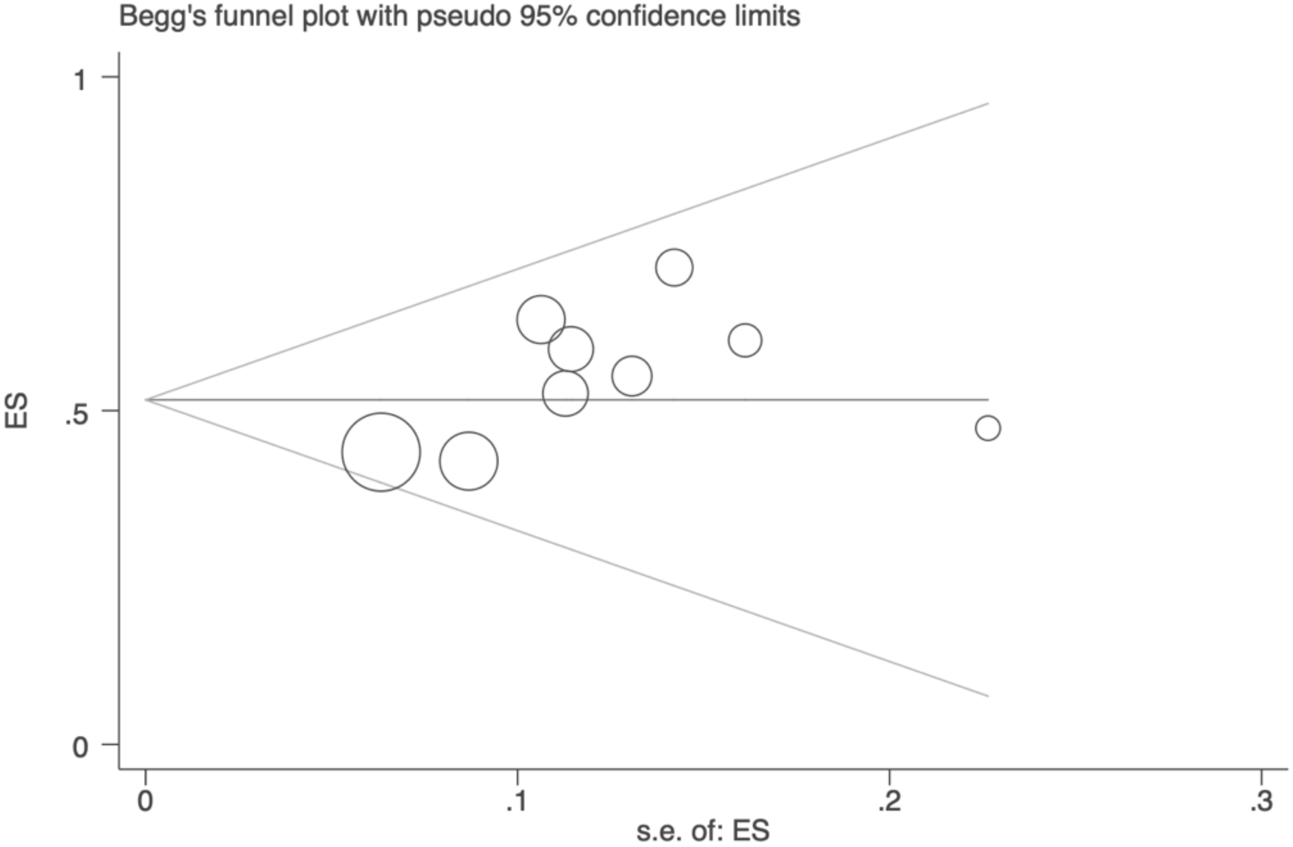
Begg's funnel plot with pseudo 95% confidence limits
1
.5
0
ES
0
.1
.2
.3
s.e. of: ES

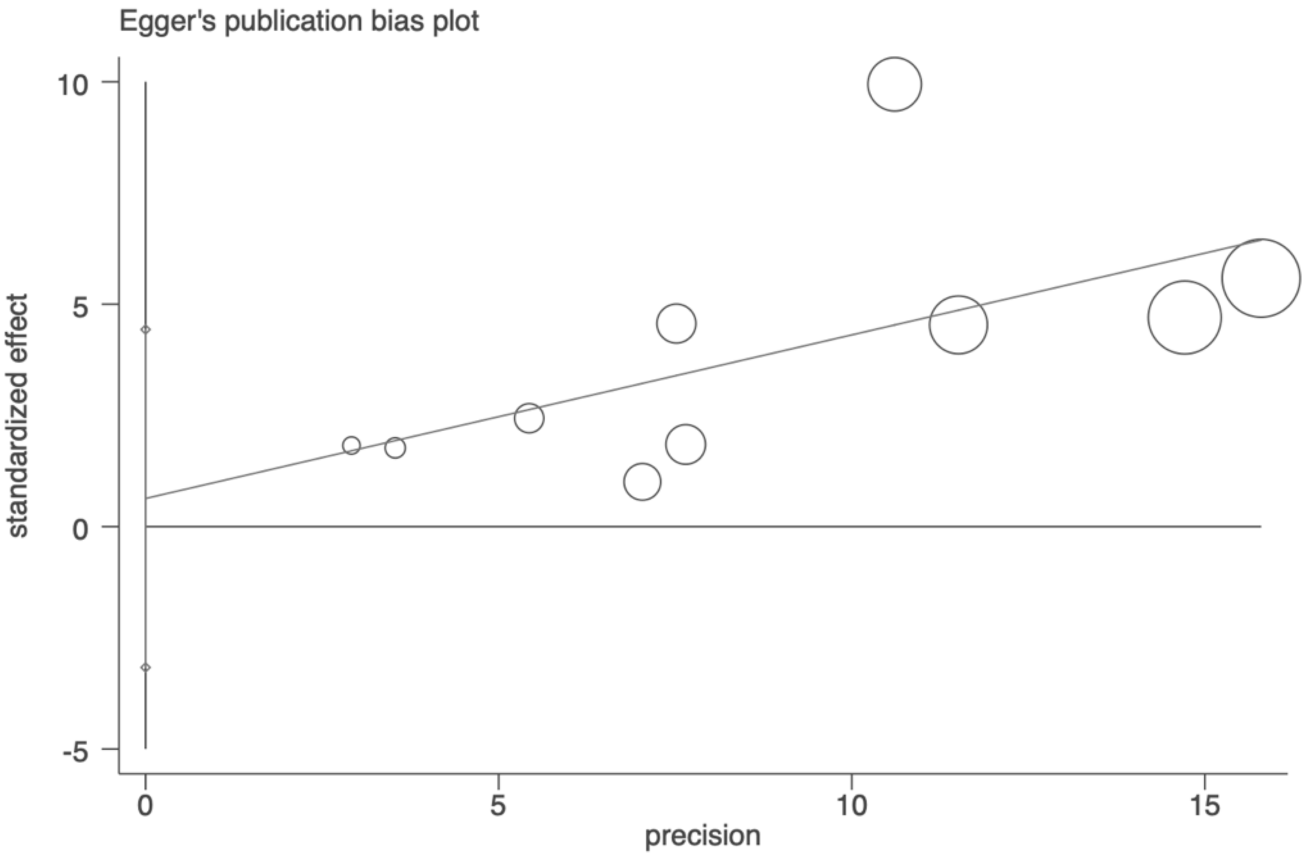

Egger's publication bias plot
standardized effect
10
5
0
-5
0
5
10
15
precision

Supplementary Figure S23: Publication bias of endoscopic response in the 1-year maintenance in Crohn's disease

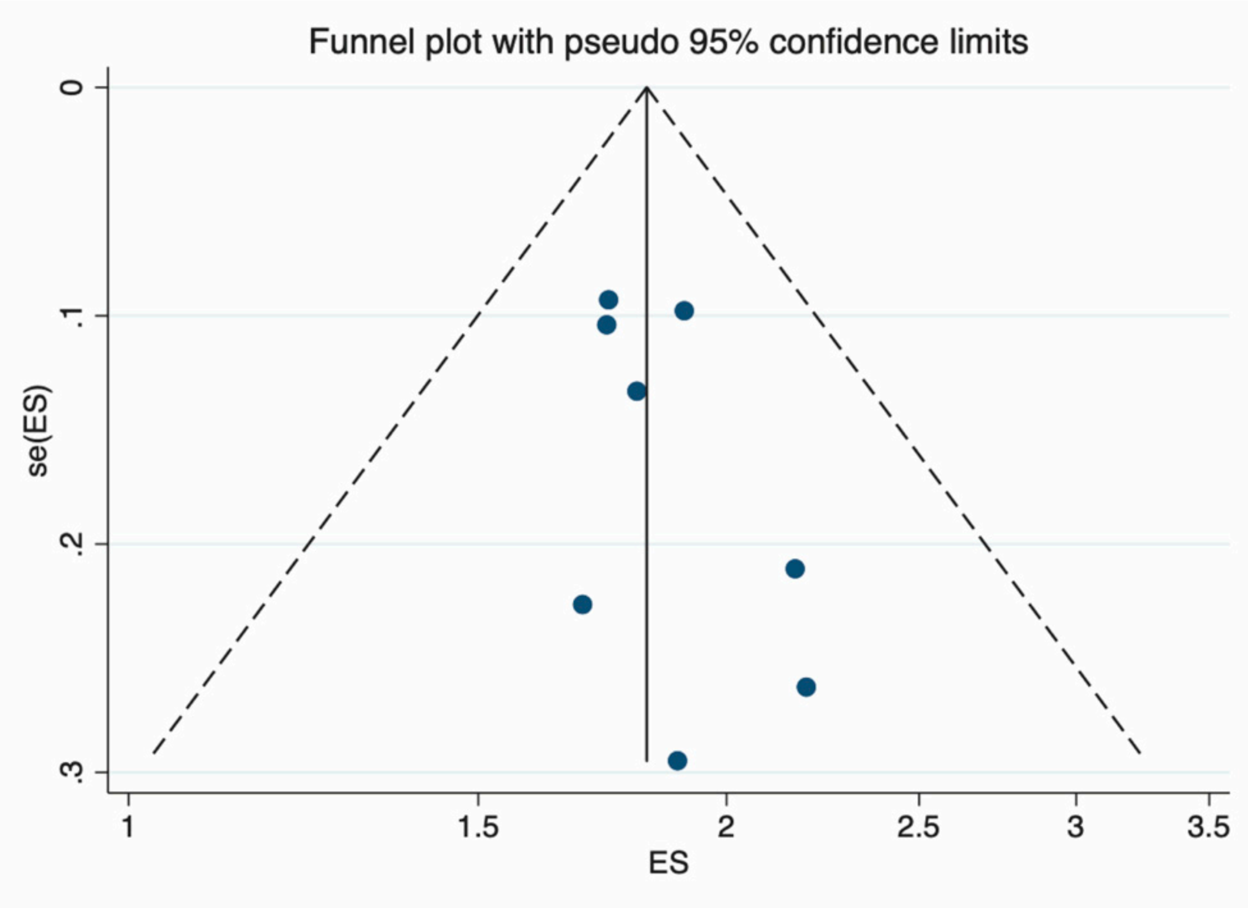

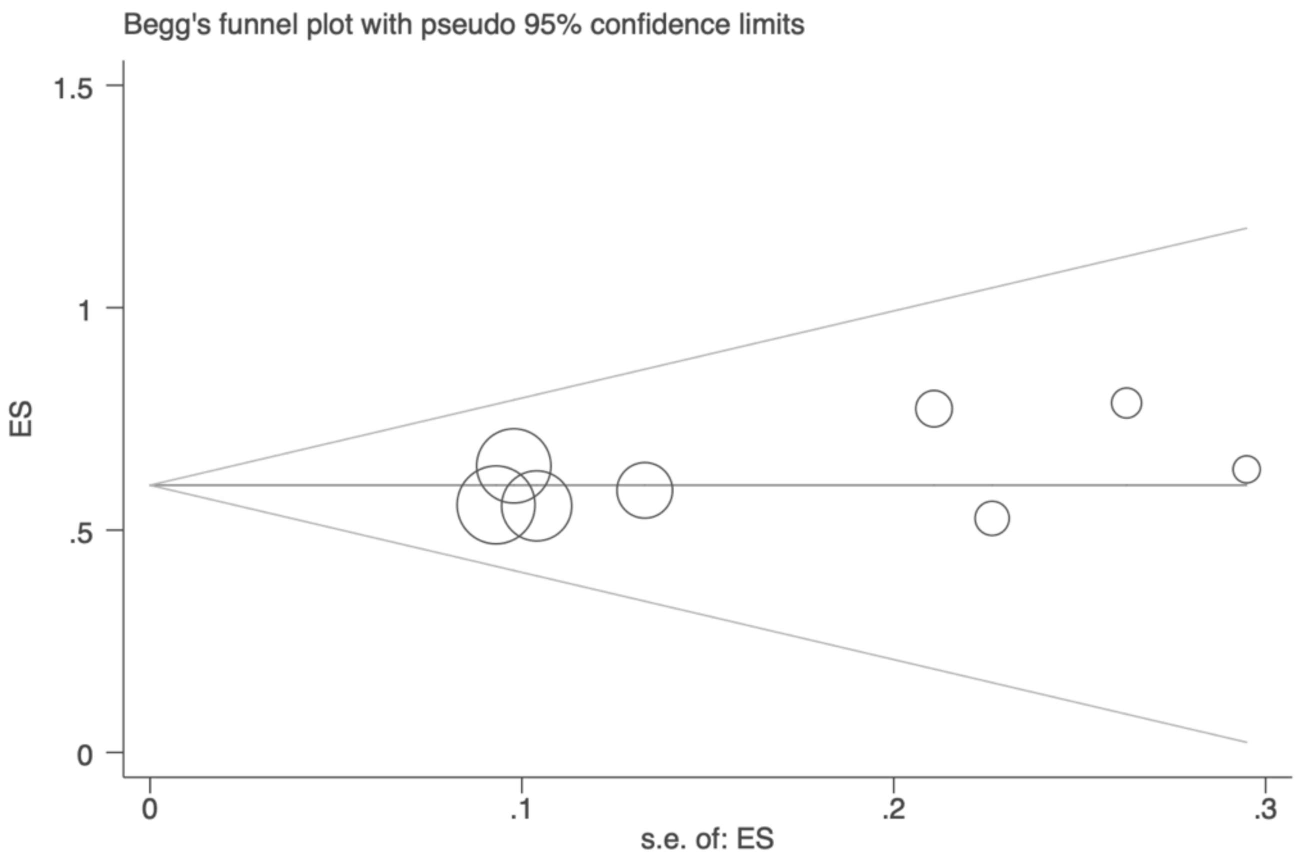
Begg's funnel plot with pseudo 95% confidence limits
1.5
1
.5
0
ES
0
.1
.2
.3
s.e. of: ES

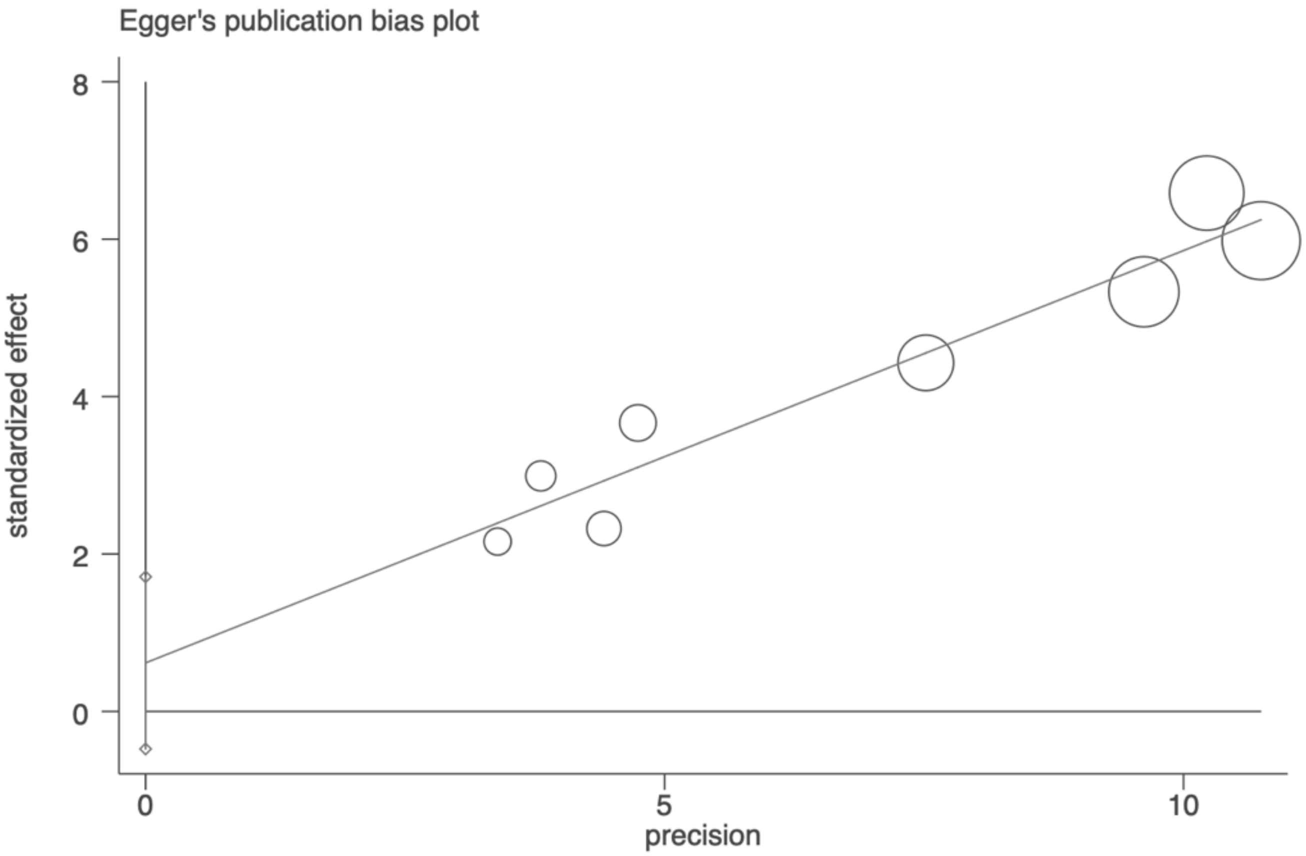
Egger's publication bias plot
standardized effect
8
6
4
2
0
0
5
10
precision

Supplementary Figure S24: Publication bias of endoscopic remission in the 1-year maintenance in Crohn’s disease

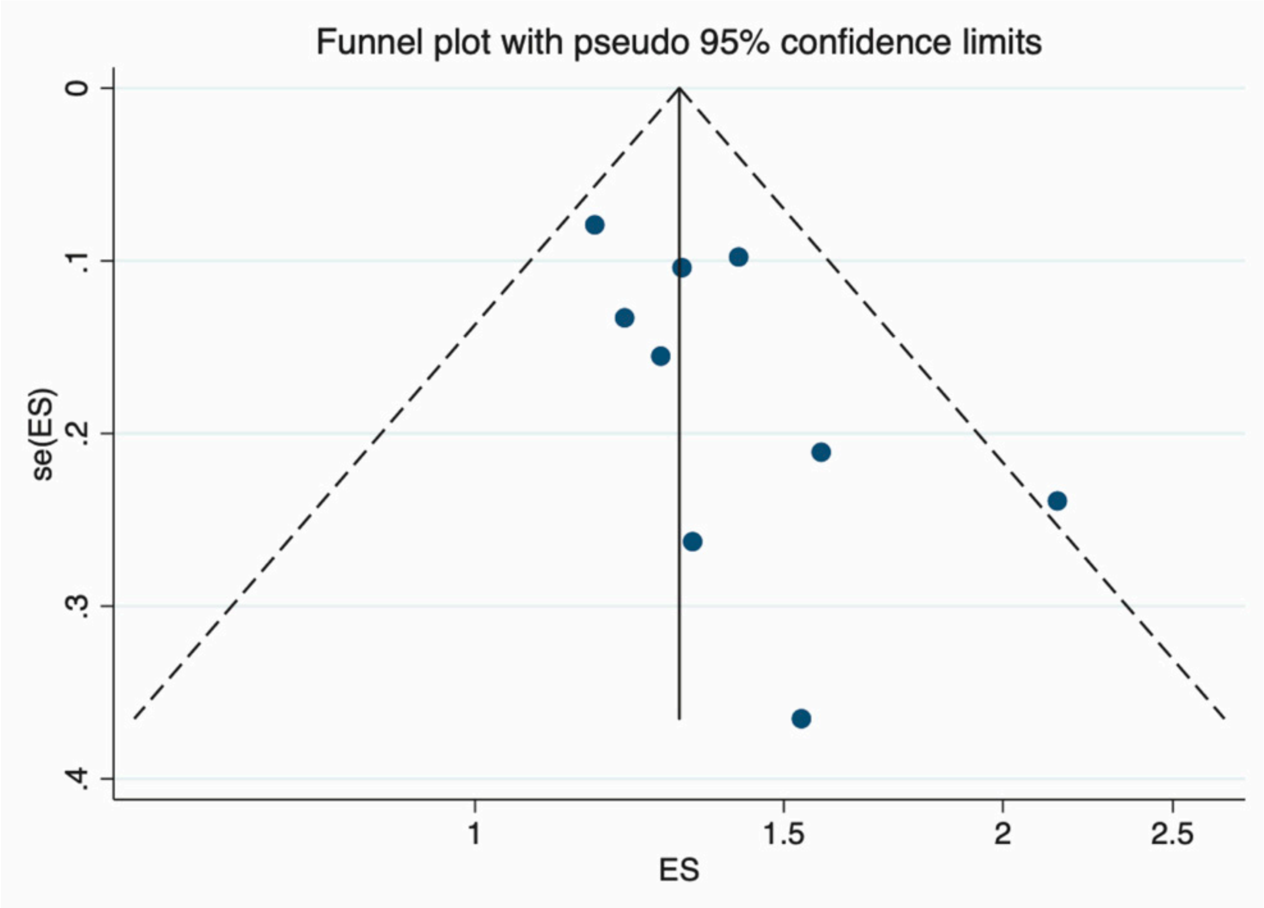

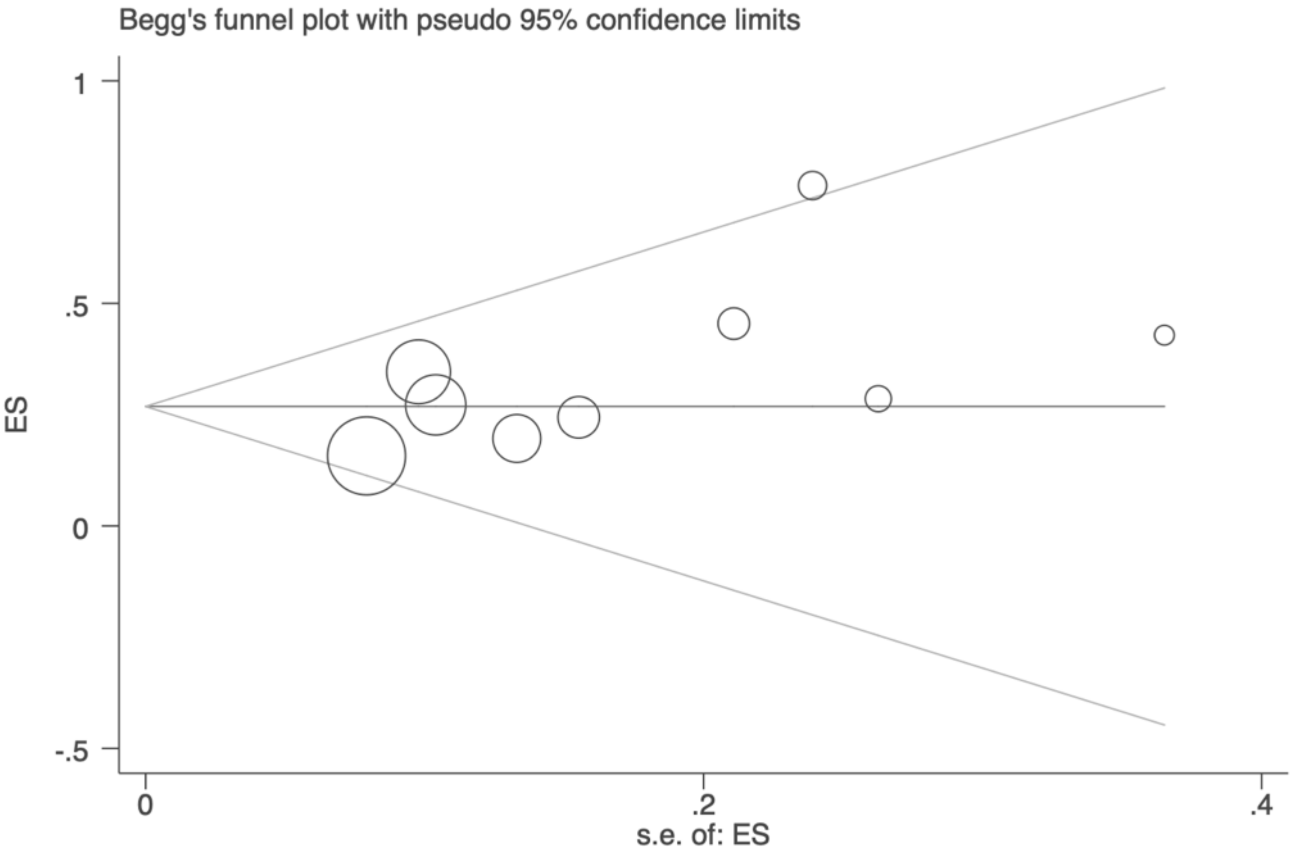
Begg's funnel plot with pseudo 95% confidence limits
1
.5
0
-.5
ES
0
.2
.4
s.e. of: ES

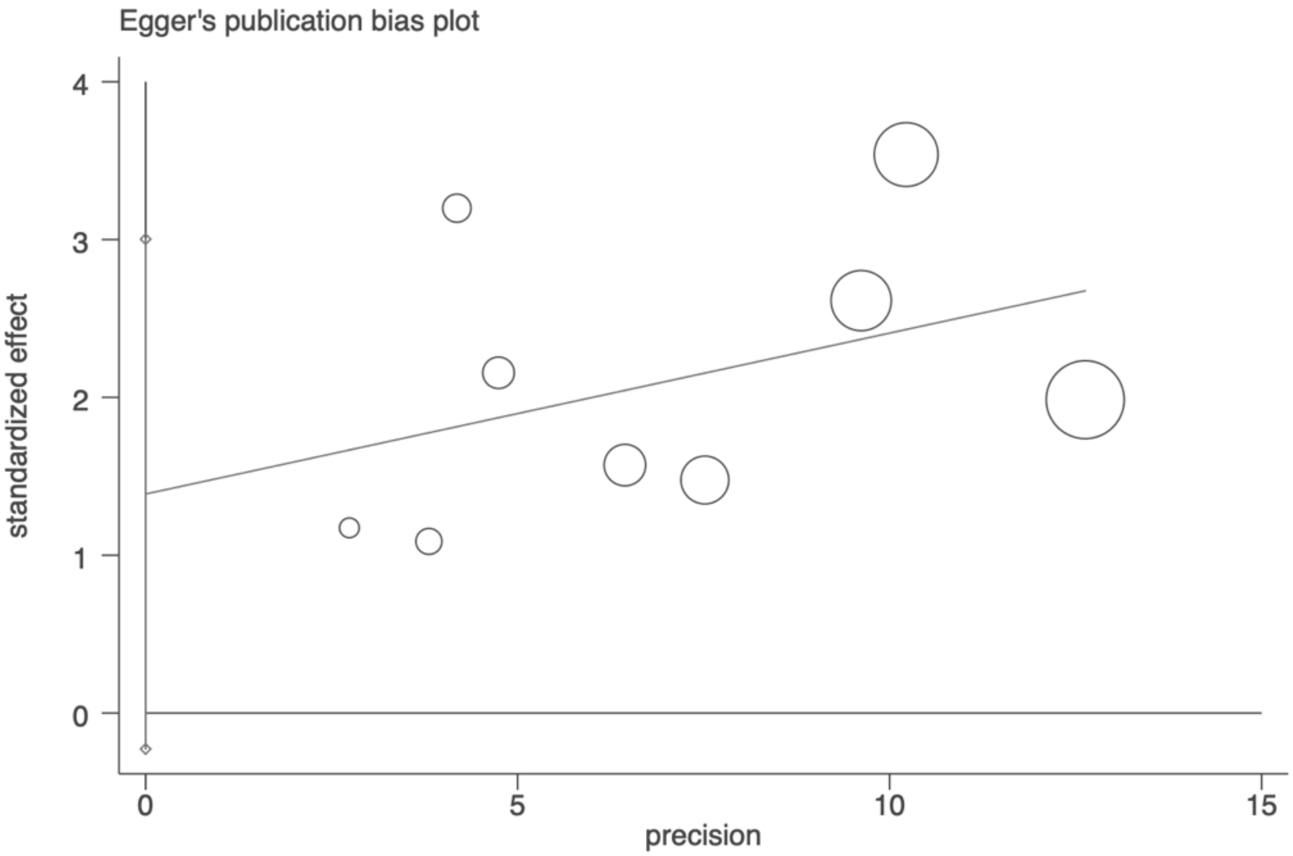

Egger's publication bias plot
standardized effect
precision
0
1
2
3
4
0
5
10
15

Supplementary Figure S25: Publication bias of mucosal healing in the 1-year maintenance in Crohn's disease

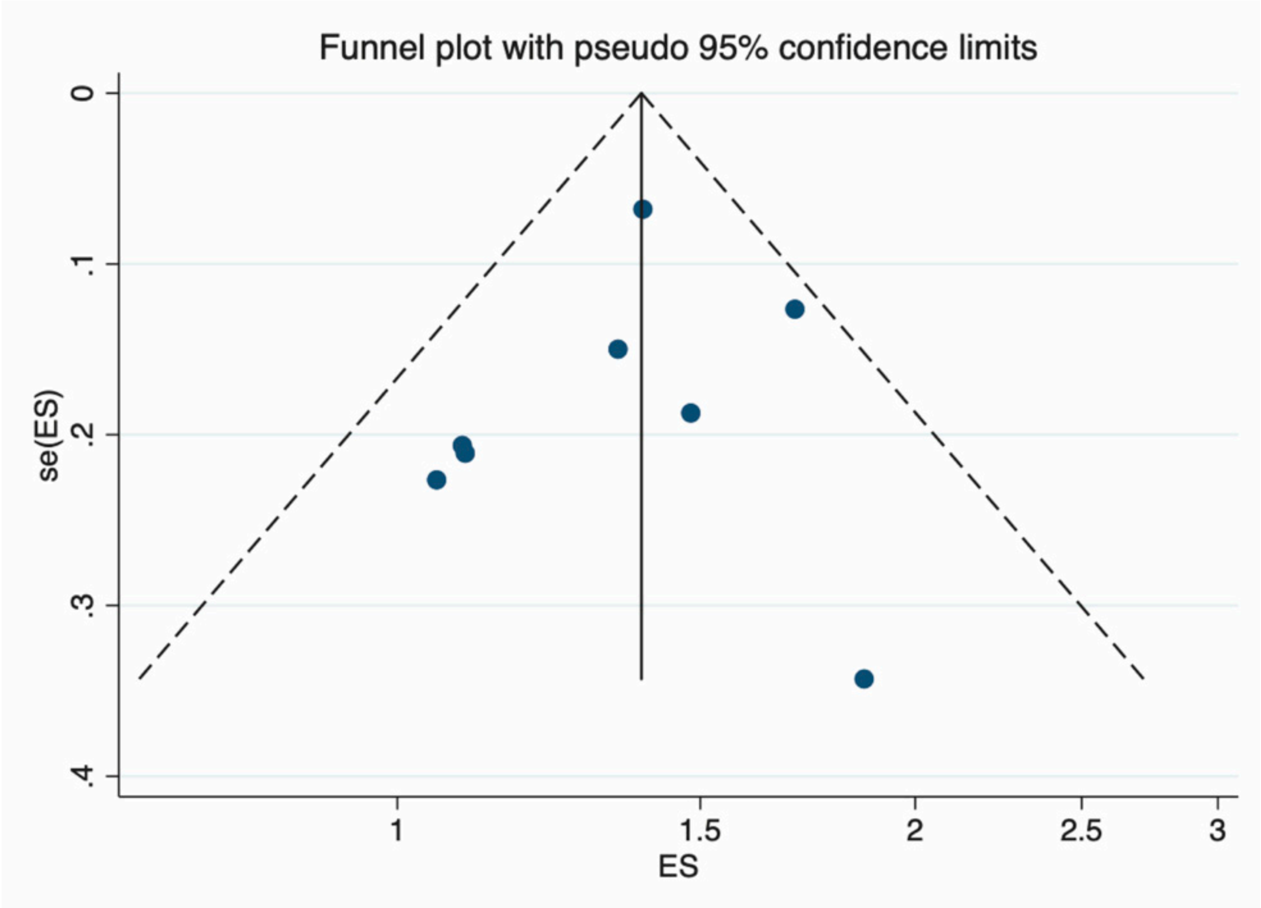

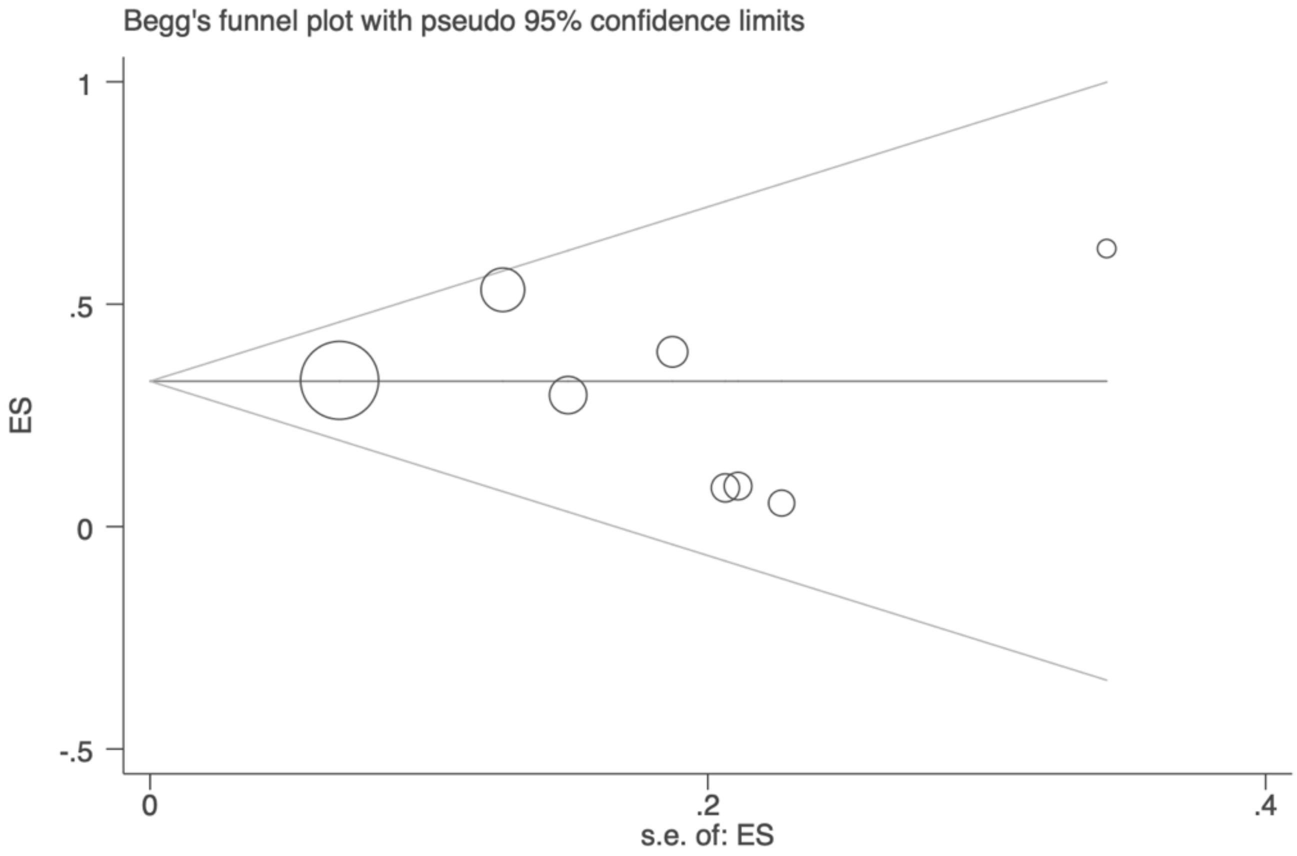

Begg's funnel plot with pseudo 95% confidence limits
1
.5
0
-.5
ES
0
.2
.4
s.e. of: ES

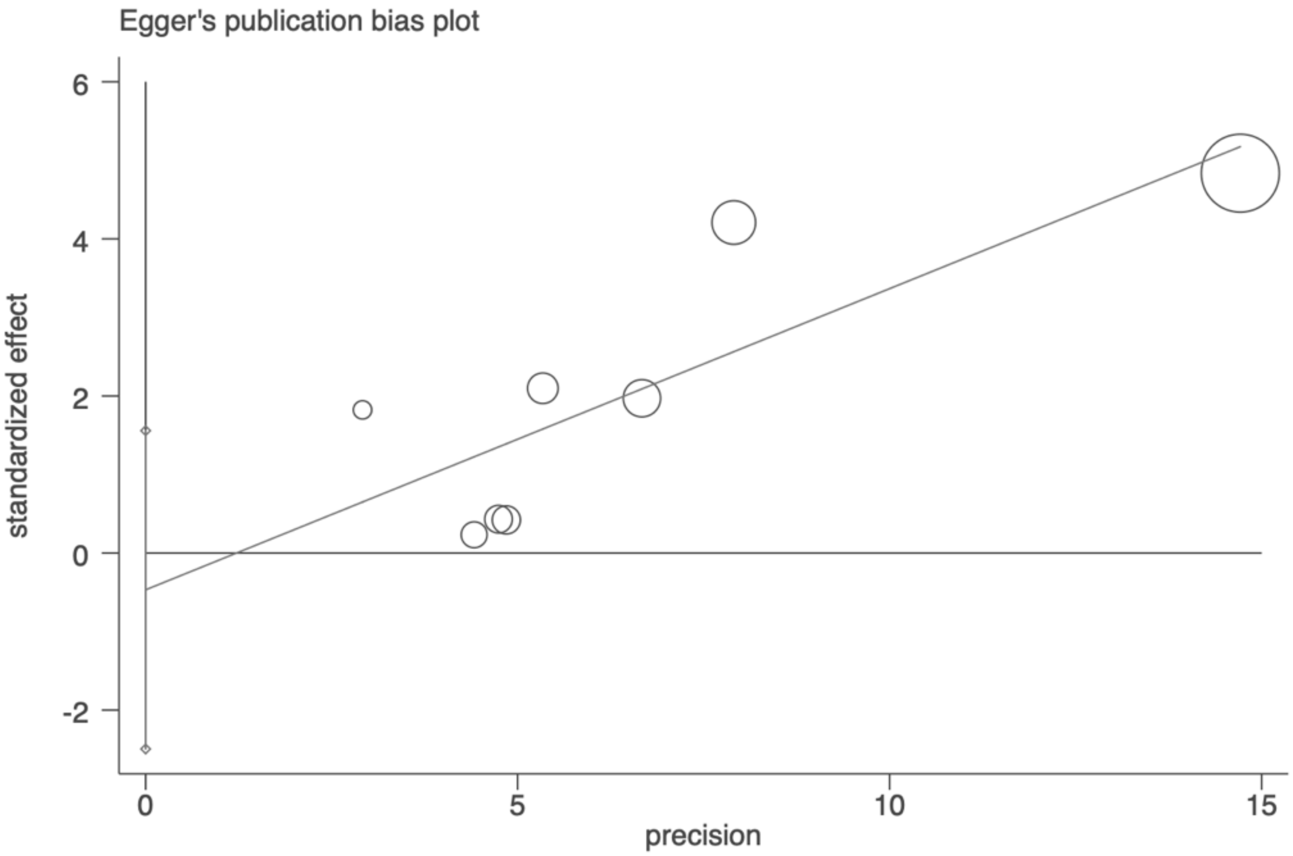

Egger's publication bias plot
standardized effect
precision
6
4
2
0
-2
0
5
10
15

Supplementary Figure S26: Publication bias of adverse events in Crohn's disease

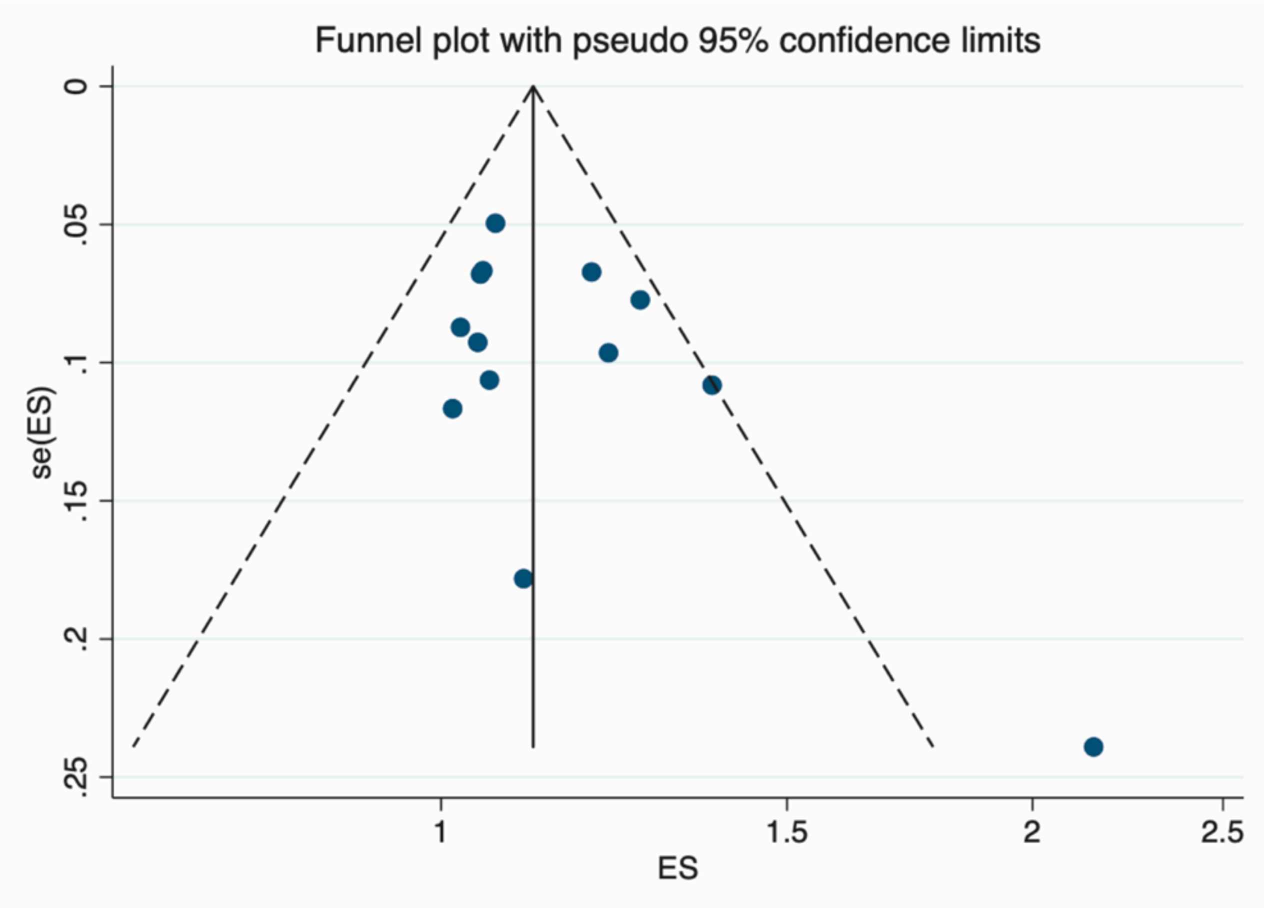

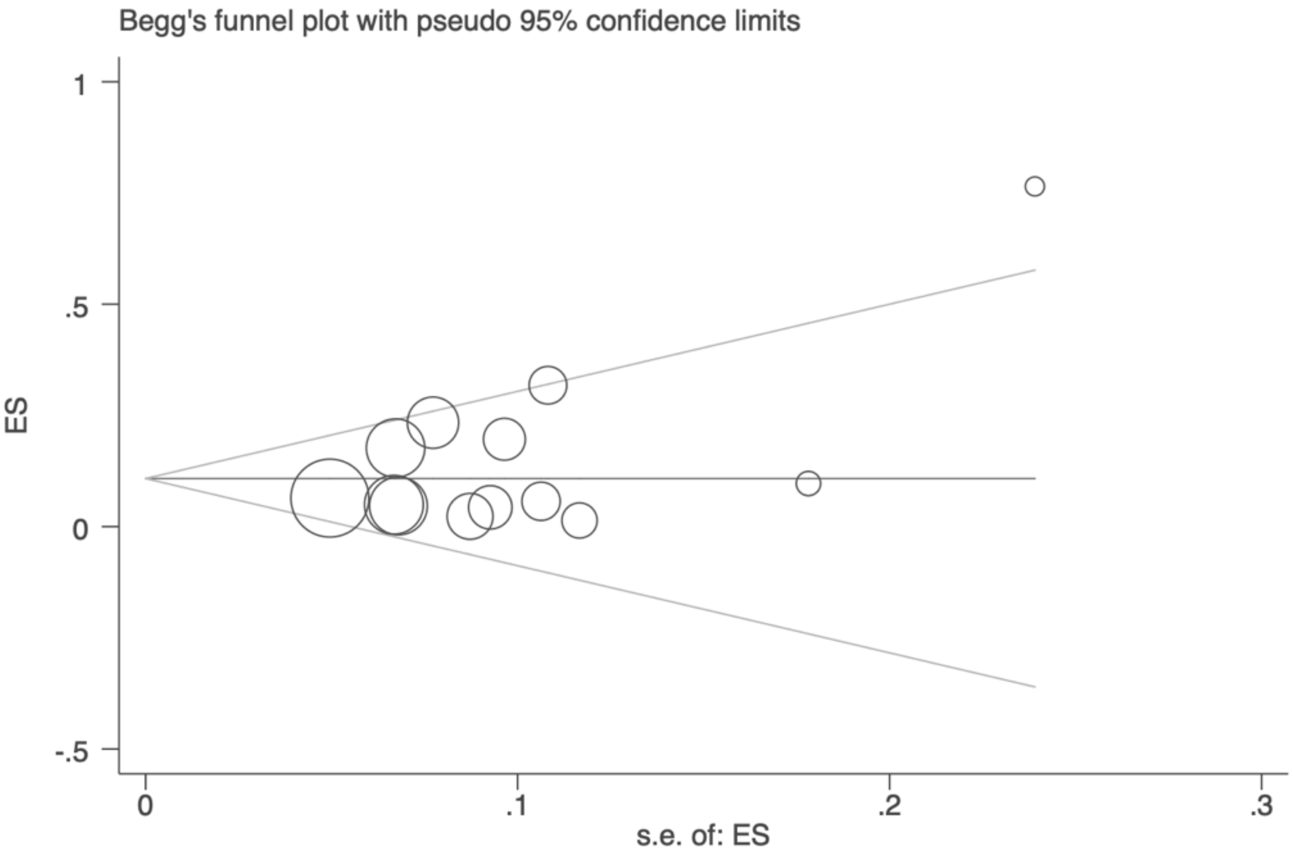

Begg's funnel plot with pseudo 95% confidence limits
1
.5
0
-.5
ES
0
.1
.2
.3
s.e. of: ES

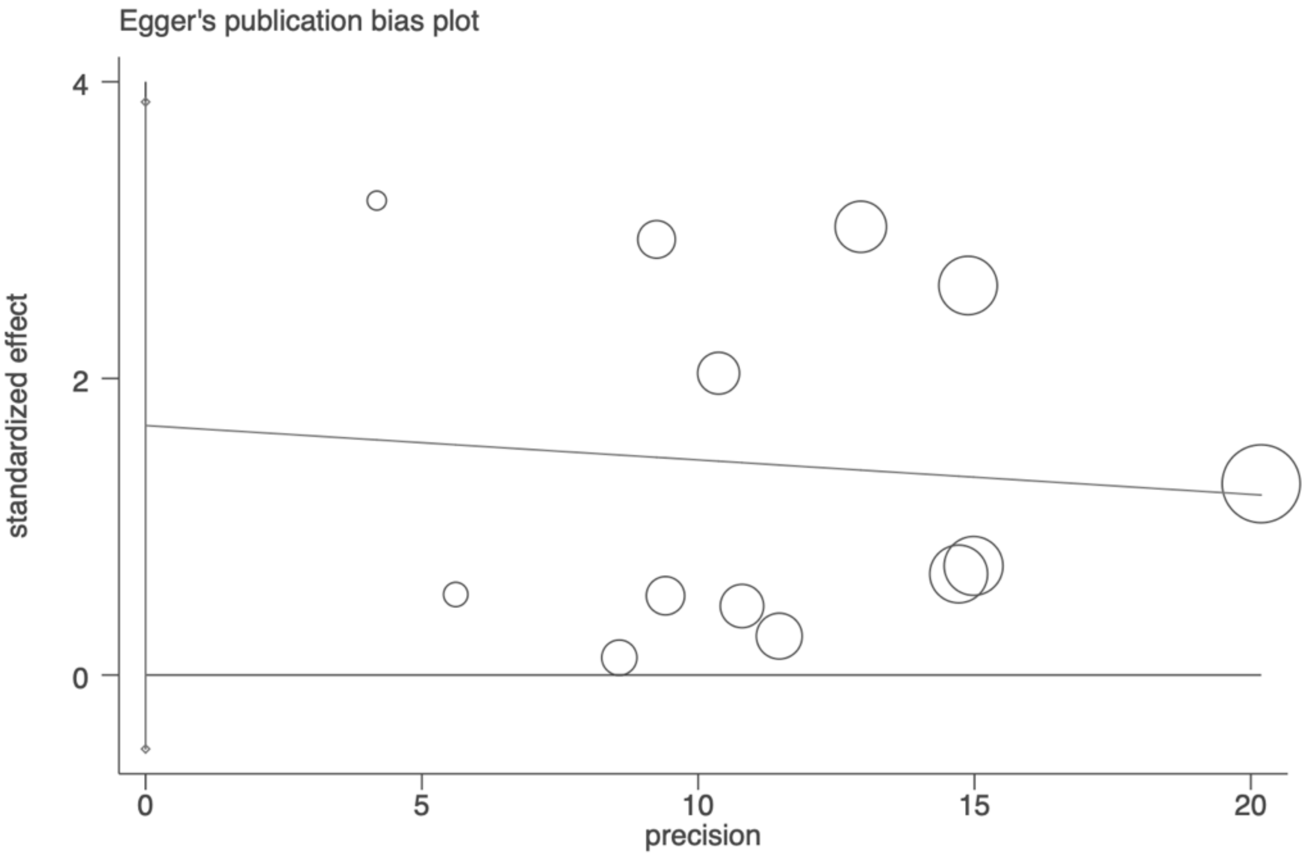
Egger's publication bias plot
standardized effect
4
2
0
0
5
10
15
20
precision
